# Copper-Catalyzed Three- Five- or Seven-Component Coupling Reactions: The Selective Synthesis of Cyanomethylamines, *N,N*-Bis(Cyanomethyl)Amines and *N,N'*-Bis(Cyanomethyl)Methylenediamines Based on a Strecker-Type Synthesis

**DOI:** 10.3390/molecules181012488

**Published:** 2013-10-10

**Authors:** Norio Sakai, Nobuaki Takahashi, Daiki Inoda, Reiko Ikeda, Takeo Konakahara

**Affiliations:** Department of Pure and Applied Chemistry, Faculty of Science and Technology, Tokyo University of Science (RIKADAI), Noda, Chiba 278-8510, Japan

**Keywords:** multi-component coupling, Strecker-type reaction, nitriles, copper

## Abstract

We have demonstrated that a cooperative catalytic system comprised of CuCl and Cu(OTf)_2_ could be used to effectively catalyse the three-, five- and seven-component coupling reactions of aliphatic or aromatic amines, formaldehyde, and trimethylsilyl cyanide (TMSCN), and selectively produce in good yields the corresponding cyanomethylamines, *N,N*-bis(cyanomethyl)amines and *N,N'*-bis(cyanomethyl)methylenediamines.

## 1. Introduction

Nitriles constitute a basic building block for natural products and biologically active substances and have been used as useful precursors of other functional groups, such as amines, amides and carboxylic acids [1,2,3,4,5], hence, the development of a facile and effective preparation of nitriles has attracted considerable attention in the field of synthetic chemistry. Thus far, the main demand has been satisfied by Strecker-type reactions using an amine, an aldehyde and an appropriate cyanide source [6,7,8,9,10,11,12,13,14,15,16,17,18,19]. However, the cyanide source used in conventional Strecker−type reactions is mainly limited to metal cyanides, such as sodium or potassium cyanide. As an extension, the preparation of dinitriles via a double Strecker reaction from primary amines, and using excess amounts of both paraformaldehyde and cyanide sources, has also been developed [20,21,22]. In recent, the use of trimethylsilyl cyanide (TMSCN), which is easy to handle and behaves as well as metal cyanides, has been developed and it has been extensively used as a nitrile surrogate [23].

In this context, we reported that a cooperative catalytic system comprised of CuCl and Cu(OTf)_2_ catalyzes a five-component coupling reaction of amines, formaldehyde, and TMSCN producing a dinitrile compound, a bis(cyanomethyl)amine derivative [24,25]. In the previous paper, the coupling reaction examined was limited to one example using trimethylsilyl cyanide. Thus, during our ongoing further extension on the five-component coupling reaction with the copper catalytic system and trimethylsilyl cyanide, we newly found that either the properties of the formaldehyde that was used in the reaction or the electronic effect of a substituent on an amine controlled a coupling mode of the amines, formaldehyde, and TMSCN, and selectively produced three-, five- or seven-component coupling products [26,27,28]. In this paper, we report on the detailed results of this unique multi-component coupling reaction, which is based on a Strecker-type reaction.

## 2. Results and Discussion

As a model reaction, when the reaction of *p*-methoxybenzylamine, a solid paraformaldehyde [29] (3 equiv.) and trimethylsilyl cyanide (TMSCN: 3 equiv.) was initially carried out with CuCl and Cu(OTf)_2_ in acetonitrile, an unexpected Strecker-type reaction occurred to produce the seven-component coupling adduct, *N,N'-*bis(cyanomethyl)methylenediamine derivative **1a** in a 61% yield within 10 min (entry 1 in Table 1) [30,31]. The structure of the amine **1a** was determined by its spectroscopic data. In addition, the use of a catalytic amount of a base promoted the seven−component coupling reaction, improving the product yield (entries 2–5). In particular, potassium carbonate afforded the selective formation of the seven−component coupling adduct **1a** (entry 4). Potassium *tert*−butoxide also shows a similar effect for the reaction (entry 5). It was noteworthy that when the reaction was performed with formalin (37 wt% aqueous solution), in contrast, the five−component coupling adduct, *N,N*-bis(cyanomethyl)amines **2a** was obtained selectively (entry 6). When an aqueous solution of the aldehyde that contained a much amount of monomer was used, the coupling form of three substrates was switched. For a five-component coupling reaction, without a base, the yield of the amine was enhanced to a nearly quantitative yield (entry 7). To show the utility of a cooperative catalytic system comprised of CuCl and Cu(OTf)_2_, the single use (10 mol%) of each copper catalyst was examined. When the reaction was conducted with either Cu(I) or Cu(II) under these conditions, it produced the seven−component coupling adduct, and in both cases the yield was reduced (entries 8 and 9). In addition, without both catalysts a substantial prolongation of the reaction time was observed (entry 10).

In general, the seven−coupling reaction with several aliphatic amines, paraformaldehyde and TMSCN, was carried out under optimal conditions (Table 2). In most cases, the expected seven−coupling reaction proceeded cleanly to selectively produce the corresponding *N,N'*−bis(cyanomethyl)methylenediamine derivatives **1** in moderate to good yields, but, with the exception of *p*−methylbenzyl amine, each reaction time was prolonged.

**Table 1:** Examination of reaction conditions.

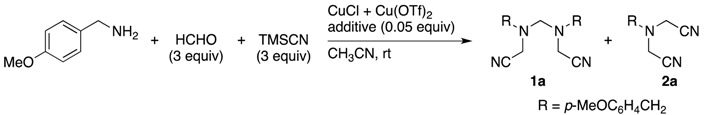						
Entry	Catalyst (mol%)	Aldehyde ^a^	Additive	Time	Yield	(%) ^b^
				(min)	1a	2a
1	CuCl (5) + Cu(OTf)_2_ (5)	PFA	−−−	10	61	ND
2	CuCl (5) + Cu(OTf)_2_ (5)	PFA	CaH_2_	10	85	ND
3	CuCl (5) + Cu(OTf)_2_ (5)	PFA	*t*−BuOK	10	90	ND
4	CuCl (5) + Cu(OTf)_2_ (5)	PFA	K_2_CO_3_	10	(99)	ND
5	CuCl (5) + Cu(OTf)_2_ (5)	PFA	Na_2_CO_3_	10	72	ND
6	CuCl (5) + Cu(OTf)_2_ (5)	formalin	K_2_CO_3_	10	ND	57
7	CuCl (5) + Cu(OTf)_2_ (5)	formalin	−−−	10	ND	(99)
8	CuCl (10)	PFA	K_2_CO_3_	10	65	ND
9	Cu(OTf)_2_ (10)	PFA	K_2_CO_3_	10	43	ND
10	None ^c^	PFA	K_2_CO_3_	120	54	ND

^a^ PFA: paraformaldehyde, formalin: formaldehyde in 37wt% aqueous solution. ^b^ NMR (Isolated) yield. ^c^ Without both copper catalysts.

**Table 2:** Selective synthesis of bis(cyanomethyl)methylenediamines **1** with paraformaldehyde *^a^*.

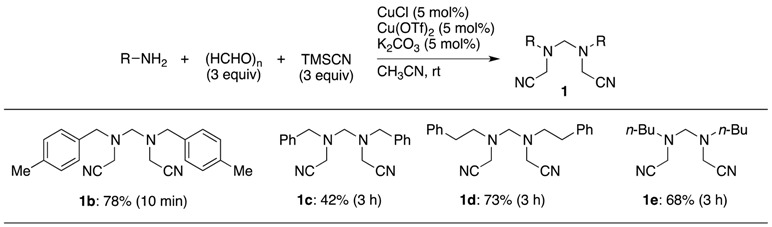

*^a^* Conditions: an amine (0.60 mmol), paraformaldehyde (1.80 mmol), TMSCN (1.80 mmol), Cu(OTf)_2_ (0.0300 mmol), CuCl (0.030 mmol) and K_2_CO_3_ (0.030 mmol) in CH_3_CN (0.6 mL).

Next, we performed selective preparations of a five-component coupling adduct with a formalin solution (Table 3). When the reaction with primary aliphatic amines was conducted using the optimal conditions shown in Table 1, the expected double Strecker products, the bis(cyanomethyl)amines **2b**–**e** was obtained selectively in excellent yields. The use of branched amines also produced the corresponding amines **2f**–**h** in good yields. It was noteworthy that this catalytic system successfully accommodated coupling reactions using an aliphatic amine with a hydroxy group.

**Table 3:** Selective synthesis of *N,N*-bis(cyanomethyl)amines **2** with formalin *^a^*.

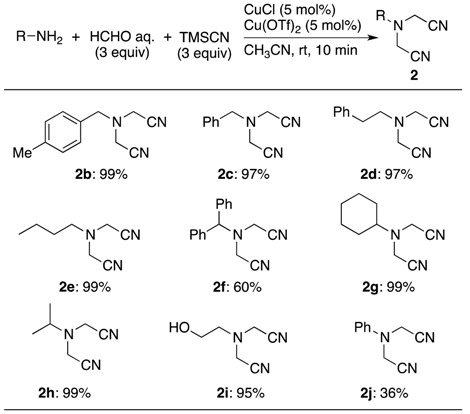

*^a^* Conditions: an amine (0.60 mmol), formalin (37 wt% aqueous solution) (1.80 mmol), TMSCN (1.80 mmol), Cu(OTf)_2_ (0.0300 mmol) and CuCl (0.030 mmol) in CH_3_CN (0.6 mL).

However, the catalytic system in acetonitrile could not be applied to the five−component coupling reaction of aromatic amines (see the result for **2j**). After many tests, the use of paraformaldehyde in methanol effected the desired five-component coupling reaction for aromatic amines, selectively affording the bis(cyanomethyl)amine derivatives **3a**–**d**, but this tendency was true only for an aromatic amine having an electron-donating group (Table 4). When the reaction was conducted with aniline, the product yield was drastically reduced to less than 12%. Surprisingly, when the reaction was carried out with an aromatic amine having a strong electron-withdrawing substituent, the typical Strecker-type products cyanomethylamines **4a**–**c** were obtained in good yields as sole products, even though the reaction time was prolonged to 24 h. This is probably due to the fact that the nucleophilicity of the formed secondary amine was drastically reduced by a strong electron-withdrawing group.

**Scheme 1:**
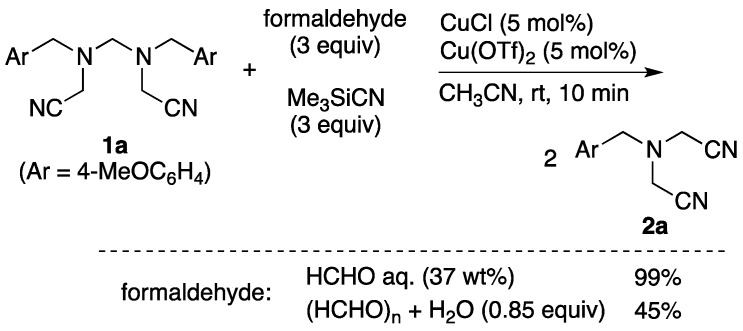
Conversion from bis(cyanomethyl)methylenediamines to bis(cyanomethyl)amine.

To understand the reaction pathway for the coupling reaction, several control experiments were conducted (Scheme 1). When the isolated *N,N'-*bis(cyanomethyl)methylenediamine **1a** was treated with the conditions that selectively gave a double Strecker product, amine **1a** was promptly and completely converted to bis(cyanomethyl)amine **2a**. Therefore, the result proved that a seven-component adduct acted as a part of the precursor of a five-component coupling adduct. Simultaneously, the result from this system showed that a retro-Mannich-type reaction had occurred. Also, when a similar reaction was carried out with a mixture of solid paraformaldehyde and an amount of water equals to that of the formalin solution used, the yield of the bis(cyanomethyl)amine was decreased to 45%. Consequently, this implied that the structure of the formed amines depended on the properties of the formaldehyde rather than on the presence of water. In other words, the use of formalin, which contains a relatively large amount of monomer, tended to produce the selective formation of five-component coupling adducts. In contrast, the employment of a solid paraformaldehyde, which was composed of a polymer, under dehydrating conditions, preferentially tended to produce the seven-component coupling adducts. However, the reaction conditions with aromatic amines shown in Table 3 were not suitable for comparison.

**Table 4:** Selective synthesis of bis(cyanomethyl)amines **3** and cyanomethylamines **4** with aromatic amines *^a^*.

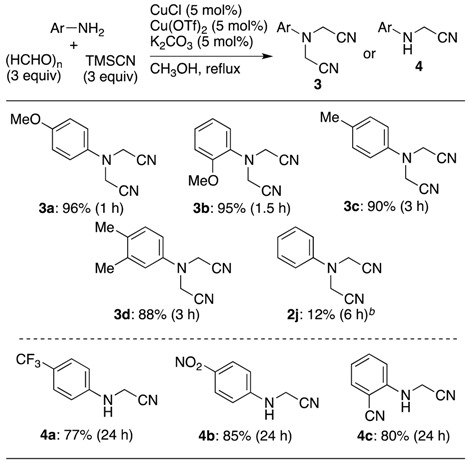

*^a^* Conditions: an amine (0.60 mmol), paraformaldehyde (1.80 mmol), TMSCN (1.80 mmol), Cu(OTf)_2_ (0.0300 mmol), CuCl (0.030 mmol) and K_2_CO_3_ (0.030 mmol) in CH_3_CN (0.6 mL) *^b^* Bis(cyanomethyl)amines **2j’** having a *p*−methoxymethylene group was obtained as a by−product.

**Scheme 2:**
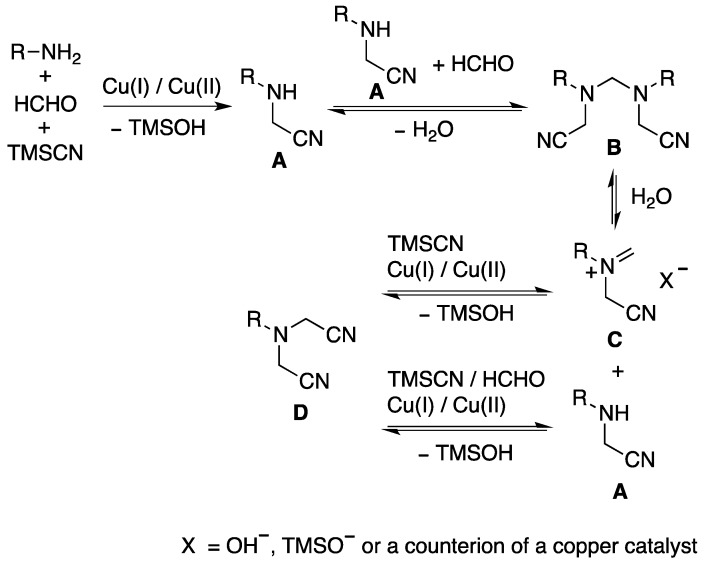
Plausible reaction pathway for the multi−component coupling reaction.

On the basis of the results, we present a plausible reaction pathway for the multi-component coupling reaction as shown in Scheme 2. In the first step, amine **A** was formed from an amine, formaldehyde, and TMSCN via a Strecker-type reaction. During this step, the role of copper triflate was presumably that of the activation of formaldehyde and a consequent generation of *N,O*-hemiacetal. Also, the copper chloride activated the trimethylsilyl cyanide to produce a cyanide anion. Then, two molecules of cyanomethylamine **A** were condensed with formaldehyde to afford the corresponding methylenediamine **B**, followed by a retro-Mannich reaction with the liberated water to form iminium intermediate **C** and amine **A** [32]. Both amine **A** and iminium **C** were again reacted with TMSCN and/or formaldehyde in the presence of copper catalysts to produce bis(cyanomethyl)amine **D**.

## 3. Experimental

### General

Column chromatography was performed using a silica gel. CH_3_CN was distilled from P_2_O_5_ and dried over MS4A, respectively. Copper(I) and copper(II) salts were commercial available, and used without a further purification. All reactions were carried out under a N_2_ atmosphere, unless otherwise noted. ^1^H-NMR spectra were measured at 500 (or 300) MHz using TMS (tetramethylsilane) as an internal standard. ^13^C-NMR spectra were measured at 125 (or 75) MHz using TMS or a center peak of chloroform (77.0 ppm) as an internal standard. High−resolution mass spectra were measured using NBA (3−nitrobenzylalcohol) as a matrix. A solid paraformaldehyde was purchased from Wako Pure Chemical Ind. (purity > 95%) and TIC (purity > 88%), respectively. Known compounds were identified by ^1^H-NMR and ^13^C-NMR by comparison with literature spectral data.

### General procedure for the synthesis of N,N'-bis(cyanomethyl)methylenediamines

To a CH_3_CN solution (0.6 mL) in a screw-capped vial under a N_2_ atmosphere, an amine (0.60 mmol), paraformaldehyde (1.80 mmol), trimethylsilyl cyanide (0.225 mL, 1.80 mmol), Cu(OTf)_2_ (10.8 mg, 0.0300 mmol), CuCl (3.0 mg, 0.030 mmol) and potassium carbonate (4.0 mg, 0.030 mmol) were successively added, and the vial was sealed with a cap containing a PTFE septum. The reaction mixture was stirred at room temperature. After the reaction, the mixture was directly subjected to SiO_2_ gel without the usual work-up, and was purified by flash column chromatography (hexane−AcOEt) to give the corresponding amines.

*2,2'-[Methylenebis(4-methoxybenzylimino)]bisacetonitrile* (**1a**): 217 mg (99%); Pale yellow oil; ^1^H-NMR (500 MHz, CDCl_3_) δ 3.47 (s, 2H), 3.55 (s, 4H), 3.72 (s, 4H), 3.81 (s, 6H), 6.88 (d, 2H, *J* = 8.5 Hz), 7.23 (d, 2H, *J* = 8.5 Hz); ^13^C-NMR (125 MHz, CDCl_3_) δ 38.6, 55.2, 55.3, 72.5, 114.1, 114.6, 128.2, 130.1, 159.4; MS(FAB): *m/z* 365 (M^+^+H); HRMS(FAB): Calcd for C_21_H_24_N_4_O_2_: 365.1978, found 365.1974.

*2,2'-[Methylenebis(4-methylbenzylimino)]bisacetonitrile* (**1b**): 156 mg (78%); Pale yellow oil; ^1^H-NMR (500 MHz, CDCl_3_) δ 2.35 (s, 6H), 3.47 (s, 2H), 3.56 (s, 4H), 3.74 (s, 4H), 7.15 (d, 2H, *J* = 8.0 Hz), 7.20 (d, 2H, *J* = 8.0 Hz); ^13^C-NMR (125 MHz, CDCl_3_) δ 21.1, 38.7, 55.5, 72.6, 114.6, 128.8, 129.4, 133.2, 137.7; MS(FAB): *m/z* 333 (M^+^+H); HRMS(FAB): Calcd for C_21_H_24_N_4_: 333.2079, found 333.2051.

*2,2'-[Methylenebis(benzylimino)]bisacetonitrile* [26] (**1c**): 76 mg (42%); pale yellow oil; ^1^H-NMR (500 MHz, CDCl_3_) δ 3.50 (s, 2H), 3.58 (s, 4H), 3.79 (s, 4H), 7.35 (m, 10H); ^13^C-NMR (125 MHz, CDCl_3_) δ 38.9, 55.8, 72.6, 114.6, 128.0, 128.8, 128.9, 136.2.

*2,2'-[Methylenebis(2-phenylethyllimino)]bisacetonitrile* (**1d**): 145 mg (73%); Pale yellow oil; ^1^H-NMR (500 MHz, CDCl_3_) δ 2.74 (t, 4H, *J* = 7.0 Hz), 2.85 (t, 4H, *J* = 7.0 Hz), 3.28 (s, 2H), 3.44 (s, 4H), 7.15 (d, 4H, *J* = 6.5 Hz), 7.21 (t, 2H, *J* = 6.5 Hz), 7.29 (t, 4H, *J* = 6.5 Hz); ^13^C-NMR (125 MHz, CDCl_3_) δ 33.7, 39.0, 52.6, 73.0, 115.0, 126.4, 128.5, 128.5, 139.0; MS(FAB): *m/z* 331 (M^+^−H); HRMS(FAB): Calcd for C_21_H_23_N_4_: 331.1923 (M^+^−H), found 331.1917.

*2,2'-[Methylenebis(buthylimino)]bisacetonitrile* [26] (**1e**): 96 mg (68%); Pale yellow oil; ^1^H-NMR (500 MHz, CDCl_3_) δ 2.74 (t, 4H, *J* = 7.0 Hz), 2.85 (t, 4H, *J* = 7.0 Hz), 3.28 (s, 2H), 3.44 (s, 4H), 7.15 (d, 4H), 7.21 (t, 2H), 7.29 (t, 4H); ^13^C-NMR (125 MHz, CDCl_3_) δ 13.8, 20.2, 29.3, 39.2, 51.4, 72.9, 115.0.

### General procedure for the synthesis of N,N-bis(cyanomethyl)amines using an aliphatic amine

To a CH_3_CN solution (0.6 mL) in a screw-capped vial under a N_2_ atmosphere, an aliphatic amine (0.60 mmol), a formaldehyde aqueous solution (0.135 mL, 1.80 mmol), trimethylsilyl cyanide (0.225 mL, 1.80 mmol), Cu(OTf)_2_ (10.8 mg, 0.0300 mmol) and CuCl (3.0 mg, 0.030 mmol) was successively added, and the vial was sealed with a cap containing a PTFE septum. The reaction mixture was stirred at room temperature. After the reaction, the mixture was directly subjected to SiO_2_ gel without the usual extraction, and was purified by flash column chromatography (hexane-AcOEt) to give the corresponding *N,N*-bis(cyanomethyl)amines.

*N-(4-Methoxybenzyl)-N,N-bis(cyanomethyl)amine* (**2a**): 128 mg (99%); Pale yellow oil; ^1^H-NMR (500 MHz, CDCl_3_) δ 3.58 (s, 4H), 3.73 (s, 2H), 3.82 (s, 3H), 6.90 (d, 2H, *J* = 8.5 Hz), 7.26 (d, 2H, *J* = 8.5 Hz); ^13^C-NMR (125 MHz, CDCl_3_) δ 41.4, 55.3, 57.5, 114.2, 114.4, 126.7, 130.4, 159.8; MS(EI): *m/z* 215; HRMS(FAB): Calcd for C_12_H_14_N_3_O: 216.1137, found 216.1145.

*N-(4-Methylbenzyl)-N,N-bis(cyanomethyl)amine* (**2b**): 119 mg (99%); Pale yellow oil; ^1^H-NMR (500 MHz, CDCl_3_) δ 2.36 (s, 3H), 3.58 (s, 4H), 3.74 (s, 2H), 7.18 (d, 2H, *J* = 8.0 Hz) 7.22 (d, 2H, *J* = 8.0 Hz); ^13^C-NMR (125 MHz, CDCl_3_) δ 21.1, 41.5, 57.7, 114.2, 129.0, 129.6, 131.7, 138.5; MS(EI): *m/z* 199; HRMS(FAB): Calcd for C_12_H_14_N_3_: 200.1188, found 200.1190.

*N-Benzyl-N,N-bis(cyanomethyl)amine* [24] (**2c**): 107 mg (97%); Colorless oil; ^1^H-NMR (500 MHz, CDCl_3_) δ 3.60 (s, 4H), 3.79 (s, 2H), 7.33−7.39 (m, 5H); ^13^C-NMR (125 MHz, CDCl_3_) δ 41.5, 57.8, 114.2, 128.5, 128.8, 128.9, 134.7; MS(EI): *m/z* 185.

*N-(2-Phenylethyl)-N,N-bis(cyanomethyl)amine* (**2d**): 116 mg (97%); Pale yellow oil; ^1^H-NMR (500 MHz, CDCl_3_) δ 2.80 (t, 2H, *J* = 7.5 Hz), 2.91 (t, 2H, *J* = 7.5 Hz), 3.60 (s, 4H), 7.19 (d, 2H, *J* = 7.5 Hz), 7.24 (t, 1H, *J* = 7.5 Hz), 7.31 (t, 2H, *J* = 7.5 Hz); ^13^C-NMR (125 MHz, CDCl_3_) δ 33.5, 42.1, 54.8, 114.2, 126.7, 128.5, 128.6, 137.9; MS(EI): *m/z* 198 (M^+^−H); HRMS(FAB): Calcd for C_12_H_12_N_3_: 198.1031 (M^+^−H), found 198.1014.

*N-Butyl-N,N-bis(cyanomethyl)amine* [33] (**2e**): 90 mg (99%); Pale yellow oil; ^1^H-NMR (500 MHz, CDCl_3_) δ 0.94 (t, 3H, *J* = 7.5 Hz), 1.37 (sext, 2H, *J* = 7.5 Hz), 1.49 (quint, 2H, *J* = 7.5 Hz), 2.65 (t, 2H, *J* = 7.5 Hz), 3.61 (s, 4H); ^13^C-NMR (125 MHz, CDCl_3_) δ 13.7, 19.9, 29.0, 42.0, 53.4, 114.3; MS(EI): *m/z* 151.

*N-Benzhydryl-N,N-bis(cyanomethyl)amine* (**2f**): 94 mg (60%); Pale yellow oil; ^1^H-NMR (500 MHz, CDCl_3_) δ 3.64 (s, 4H), 4.67 (s, 1H), 7.27 (t, 2H, *J* = 7.5 Hz), 7.34 (t, 4H, *J* = 7.5 Hz), 7.47 (d, 4H, *J* = 7.5 Hz); ^13^C-NMR (125 MHz, CDCl_3_) δ 40.6, 71.8, 114.4, 127.4, 128.5, 129.4, 139.4; MS(FAB): *m/z* 262 (M^+^+H); HRMS(FAB): Calcd for C_17_H_16_N_3_: 262.1344, found 262.1353.

*N-Cyclohexyl-N,N-bis(cyanomethyl)amine* [34] (**2g**): 90 mg (99%); Pale yellow oil; ^1^H-NMR (500 MHz, CDCl_3_) δ 1.17 (m, 1H), 1.29 (m, 4H), 1.65 (m, 1H), 1.81 (m, 2H,), 1.93 (m, 2H), 2.63 (m, 1H), 3.72 (s, 1H); ^13^C-NMR (125 MHz, CDCl_3_) δ 24.8, 25.3, 29.8, 39.2, 60.3, 115.4; MS(EI): *m/z* 151.

*N-iso-Propyl-N,N-bis(cyanomethyl)amine* [33] (**2h**): 81 mg (99%); pale yellow oil; ^1^H-NMR (300 MHz, CDCl_3_) δ 1.18 (d, 6H, *J* = 6.5 Hz), 3.05 (sept, 1H, *J* = 6.5 Hz), 3.69 (s, 4H); ^13^C-NMR (75 MHz, CDCl_3_) δ 19.7, 39.1, 52.8, 115.3; MS(EI): *m/z* 137.

*N-(2-Hydroxyethyl)-N,N-bis(cyanomethyl)amine* [35] (**2i**): 79 mg (95%); Pale yellow oil; ^1^H-NMR (500 MHz, CDCl_3_) δ 2.02 (brs, 1H), 2.88 (t, 2H, *J* = 5.0 Hz), 3.75 (s, 4H), 3.80 (t, 2H, *J* = 5.0 Hz); ^13^C-NMR (125 MHz, CDCl_3_) δ 42.9, 53.1, 66.1, 114.5; MS(EI): *m/z* 139.

*N-Phenyl-N,N-bis(cyanomethyl)amine* [36] (**2j**): 37 mg (36%); pale yellow solid; mp. 159–160°C ^1^H-NMR (500 MHz, CDCl_3_) δ 4.24 (s, 3H), 7.00 (d, 2H, *J* = 7.5 Hz), 7.11 (t, 1H, *J* = 7.5 Hz) , 7.39 (t, 1H, *J* = 7.5 Hz); ^13^C-NMR (125 MHz, CDCl_3_) δ 41.0, 114.6, 117.2, 123.4, 130.0, 145.4; MS(EI): *m/z* 171.

*N-[4-(Methoxymethyl)phenyl]-N,N-bis(cyanomethyl)amine***(2j'**): 6% yield; Pale yellow oil; ^1^H-NMR (500 MHz, CDCl_3_) δ 3.39 (s, 3H), 4.24 (s, 4H), 4.42 (s, 2H), 6.97 (d, 2H, *J* = 9.0 Hz), 7.35 (d, 2H, *J* = 9.0 Hz); ^13^C-NMR (125 MHz, CDCl_3_) δ 41.0, 58.1, 73.9, 114.5, 117.1, 129.5, 133.8, 144.9; HRMS(FAB): Calcd for C_12_H_13_N_3_O: 215.1059, found 215.1032.

### General procedure for the synthesis of N,N-bis(cyanomethyl)amines using an aromatic amine

To a CH_3_CN solution (0.6 mL) in a screw-capped vial under a N_2_ atmosphere, an amine (0.60 mmol), paraformaldehyde (1.80 mmol), trimethylsilyl cyanide (0.225 mL, 1.80 mmol), Cu(OTf)_2_ (10.8 mg, 0.0300 mmol), CuCl (3.0 mg, 0.030 mmol) and potassium carbonate (4.0 mg, 0.030 mmol) were successively added, and the vial was sealed with a cap containing a PTFE septum. The reaction mixture was stirred at room temperature. After the reaction, the mixture was directly subjected to SiO_2_ gel without the usual work-up, and was purified by flash column chromatography (hexane-AcOEt) to give the corresponding amines.

*N-(4-Methoxyphenyl)-N,N-bis(cyanomethyl)amine* [24] (**3a**): 119 mg (99%); white solid; mp 114–115 °C; ^1^H-NMR (500 MHz, CDCl_3_) δ 3.80 (s, 3H), 4.13 (s, 4H), 6.91 (d, 2H, *J* = 9.0 Hz), 7.04 (d, 2H, *J* = 9.0 Hz); ^13^C-NMR (125 MHz, CDCl_3_) δ 42.2, 55.5, 114.7, 115.1, 121.0, 139.3, 156.8; MS(EI): *m/z* 201.

*N-(2-Methoxyphenyl)-N,N-bis(cyanomethyl)amine* (**3b**): 119 mg (99%); White solid; mp 61–62 °C; ^1^H-NMR (500 MHz, CDCl_3_) δ 3.90 (s, 3H), 4.18 (s, 4H), 6.94 (d, 1H, *J* = 7.5 Hz), 6.97 (t, 1H, *J* = 7.5 Hz), 7.09 (d, 1H, *J* = 7.5 Hz), 7.18 (t, 1H, *J* = 7.5 Hz); ^13^C-NMR (125 MHz, CDCl_3_) δ 41.2, 55.6, 112.0, 115.1, 121.3, 121.7, 126.7, 134.7, 152.9; MS(EI): *m/z* 201; HRMS(FAB): Calcd for C_11_H_11_N_3_O: 201.0902, found 201.0916.

*N-(4-Methylphenyl)-N,N-bis(cyanomethyl)amine* [36] (**3c**): 100 mg (90%); Yellow solid; mp 119–120 °C; ^1^H-NMR (500 MHz, CDCl_3_) δ 2.32 (s, 3H), 4.18 (s, 4H), 6.91 (d, 2H, *J* = 8.0 Hz), 7.17 (d, 2H, *J* = 8.0 Hz); ^13^C-NMR (125 MHz, CDCl_3_) δ 20.5, 41.3, 114.7, 117.8, 130.4, 133.5, 143.2; MS(EI): *m/z* 185.

*N-(3,4-Dimethylphenyl)-N,N-bis(cyanomethyl)amine* (**3d**): 105 mg (88%); white solid; mp 136–137 °C; ^1^H-NMR (500 MHz, CDCl_3_) δ 2.22 (s, 3H), 2.27 (s, 3H), 4.18 (s, 4H), 6.74 (d, 1H, *J* = 8.0 Hz), 7.79 (s, 1H), 7.11(d, 1H, *J* = 8.0 Hz); ^13^C-NMR (125 MHz, CDCl_3_) δ 18.9, 20.1, 41.3, 114.7, 115.2, 119.3, 130.8, 132.2, 138.3, 143.6; MS(EI): *m/z* 199: HRMS(FAB): Calcd for C_12_H_13_N_3_:199.1109, found 199.1082.

*N-[4-(Trifluoromethyl)phenyl]-N-(cyanomethyl)amine* [37] (**4a**): 92 mg (77%); White solid; mp 106 °C; ^1^H-NMR (500 MHz, CDCl_3_) δ 4.13 (s, 2H), 4.39 (brs, 1H), 6.72 (d, 2H, *J* = 8.5 Hz), 7.50 (d, 2H, *J* = 8.5 Hz); ^13^C-NMR (125 MHz, CDCl_3_) δ 32.1, 112.8. 116.3, 121.7 (q, ^2^*J*_C−F_ = 32.6 Hz), 124.5 (q, ^1^*J*_C−F_ = 271.2 Hz), 126.9 (q, ^3^*J*_C−F_ = 3.4 Hz), 147.6; MS(EI): *m/z* 200.

*N-(4-Nitrophenyl)-N-(cyanomethyl)amine* [20] (**4b**): 90 mg (85%); Yellow solid; mp 112–113 °C; ^1^H-NMR (500 MHz, DMSO−d_6_) δ 4.44 (d, 2H, *J* = 4.5Hz), 6.81 (d, 2H, *J* = 9.0 Hz), 7.6 (t, 1H, *J* = 4.5 Hz), 8.08 (d, 1H, *J* = 9.0 Hz); ^13^C-NMR (125 MHz, DMSO−d_6_) δ 30.9, 112.0, 117.6, 126.1, 137.9, 152.6; MS(EI): *m/z* 177.

N-(2-cyanophenyl)-N-(cyanomethyl)amine [38] (**4c**): 75 mg (80%); White solid; mp 95–96 °C; ^1^H-NMR (500 MHz, CDCl_3_) δ 4.20 (d, 2H, *J* = 6.5 Hz), 5.21(brs, 1H), 6.80 (d, 1H, *J* = 7.5 Hz), 6.87 (t, 1H, *J* = 7.5 Hz), 7.48 (d, 1H, *J* = 7.5 Hz), 7.52 (t, 1H, *J* = 7.5 Hz); ^13^C-NMR (125 MHz, CDCl_3_) δ 31.6, 97.6, 111.0, 115.9, 117.1, 119.1, 133.1, 134.6, 147.6.

## 4. Conclusions

We have demonstrated that a cooperative catalytic system comprised of CuCl and Cu(OTf)_2_ effectively catalyzes a three-, five- or seven-component coupling reaction of aliphatic amines, formaldehyde, and TMSCN under neutral or basic conditions to produce cyanomethylamines, *N,N*-bis(cyanomethyl)amines, and *N,N'*-bis(cyanomethyl)methylenediamines, respectively. We also have found that a seven-component adduct functioned as a part of the precursor of a five-component coupling adduct in a multi-component coupling reaction series.

## Supplementary Materials

Supplementary materials can be accessed at: http://www.mdpi.com/1420-3049/18/10/12488/s1.
