# Supplementary material for: Copper-Catalyzed Three- Five- or Seven-Component Coupling Reactions: The Selective Synthesis of Cyanomethylamines, N,N-Bis(Cyanomethyl)Amines and N,N'-Bis(Cyanomethyl)Methylenediamines Based on a Strecker-Type Synthesis
Source: Molecules. 2013 Oct 10;18(10):12488–99. doi: 10.3390/molecules181012488 (PMC6270086; doi:10.3390/molecules181012488)
Supplement: Supplementary file 1 [file molecules-18-12488-s001.pdf]

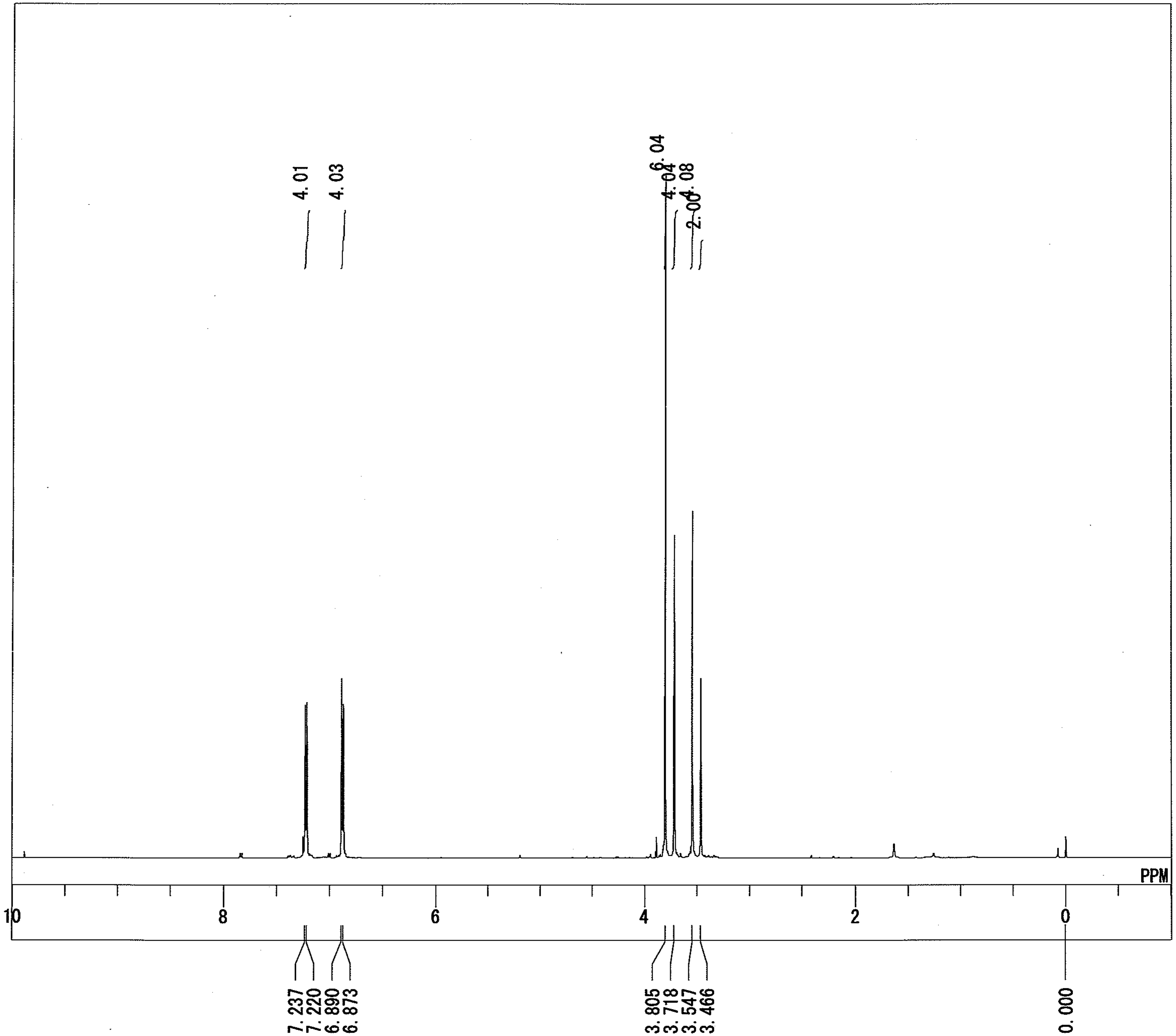

COMNT	Single Pulse Experiment
DATIM	26-05-2009 18:22:40
OBNUC	1H
EXMOD	single_pulse.exp
OBFRQ	500.16 MHz
OBSET	2.41 KHz
OBFIN	6.01 Hz
POINT	32768
FREQU	7507.51 Hz
SCANS	8
ACQTM	2.1823 sec
PD	4.0000 sec
PW1	7.00 usec
IRNUC	
CTEMP	24.5 c
SLVNT	CDCL3
EXREF	0.00 ppm
BF	0.23 Hz
RGAIN	12

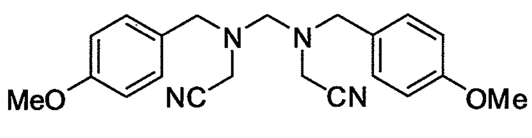

1a

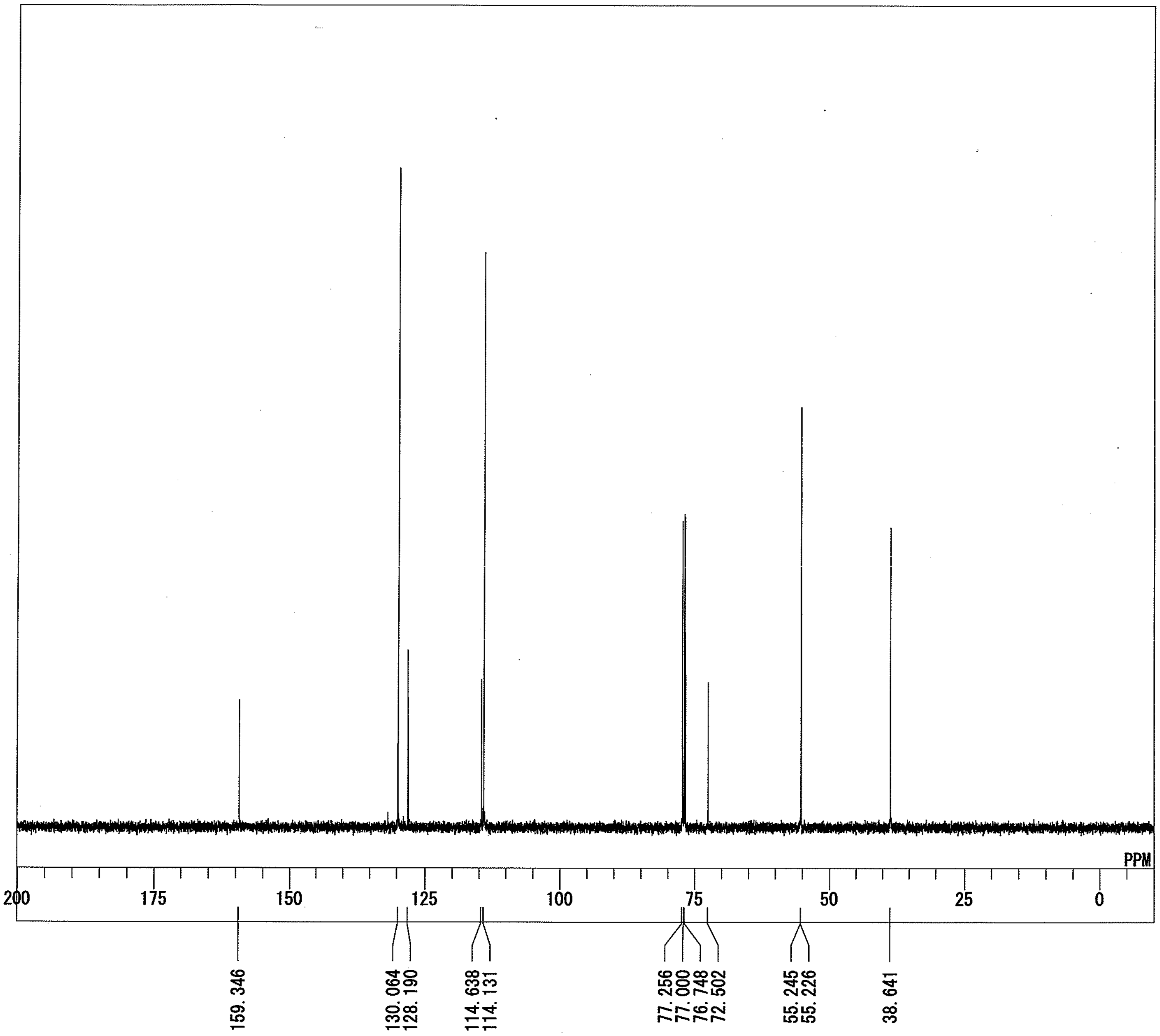

COMNT	Single Pulse with Broadband Decoupling
DATIM	26-05-2009 18:30:08
OBNUC	13C
EXMOD	single_pulse_dec
OBFRQ	125.77 MHz
OBSET	7.87 KHz
OBFIN	4.21 Hz
POINT	65536
FREQU	31446.54 Hz
SCANS	191
ACQTM	1.0420 sec
PD	1.0000 sec
PW1	4.17 usec
IRNUC	1H
CTEMP	27.0 c
SLVNT	CDCL3
EXREF	77.00 ppm
BF	1.00 Hz
RGAIN	30

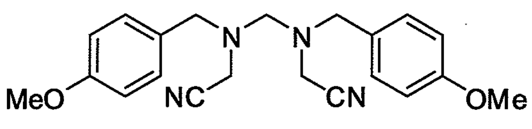

1a

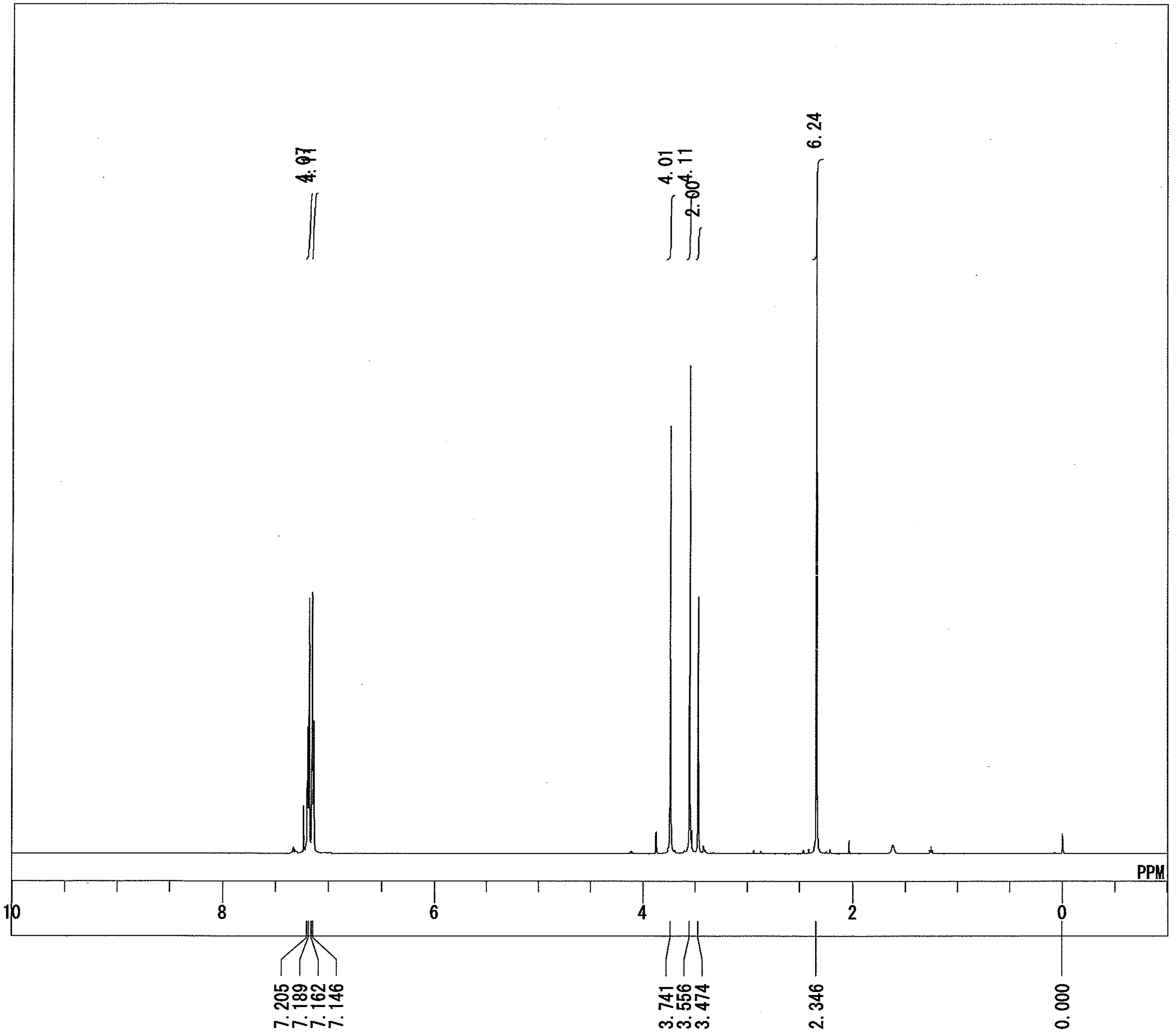

COMNT Single Pulse Experiment
DATIM 11-11-2009 19:58:16
OBNUC 1H
EXMOD single_pulse.exp
OBFRQ 500.16 MHz
OBSET 2.41 KHz
OBFIN 6.01 Hz
POINT 32768
FREQU 7507.51 Hz
SCANS 8
ACQTM 2.1823 sec
PD 4.0000 sec
PW1 7.00 usec
IRNUC
CTEMP 22.5 c
SLVNT CDCL3
EXREF 0.00 ppm
BF 0.23 Hz
RGAIN 12
4.07
4.11
4.01
4.11
2.00
6.24
PPM
10
8
6
4
2
0
7.205
7.189
7.162
7.146
3.741
3.556
3.474
2.346
0.000

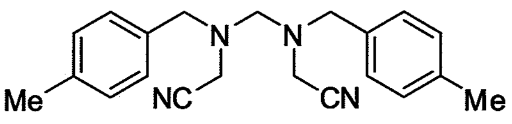

Me
NC
N
N
CN
Me

1b

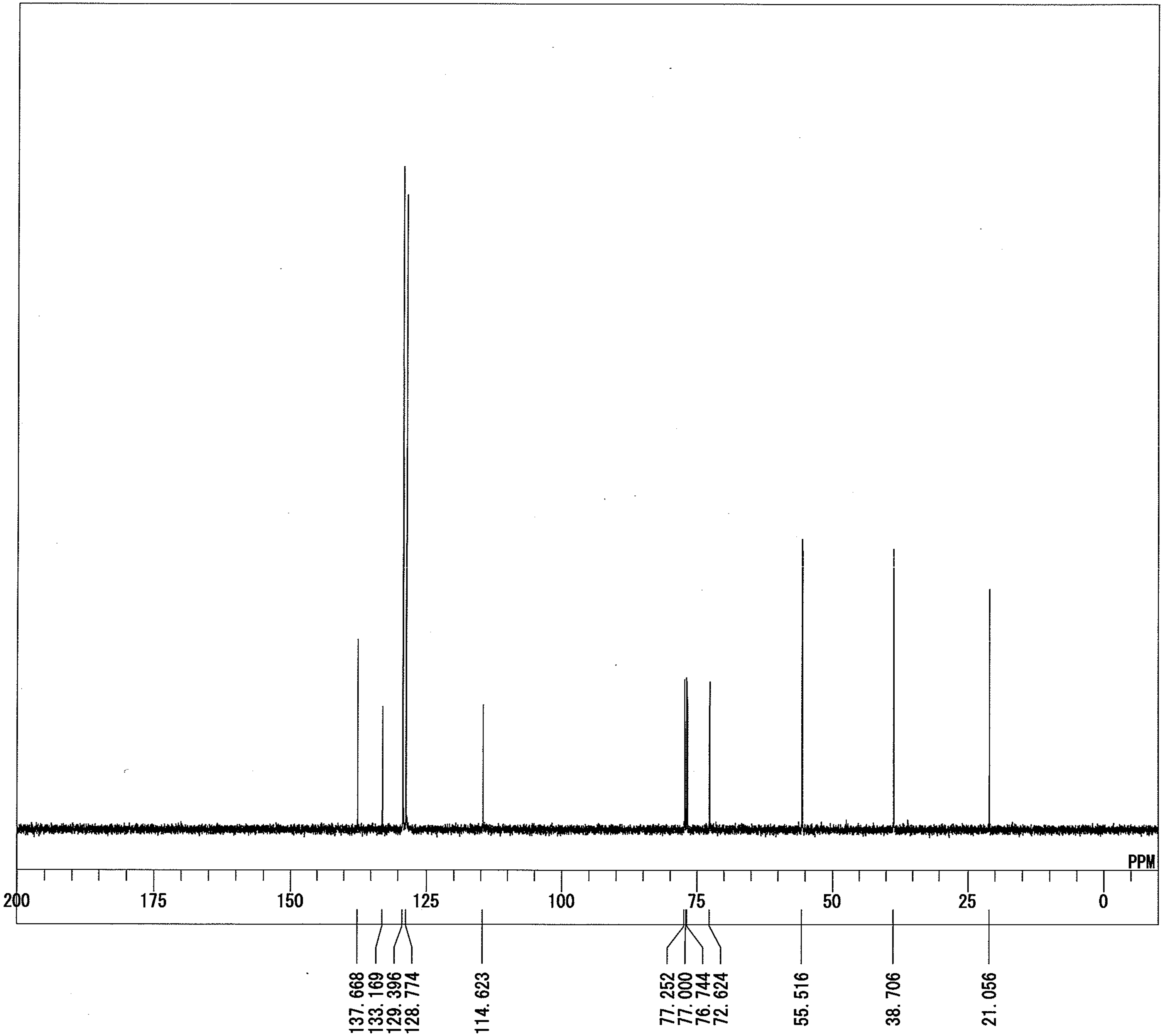

COMNT Single Pulse with Broadband Decoupling
DATIM 11-11-2009 20:05:26
OBNUC 13C
EXMOD single_pulse_dec
OBFRQ 125.77 MHz
OBSET 7.87 KHz
OBFIN 4.21 Hz
POINT 65536
FREQU 31446.54 Hz
SCANS 185
ACQTM 1.0420 sec
PD 1.0000 sec
PW1 4.17 usec
IRNUC 1H
CTEMP 25.9 c
SLVNT CDCL3
EXREF 77.00 ppm
BF 1.00 Hz
RGAIN 30

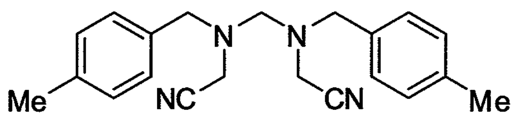

1b

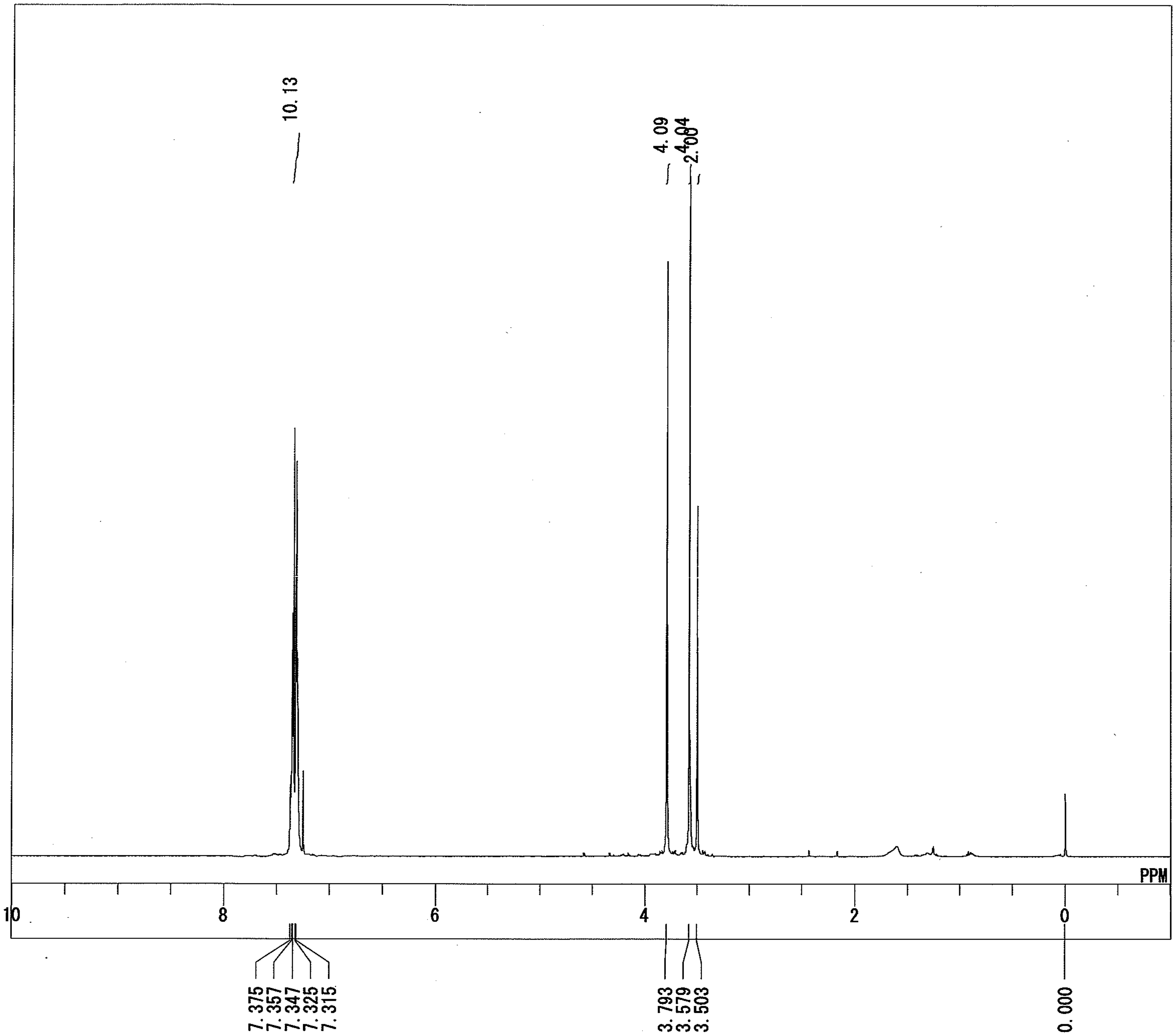

COMNT	Single Pulse Experiment
DATIM	13-12-2010 13:00:06
OBNUC	1H
EXMOD	single_pulse.exp
OBFRQ	500.16 MHz
OBSET	2.41 KHz
OBFIN	6.01 Hz
POINT	32768
FREQU	7507.51 Hz
SCANS	8
ACQTM	2.1823 sec
PD	4.0000 sec
PW1	7.00 usec
IRNUC	
CTEMP	21.8 c
SLVNT	CDCL3
EXREF	0.00 ppm
BF	0.23 Hz
RGAIN	15

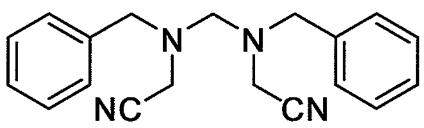

1c

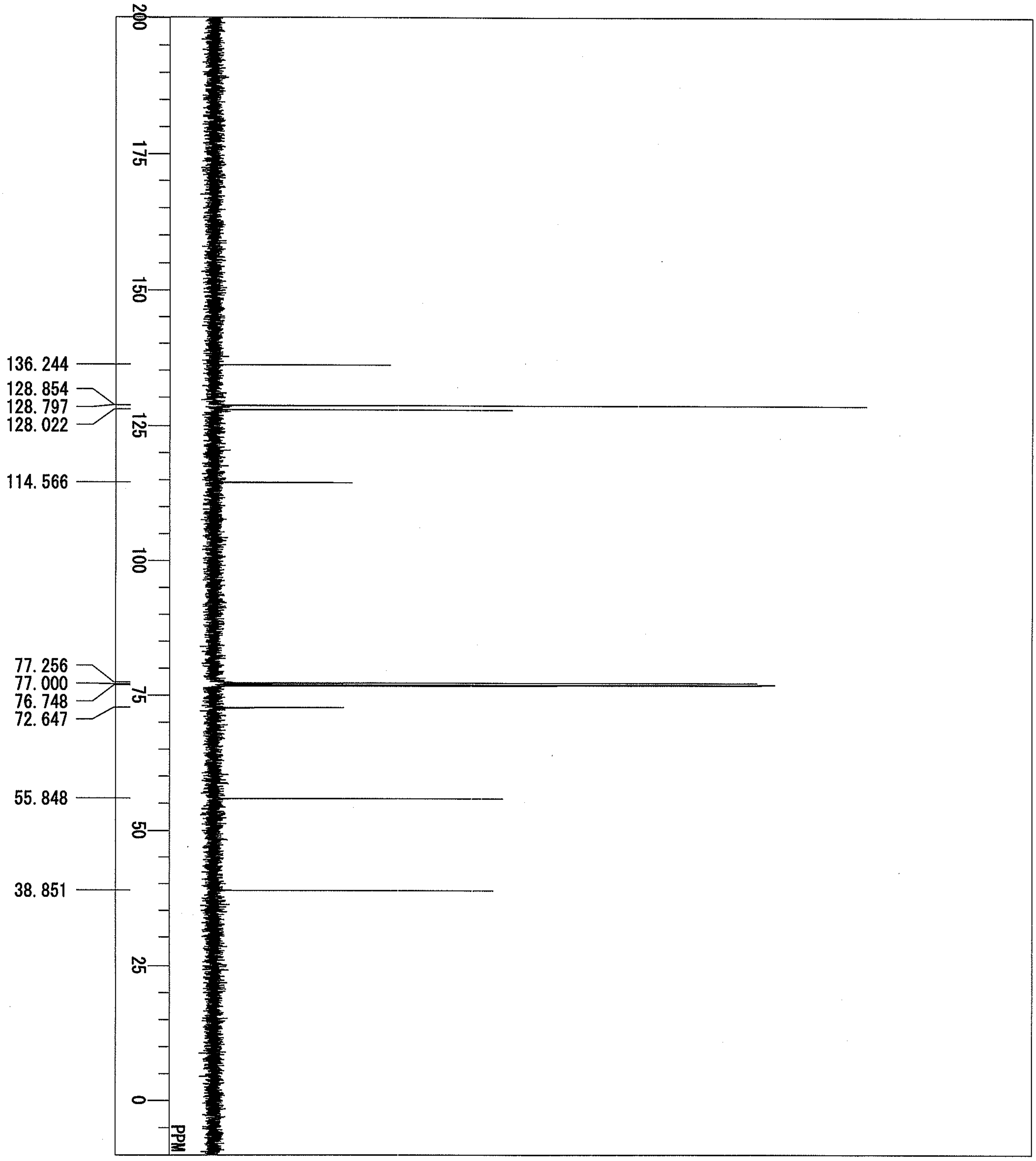

COMNT Single Pulse with Broadband Decoupling
DATIM 13-12-2010 13:14:29
OBNUC 13C
EXMOD single_pulse_dec
OBFRQ 125.77 MHz
OBSET 7.87 KHz
OBFIN 4.21 Hz
POINT 65536
FREQU 31446.54 Hz
SCANS 262
ACQTM 1.0420 sec
PD 1.0000 sec
PW1 4.17 usec
IRNUC 1H
CTEMP 22.9 c
SLVNT CDCL3
EXREF 77.00 ppm
BF 1.00 Hz
RGAIN 30

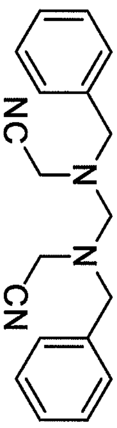

1c

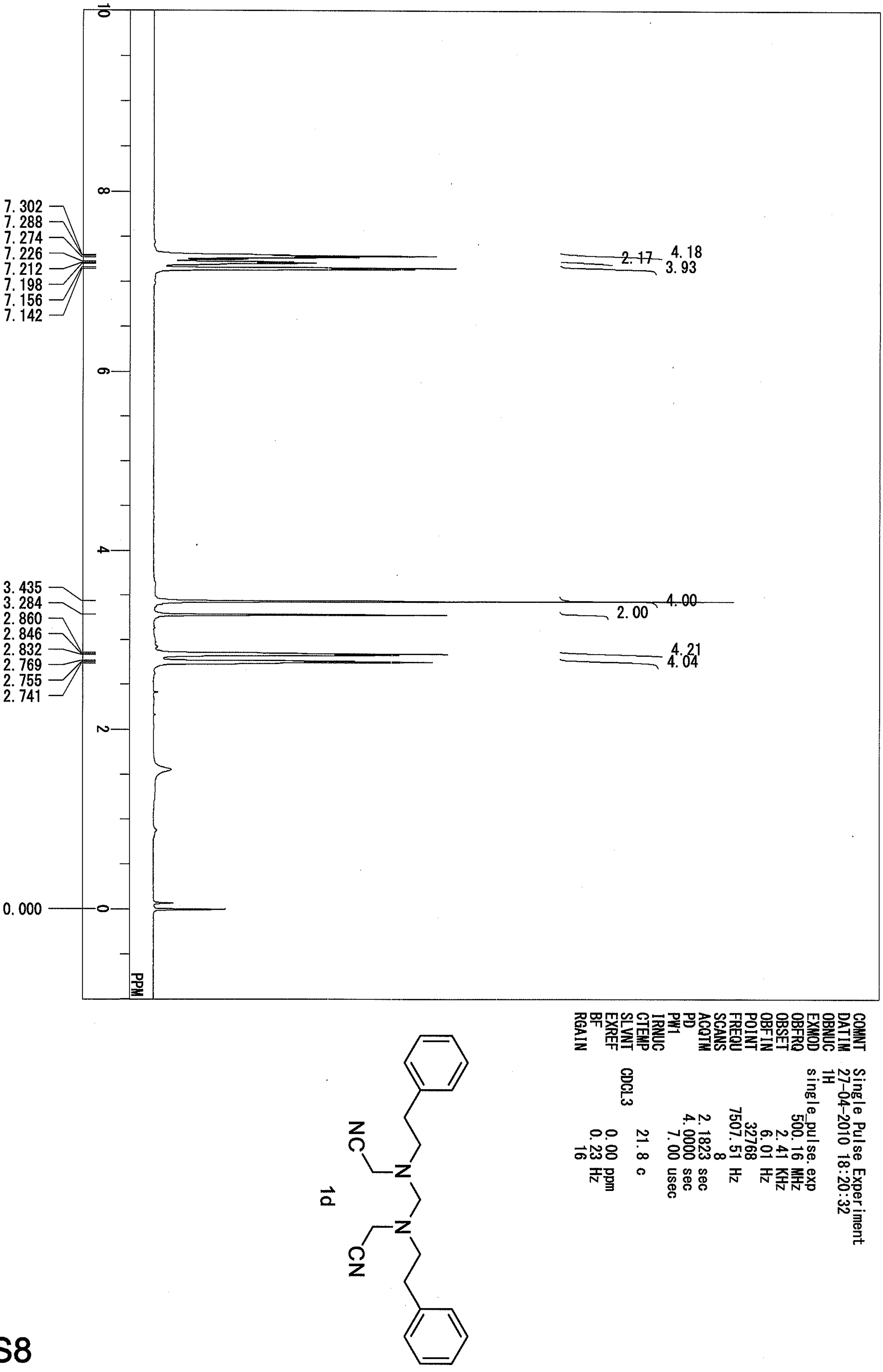
7.302
7.288
7.274
7.226
7.212
7.198
7.156
7.142
3.435
3.284
2.860
2.846
2.832
2.769
2.755
2.741
0.000
PPM
2.17
4.18
3.93
4.00
2.00
4.21
4.04
COMNT Single Pulse Experiment
DATIM 27-04-2010 18:20:32
OBNUC 1H
EXMOD single_pulse.exp
OBFRQ 500.16 MHz
OBSET 2.41 KHz
OBFIN 6.01 Hz
POINT 32768
FREQU 7507.51 Hz
SCANS 8
ACQTM 2.1823 sec
PD 4.0000 sec
PW1 7.00 usec
IRNUC
CTEMP 21.8 c
SLVNT CDCL3
EXREF 0.00 ppm
BF 0.23 Hz
RGAIN 16
NC
N
N
CN
1d

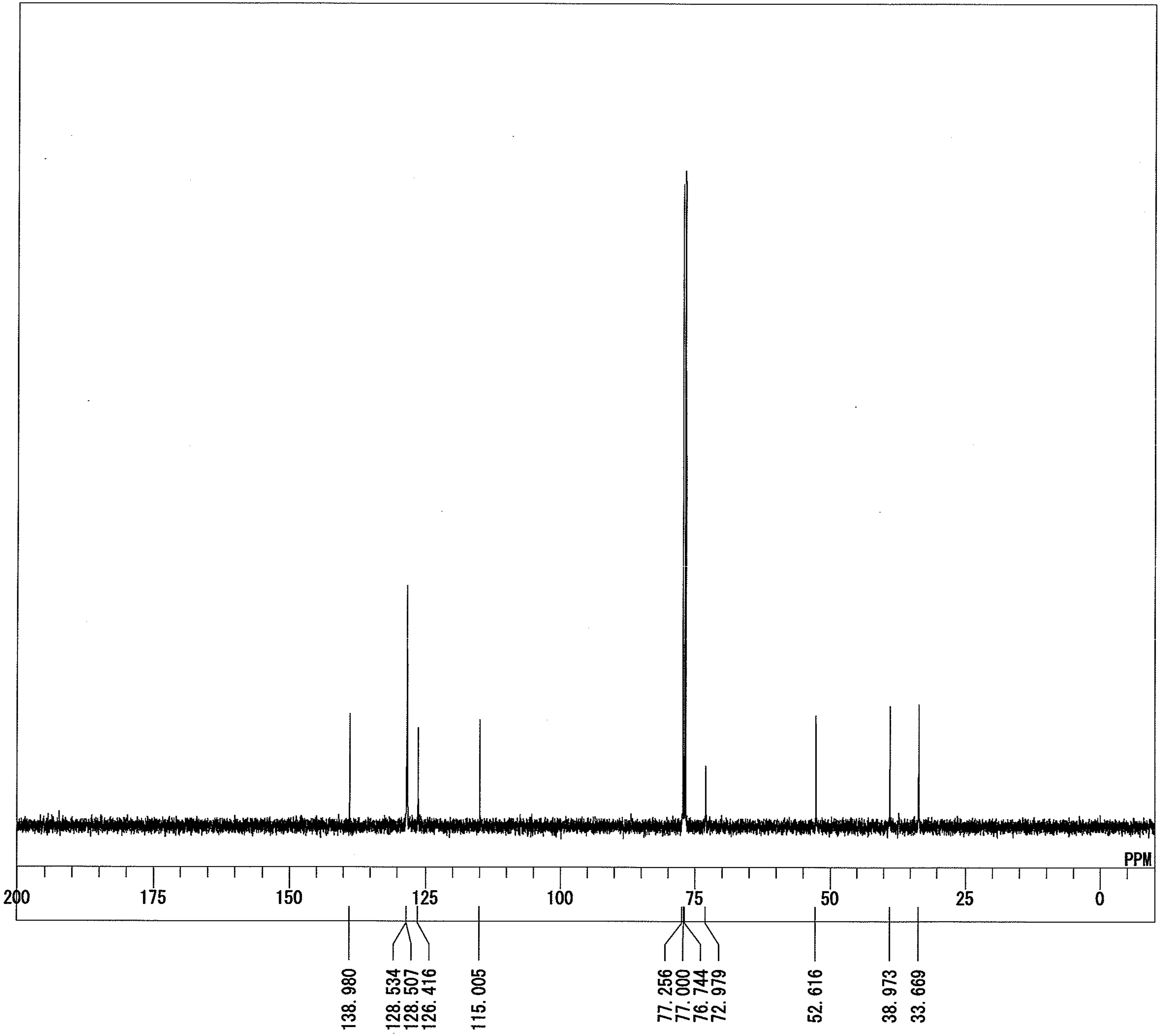

COMNT	Single Pulse with Broadband Decoupling
DATIM	27-04-2010 18:45:55
OBNUC	13C
EXMOD	single_pulse_dec
OBFRQ	125.77 MHz
OBSET	7.87 KHz
OBFIN	4.21 Hz
POINT	65536
FREQU	31446.54 Hz
SCANS	659
ACQTM	1.0420 sec
PD	1.0000 sec
PW1	4.17 usec
IRNUC	1H
CTEMP	23.4 c
SLVNT	CDCL3
EXREF	77.00 ppm
BF	1.00 Hz
RGAIN	30

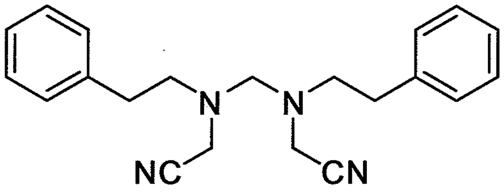

1d

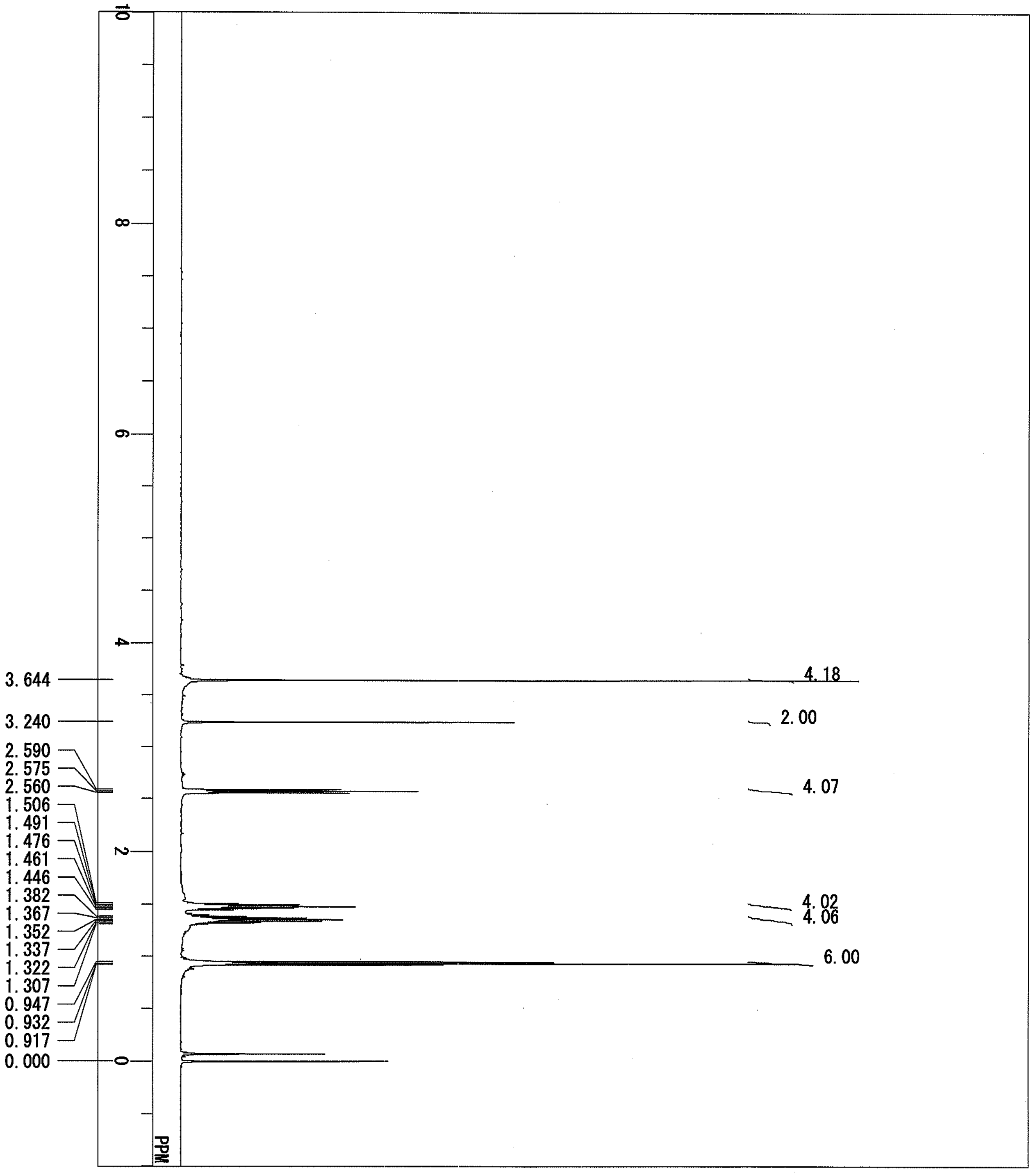
3.644
3.240
2.590
2.575
2.560
1.506
1.491
1.476
1.461
1.446
1.382
1.367
1.352
1.337
1.322
1.307
0.947
0.932
0.917
0.000
10
8
6
4
2
0
PPM
4.18
2.00
4.07
4.02
4.06
6.00
COMNT Single Pulse Experiment
DATIM 04-02-2011 13:01:43
OBNUC 1H
EXMOD single_pulse.exp
OBFRQ 500.16 MHz
OBSET 2.41 KHz
OBFIN 6.01 Hz
POINT 32768
FREQU 7507.51 Hz
SCANS 8
ACQTM 2.1823 sec
PD 4.0000 sec
PW1 7.00 usec
IRNUC
CTEMP 21.7 c
SLVNT CDCL3
EXREF 0.00 ppm
BF 0.23 Hz
RGAIN 18

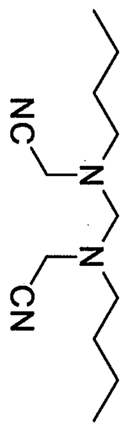
NC
N
N
CN

1e

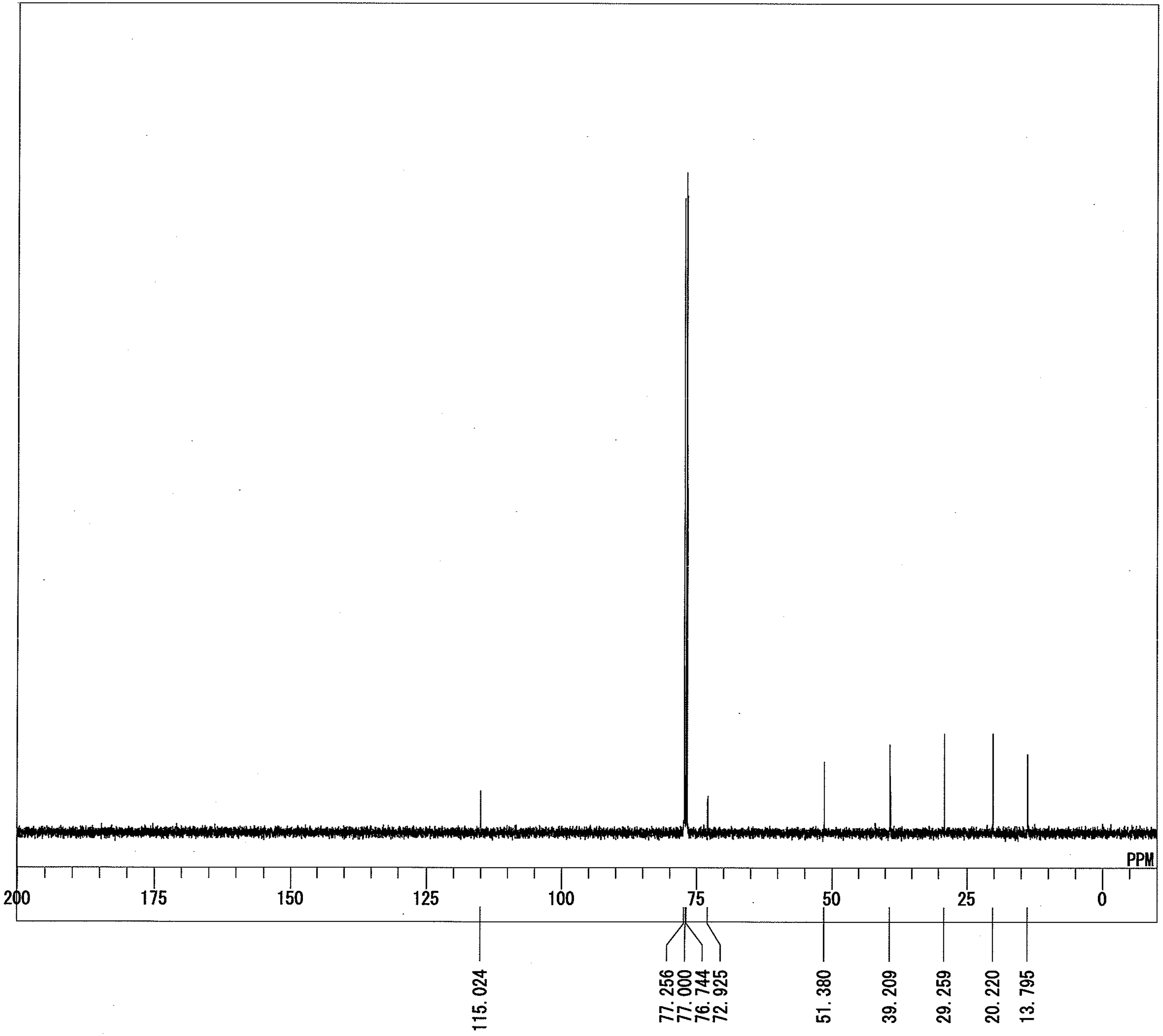

COMNT Single Pulse with Broadband Decoupling
DATIM 04-02-2011 13:33:18
OBNUC 13C
EXMOD single_pulse_dec
OBFRQ 125.77 MHz
OBSET 7.87 KHz
OBFIN 4.21 Hz
POINT 65536
FREQU 31446.54 Hz
SCANS 818
ACQTM 1.0420 sec
PD 1.0000 sec
PW1 4.17 usec
IRNUC 1H
CTEMP 24.1 c
SLVNT CDCL3
EXREF 77.00 ppm
BF 1.00 Hz
RGAIN 30

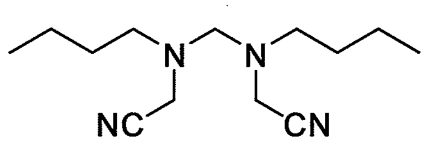

1e

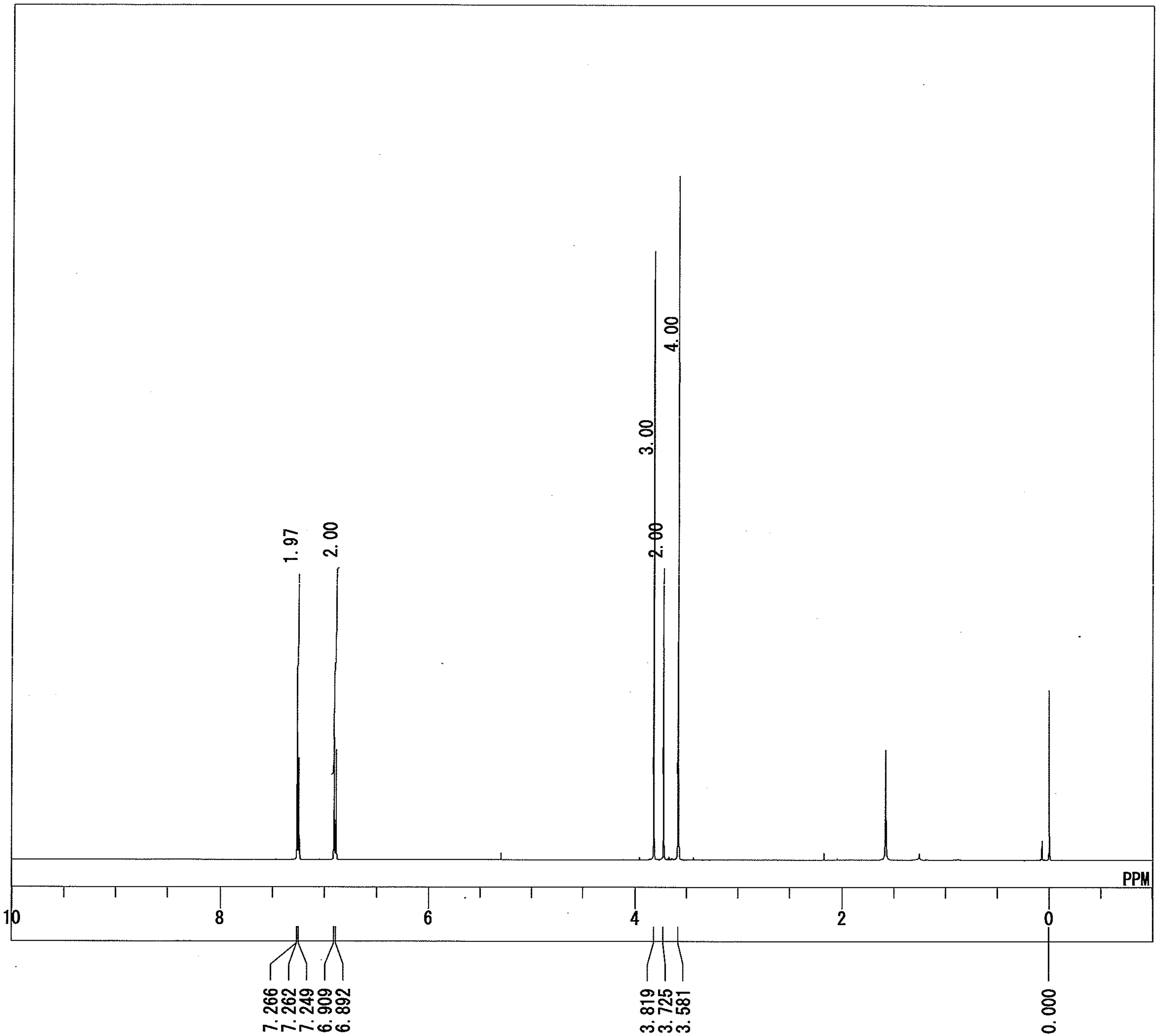

DFILE	DI-58-t-1-1H.als	
COMNT	Single Pulse Experiment	
DATIM	25-10-2008 17:18:38	
OBNUC	1H	
EXMOD	single_pulse.exp	
OBFRQ	500.16	MHz
OBSET	2.41	KHz
OBFIN	6.01	Hz
POINT	32768	
FREQU	7507.51	Hz
SCANS	8	
ACQTM	2.1823	sec
PD	4.0000	sec
PW1	7.00	usec
IRNUC		
CTEMP	24.5	c
SLVNT	CDCL3	
EXREF	0.00	ppm
BF	0.23	Hz
RGAIN	19	

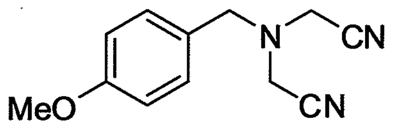

2a

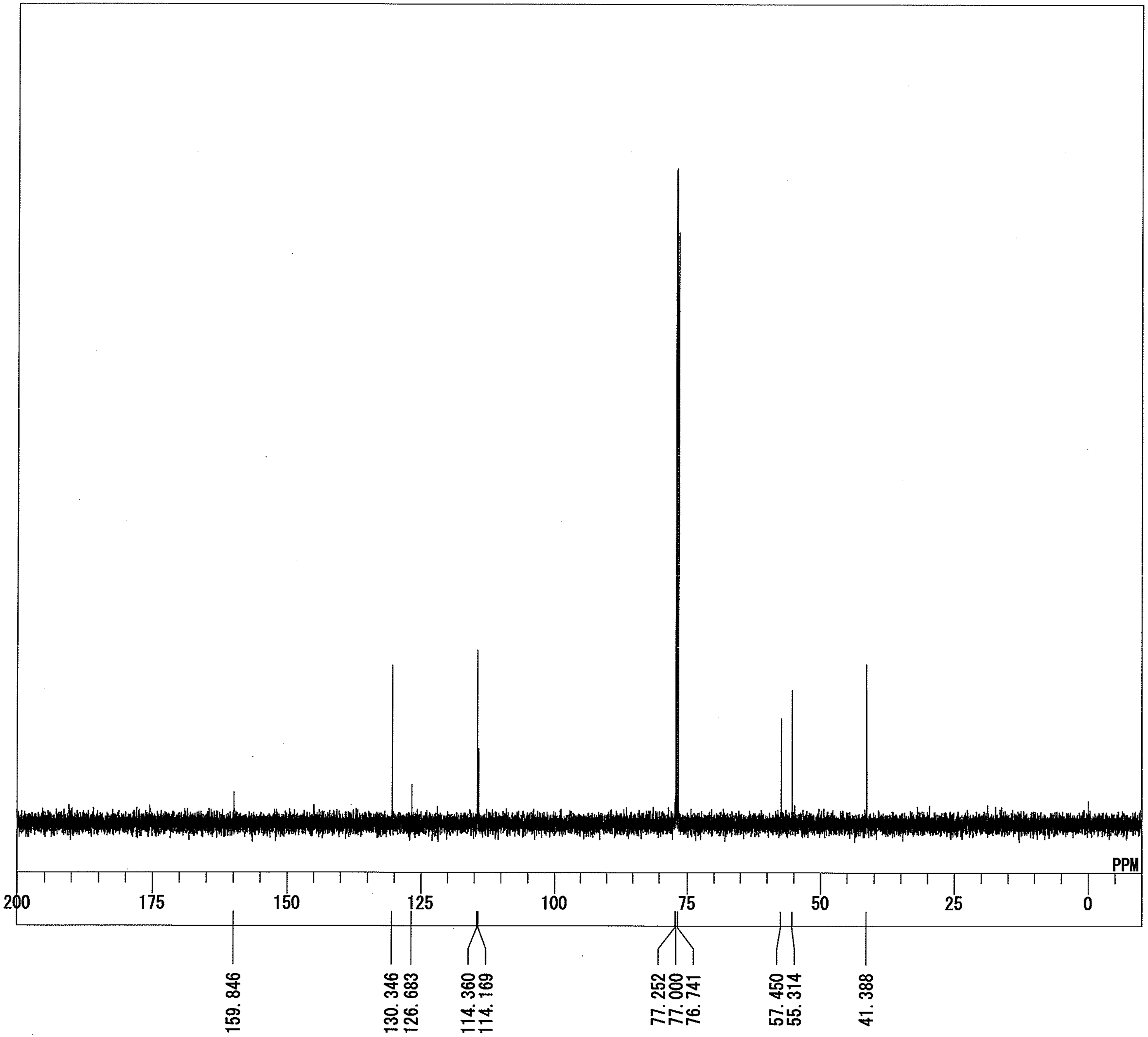

DFILE DI-58-t-1-13C.als
COMNT Single Pulse with Broadband Decoupling
DATIM 25-10-2008 17:31:38
OBNUC 13C
EXMOD single_pulse_dec
OBFRQ 125.77 MHz
OBSET 7.87 KHz
OBFIN 4.21 Hz
POINT 32768
FREQU 31446.54 Hz
SCANS 352
ACQTM 1.0420 sec
PD 1.0000 sec
PW1 4.17 usec
IRNUC 1H
CTEMP 27.3 c
SLVNT CDCL3
EXREF 77.00 ppm
BF 0.23 Hz
RGAIN 30

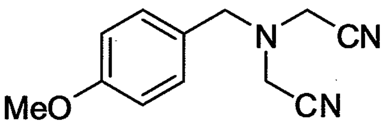

2a

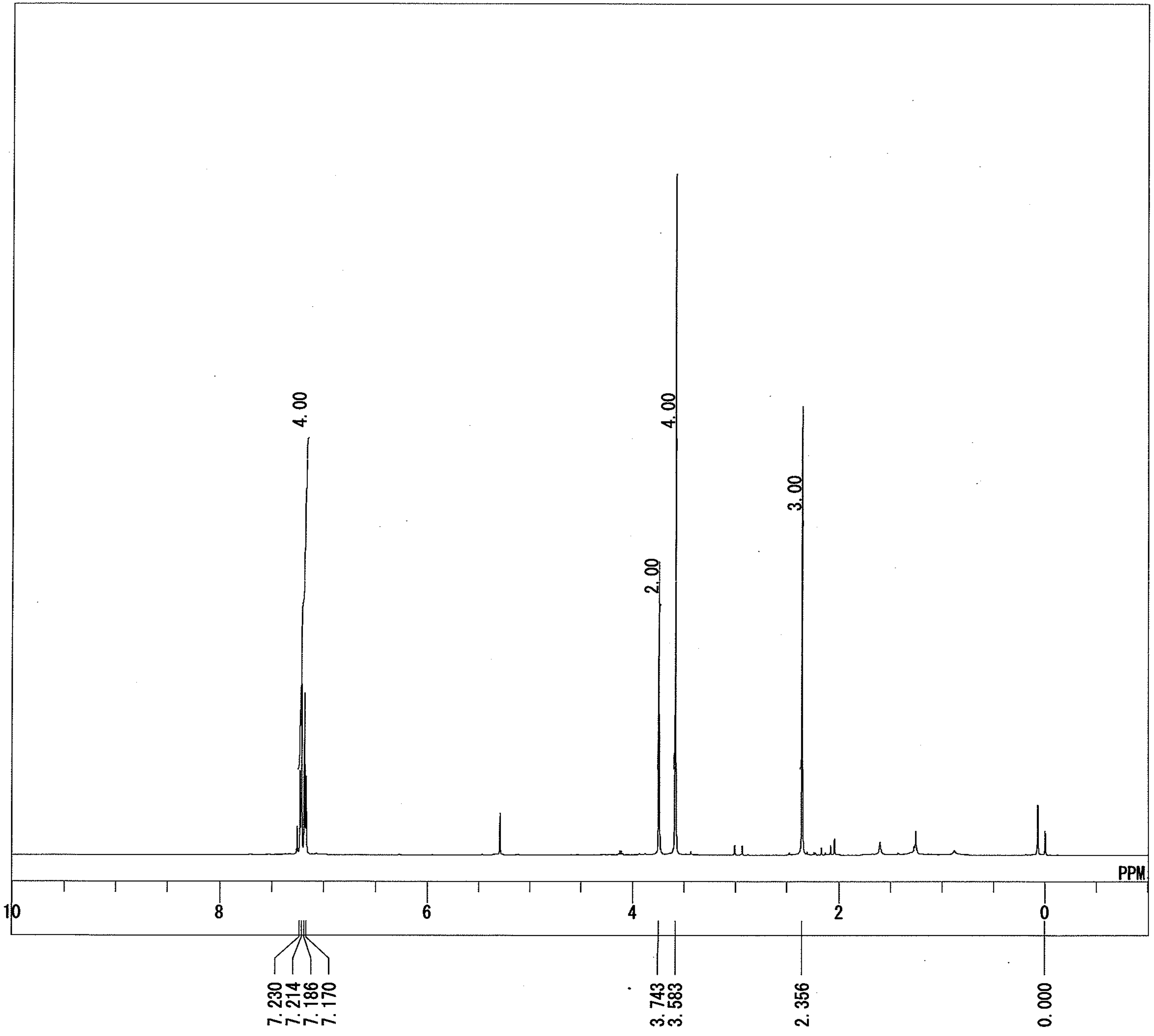

DFILE	DI-60-t-1-1H.als
COMNT	Single Pulse Experiment
DATIM	28-10-2008 12:56:43
OBNUC	1H
EXMOD	single_pulse.exp
OBFRQ	500.16 MHz
OBSET	2.41 KHz
OBFIN	6.01 Hz
POINT	32768
FREQU	7507.51 Hz
SCANS	8
ACQTM	2.1823 sec
PD	4.0000 sec
PW1	7.00 usec
IRNUC	
CTEMP	24.9 c
SLVNT	CDCL3
EXREF	0.00 ppm
BF	0.23 Hz
RGAIN	15

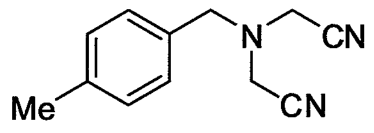

2b

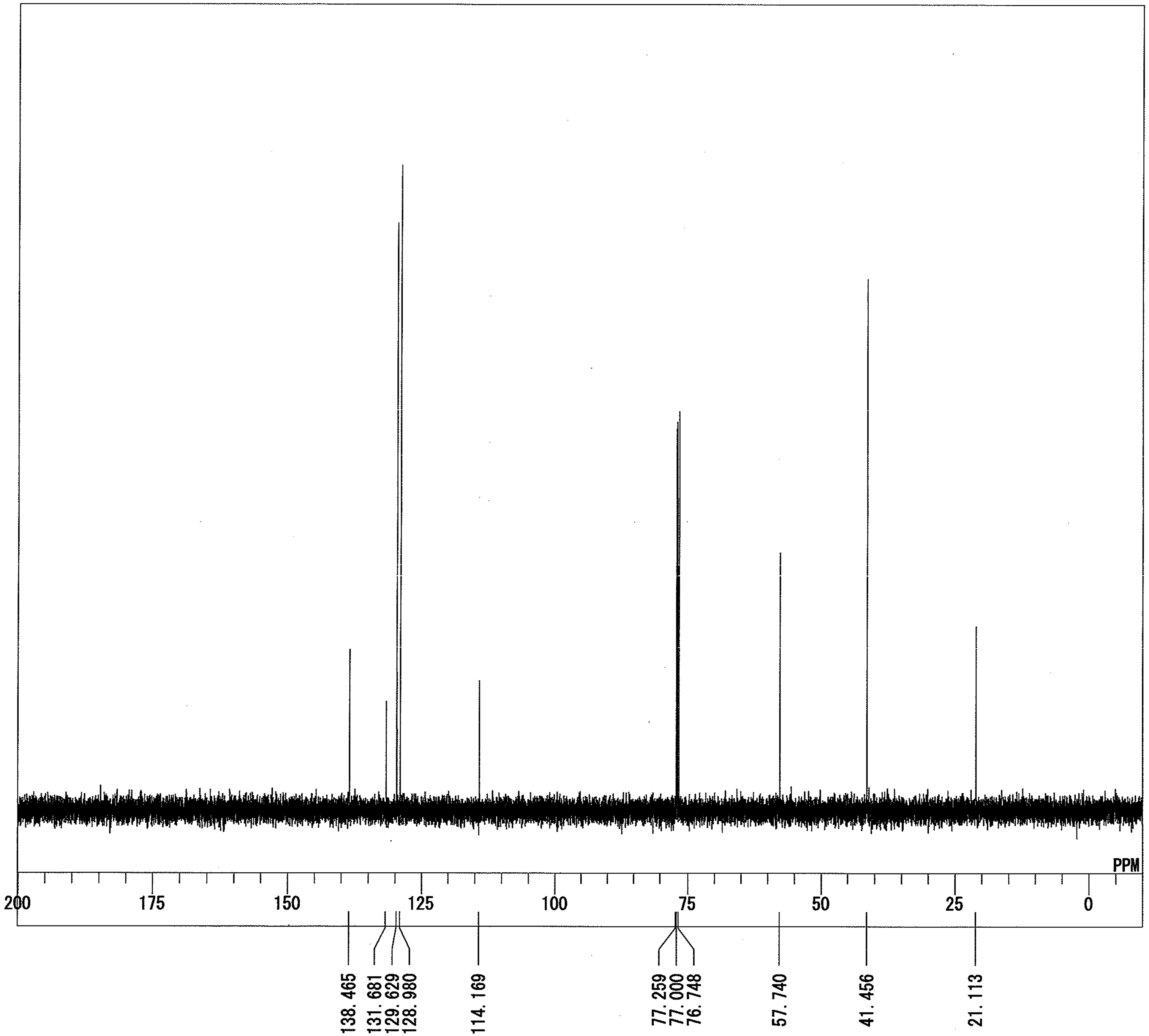

DFILE	DI-60-t-1-13C.als
COMNT	Single Pulse with Broadband Decoupling
DATIM	28-10-2008 13:01:03
OBNUC	13C
EXMOD	single_pulse_dec
OBFRQ	125.77 MHz
OBSET	7.87 KHz
OBFIN	4.21 Hz
POINT	32768
FREQU	31446.54 Hz
SCANS	102
ACQTM	1.0420 sec
PD	1.0000 sec
PW1	4.17 usec
IRNUC	1H
CTEMP	26.7 c
SLVNT	CDCL3
EXREF	77.00 ppm
BF	0.23 Hz
RGAIN	30

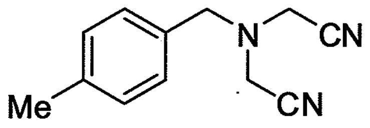

2b

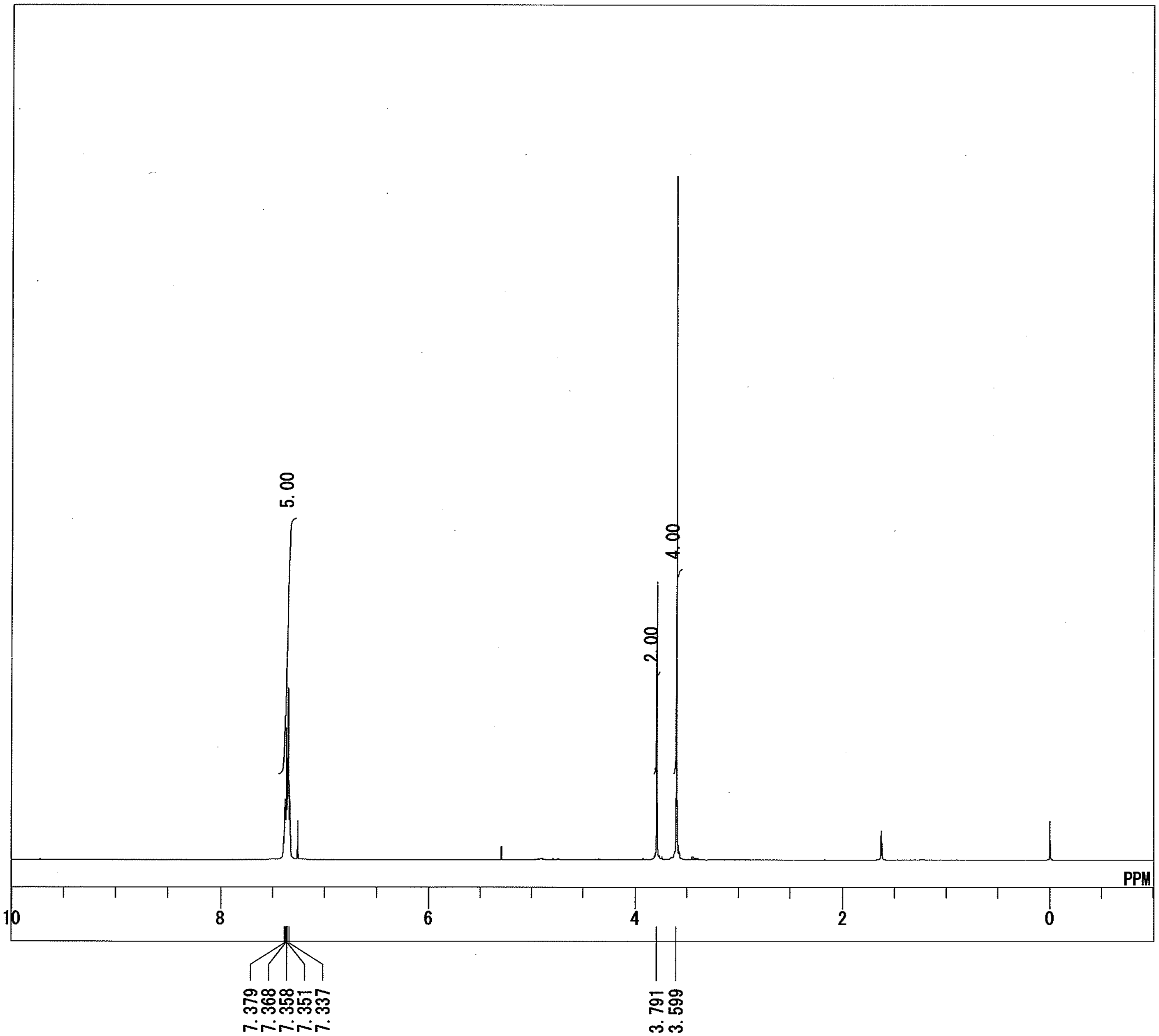

DFILE	NT-5CR-BnNH2-1H.als
COMNT	Single Pulse Experiment
DATIM	04-09-2012 00:18:35
OBNUC	1H
EXMOD	single_pulse.exp
OBFRQ	500.16 MHz
OBSET	2.41 KHz
OBFIN	6.01 Hz
POINT	32768
FREQU	7507.51 Hz
SCANS	8
ACQTM	2.1823 sec
PD	4.0000 sec
PW1	7.00 usec
IRNUC	
CTEMP	22.3 c
SLVNT	CDCL3
EXREF	0.00 ppm
BF	0.23 Hz
RGAIN	14

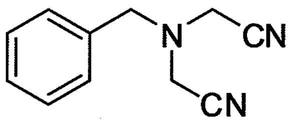

2c

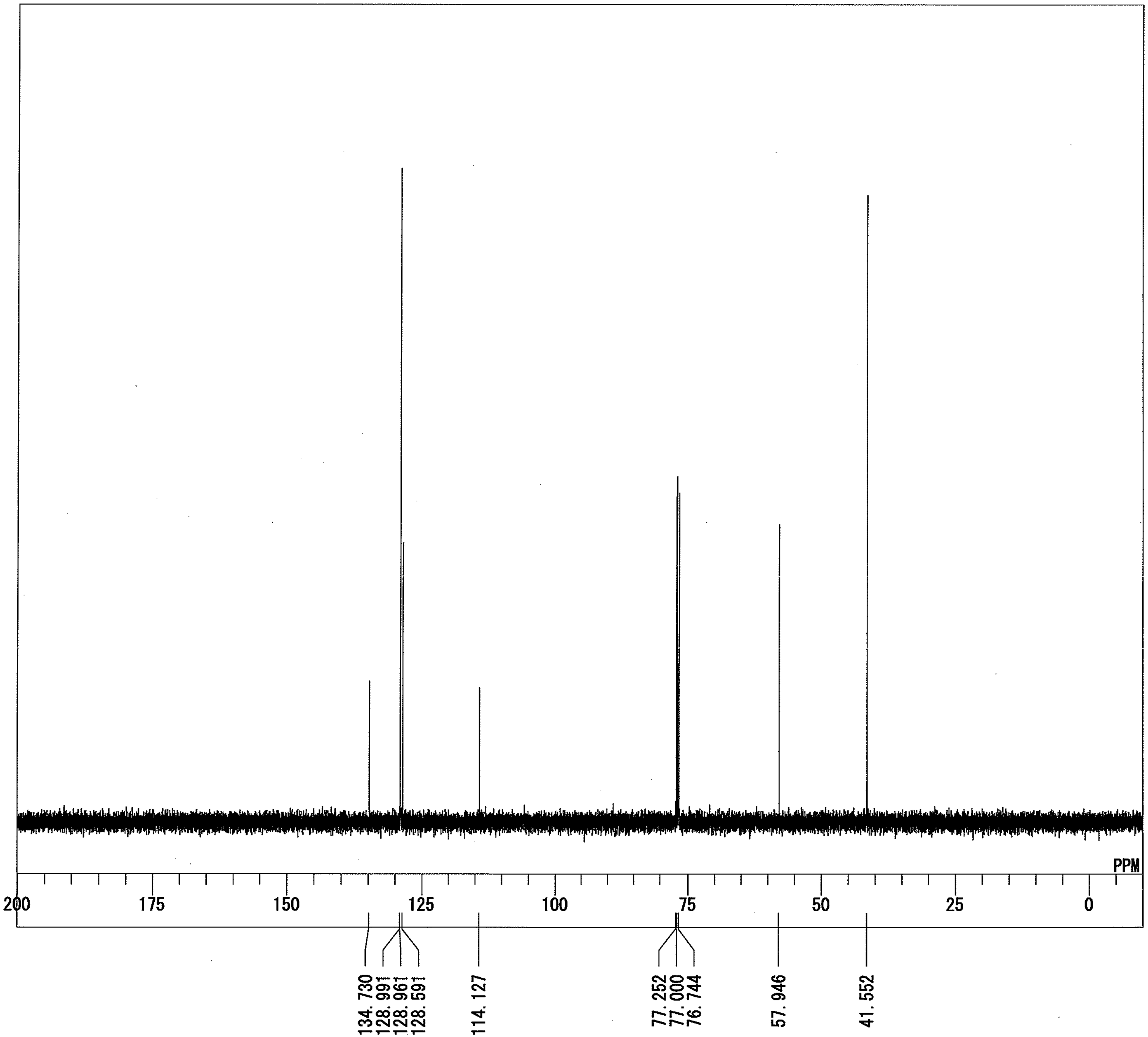

DFILE	NT-5CR-BnNH2-13C.als
COMNT	Single Pulse with Broadband Decoupling
DATIM	04-09-2012 00:27:04
OBNUC	13C
EXMOD	single_pulse_dec
OBFRQ	125.77 MHz
OBSET	7.87 KHz
OBFIN	4.21 Hz
POINT	65536
FREQU	31446.54 Hz
SCANS	139
ACQTM	1.0420 sec
PD	1.0000 sec
PW1	4.17 usec
IRNUC	1H
CTEMP	24.2 c
SLVNT	CDCL3
EXREF	77.00 ppm
BF	0.23 Hz
RGAIN	30

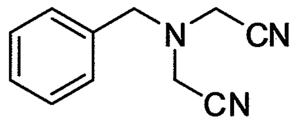

2c

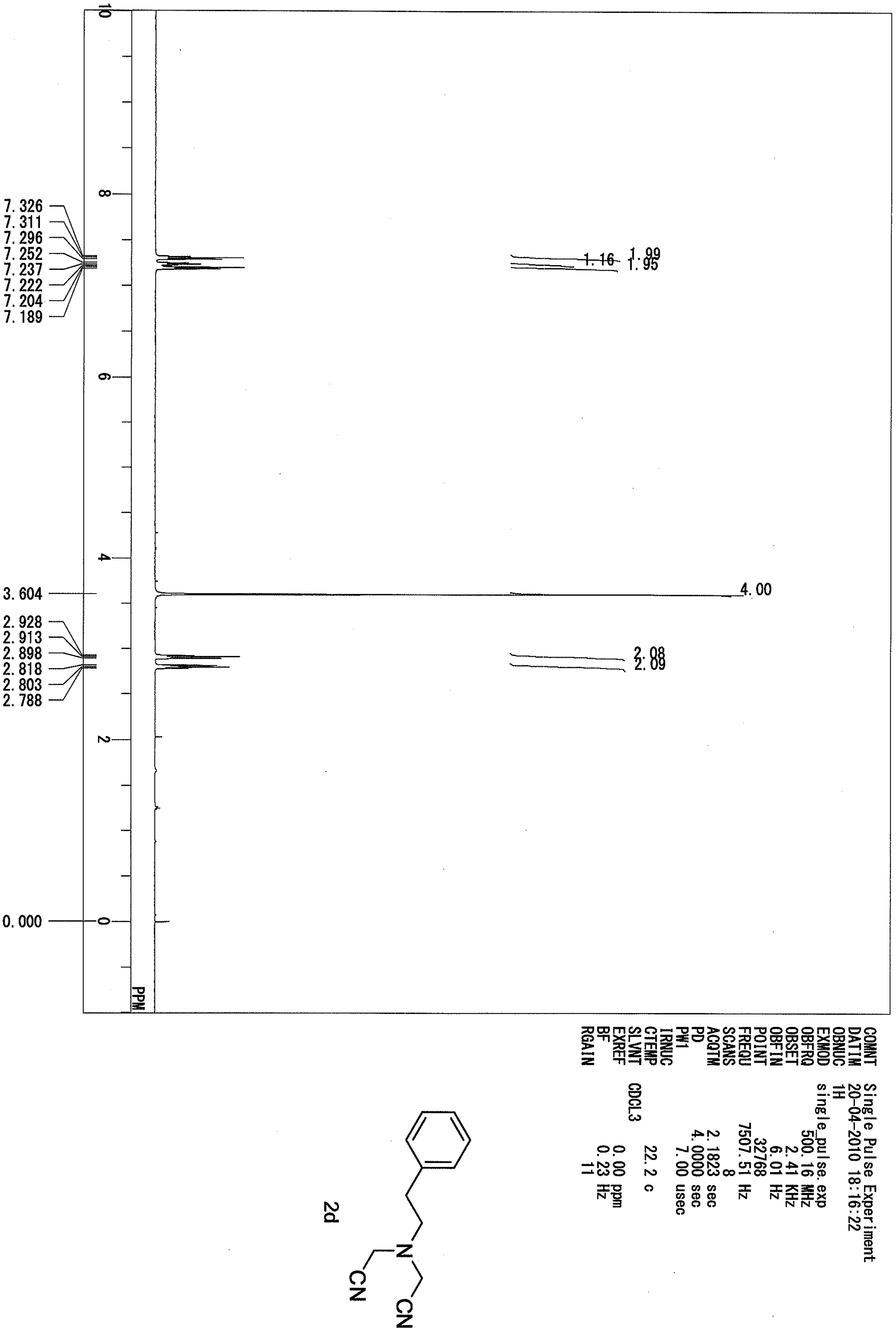
7.326
7.311
7.296
7.252
7.237
7.222
7.204
7.189
3.604
2.928
2.913
2.898
2.818
2.803
2.788
0.000
PPM
1.16
1.99
1.95
4.00
2.08
2.09
COMNT Single Pulse Experiment
DATIM 20-04-2010 18:16:22
OBNUC 1H
EXMOD single_pulse.exp
OBFRQ 500.16 MHz
OBSET 2.41 KHz
OBFIN 6.01 Hz
POINT 32768
FREQU 7507.51 Hz
SCANS 8
ACQTM 2.1823 sec
PD 4.0000 sec
PW1 7.00 usec
IRNUC
CTEMP 22.2 c
SLVNT CDCL3
EXREF 0.00 ppm
BF 0.23 Hz
RGAIN 11
N
CN
CN

2d

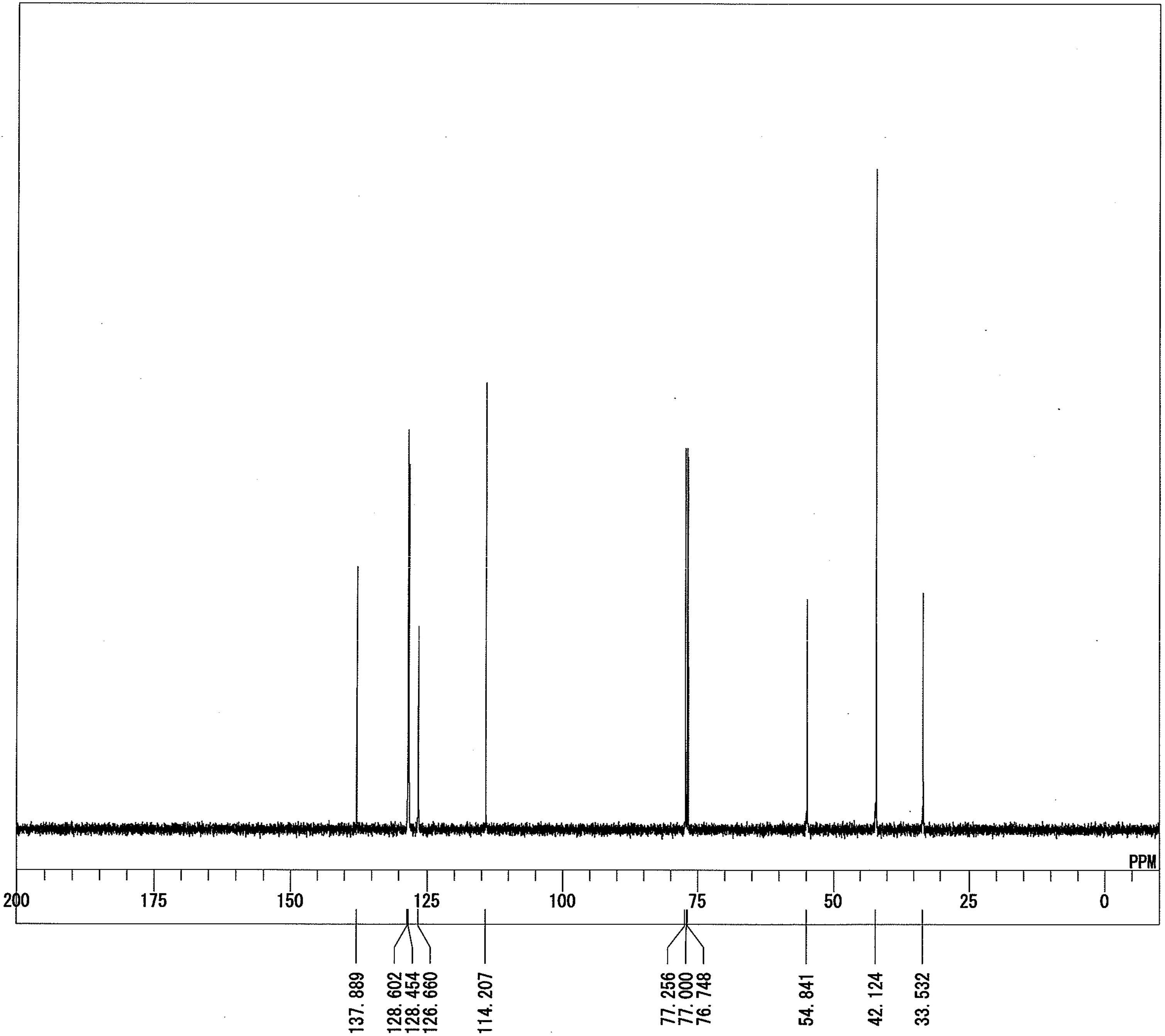


```
COMNT   Single Pulse with Broadband Decoupling
DATIM   20-04-2010 18:27:28
OBNUC   13C
EXMOD   single_pulse_dec
OBFRQ          125.77 MHz
OBSET            7.87 KHz
OBFIN            4.21 Hz
POINT           65536
FREQU        31446.54 Hz
SCANS             249
ACQTM          1.0420 sec
PD             1.0000 sec
PW1              4.17 usec
IRNUC   1H
CTEMP            23.5 c
SLVNT   CDCL3
EXREF           77.00 ppm
BF               1.00 Hz
RGAIN              30
```

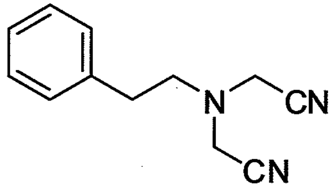

2d

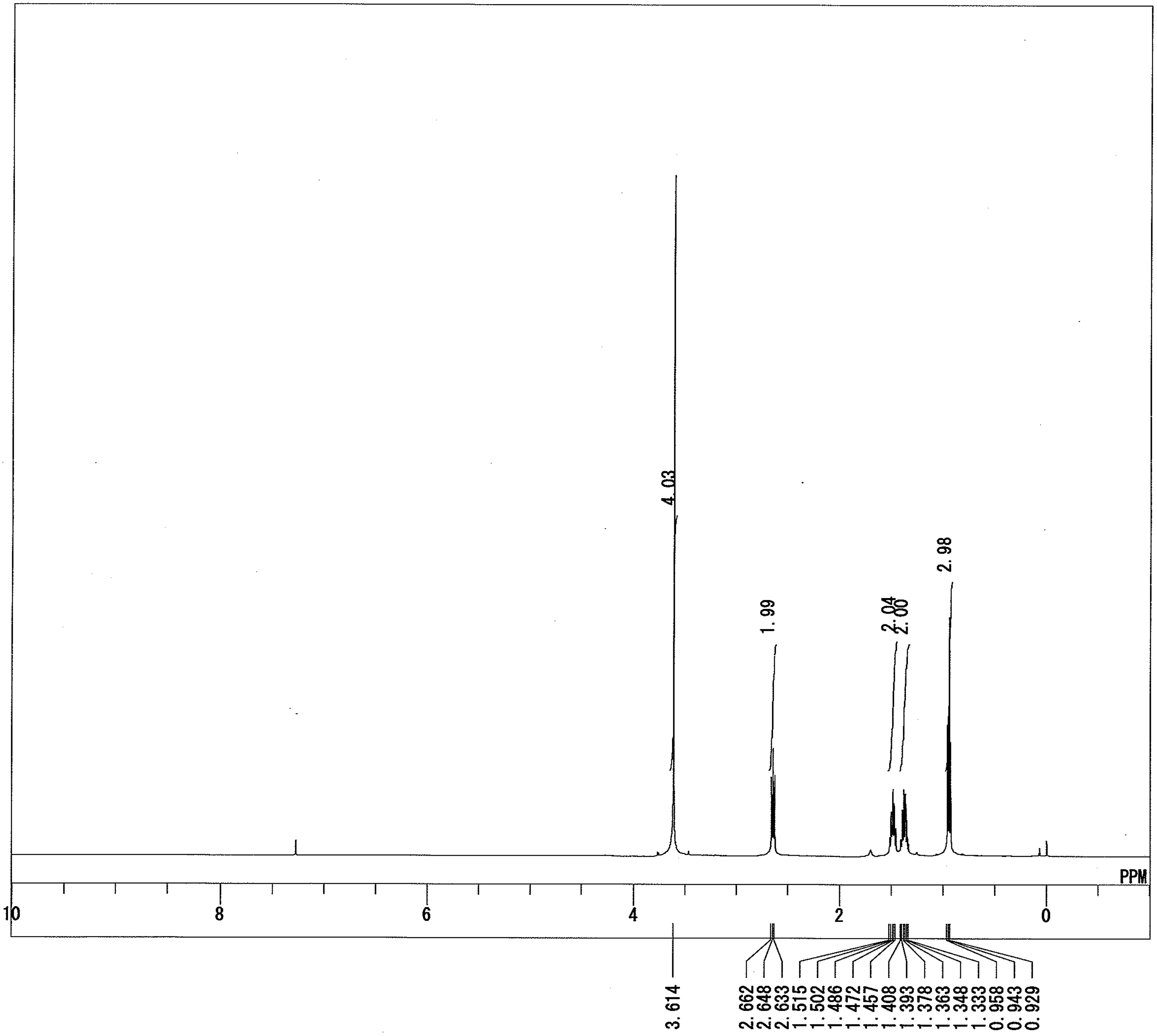

DFILE	NT-5CR-nBuNH2-1H-2.als
COMNT	Single Pulse Experiment
DATIM	05-09-2012 01:59:24
OBNUC	1H
EXMOD	single_pulse.exp
OBFRQ	500.16 MHz
OBSET	2.41 KHz
OBFIN	6.01 Hz
POINT	32768
FREQU	7507.51 Hz
SCANS	8
ACQTM	2.1823 sec
PD	4.0000 sec
PW1	7.00 usec
IRNUC	
CTEMP	22.8 c
SLVNT	CDCL3
EXREF	0.00 ppm
BF	0.23 Hz
RGAIN	12

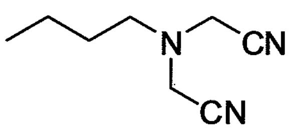

2e

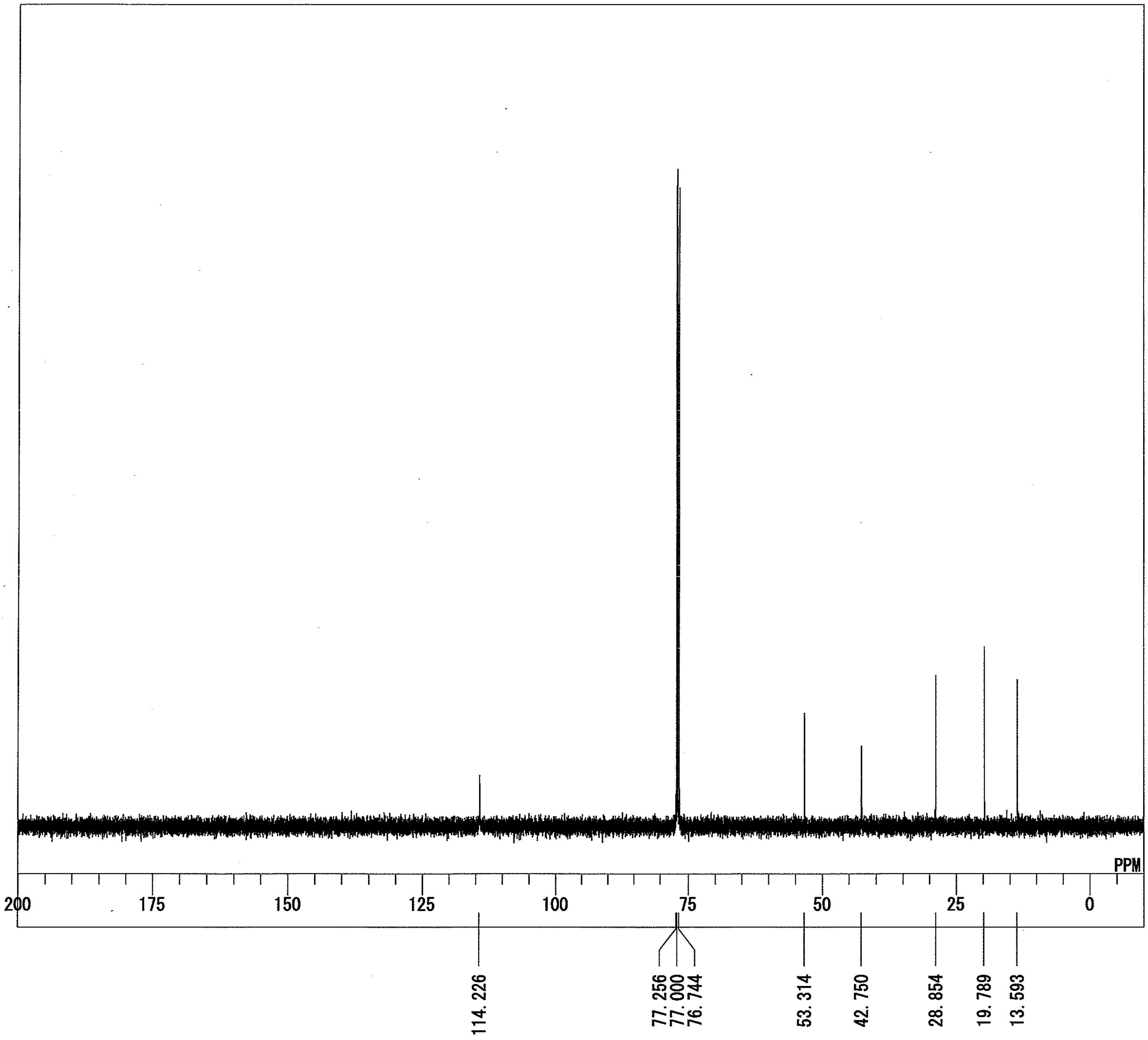

DFILE	NT-5CR-nBuNH2-13C.als
COMNT	Single Pulse with Broadband Decoupling
DATIM	04-09-2012 02:12:43
OBNUC	13C
EXMOD	single_pulse_dec
OBFRQ	125.77 MHz
OBSET	7.87 KHz
OBFIN	4.21 Hz
POINT	65536
FREQU	31446.54 Hz
SCANS	147
ACQTM	1.0420 sec
PD	1.0000 sec
PW1	4.17 usec
IRNUC	1H
CTEMP	24.1 c
SLVNT	CDCL3
EXREF	77.00 ppm
BF	0.23 Hz
RGAIN	30

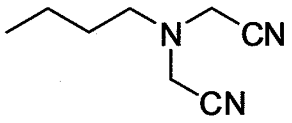

2e

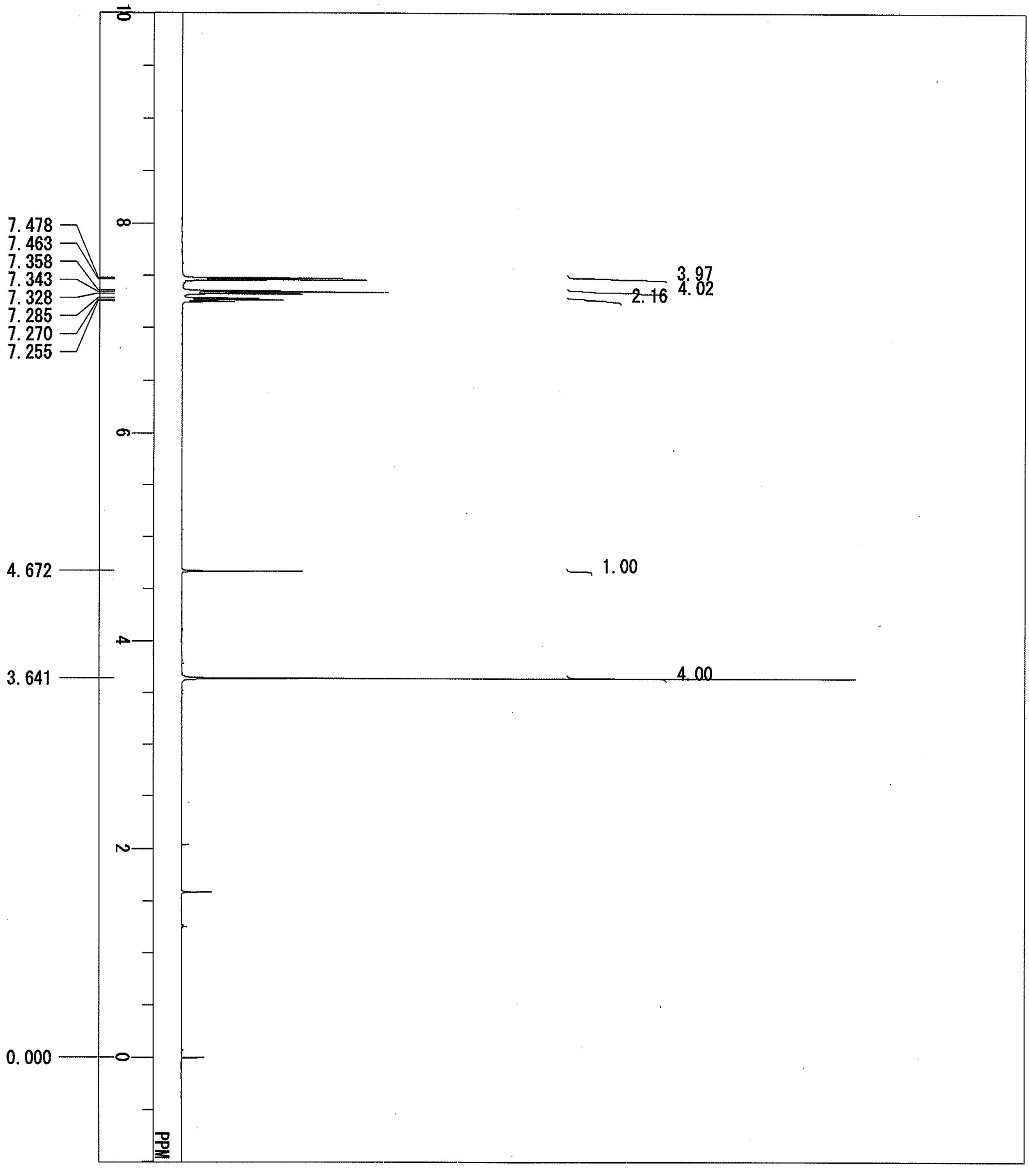
7.478
7.463
7.358
7.343
7.328
7.285
7.270
7.255
4.672
3.641
0.000
3.97
4.02
2.16
1.00
4.00
10
8
6
4
2
0
PPM
COMNT Single Pulse Experiment
DATIM 18-05-2010 14:27:60
OBNUC 1H
EXMOD single_pulse.exp
OBFRQ 500.16 MHz
OBSET 2.41 KHz
OBFIN 6.01 Hz
POINT 32768
FREQU 7507.51 Hz
SCANS 8
ACQTM 2.1823 sec
PD 4.0000 sec
PW1 7.00 usec
IRNUC
CTEMP 22.2 c
SLVNT CDCL3
EXREF 0.00 ppm
BF 0.23 Hz
RGAIN 14

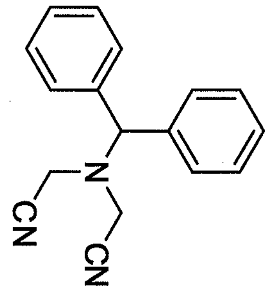
N
CN
CN

2f

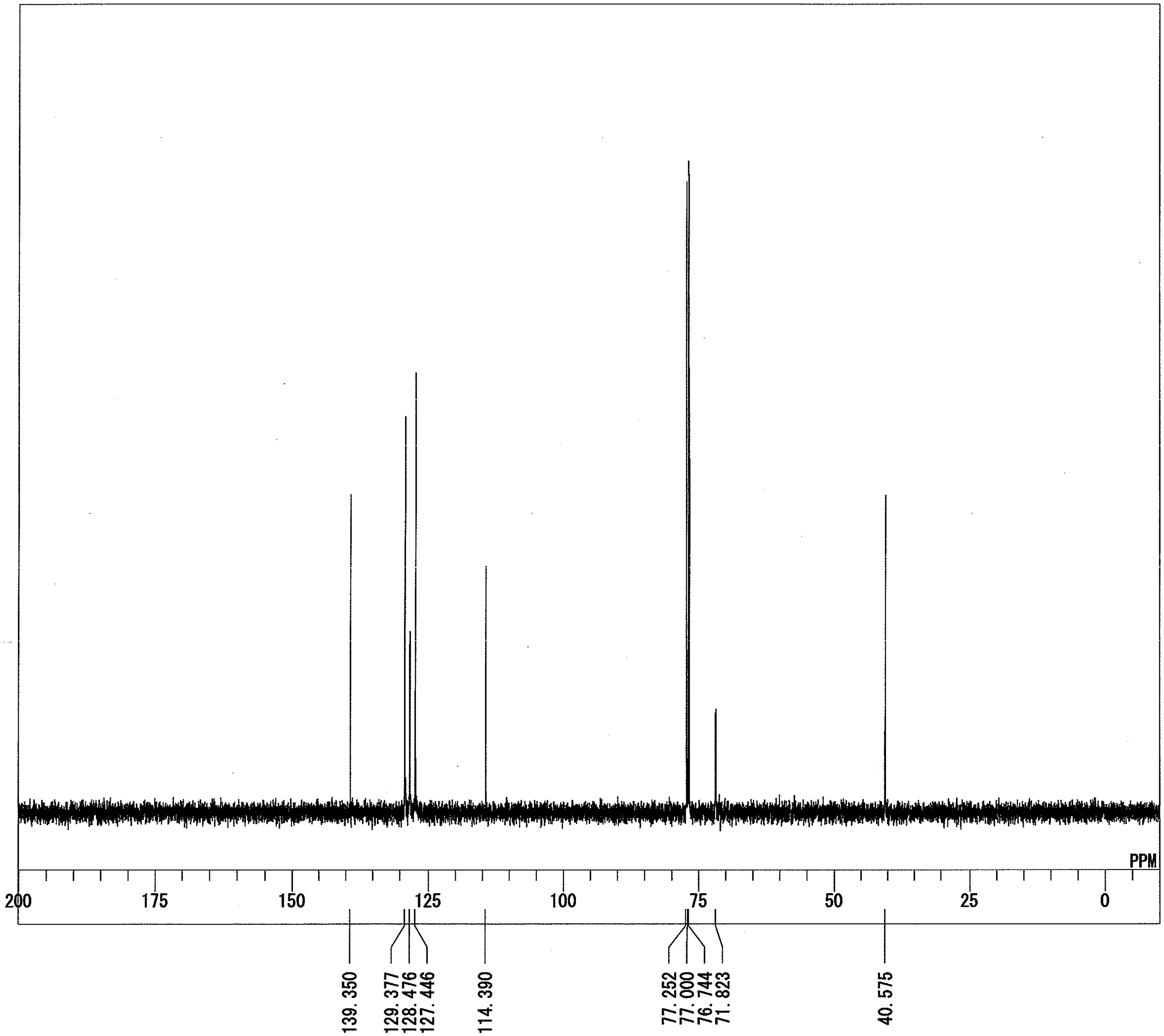

COMNT Single Pulse with Broadband Decoupling
DATIM 18-05-2010 14:36:51
OBNUC 13C
EXMOD single_pulse_dec
OBFRQ 125.77 MHz
OBSET 7.87 KHz
OBFIN 4.21 Hz
POINT 65536
FREQU 31446.54 Hz
SCANS 174
ACQTM 1.0420 sec
PD 1.0000 sec
PW1 4.17 usec
IRNUC 1H
CTEMP 23.5 c
SLVNT CDCL3
EXREF 77.00 ppm
BF 1.00 Hz
RGAIN 30

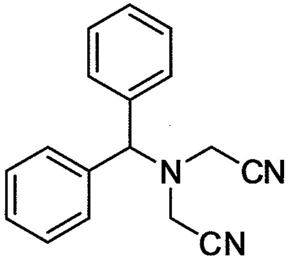

2f

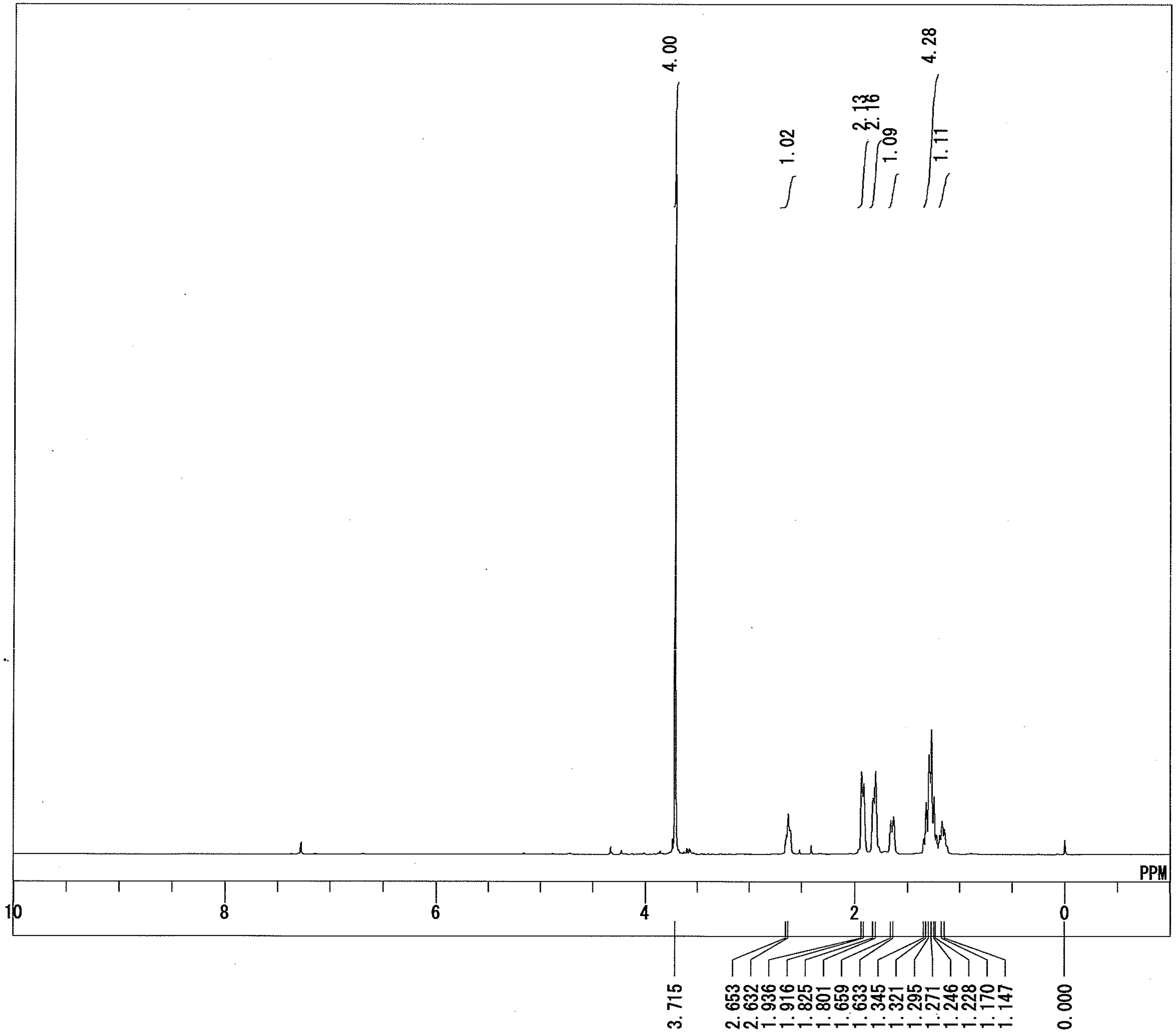

COMNT	Single Pulse Experiment
DATIM	24-04-2010 13:07:39
OBNUC	1H
EXMOD	single_pulse.exp
OBFRQ	500.16 MHz
OBSET	2.41 KHz
OBFIN	6.01 Hz
POINT	32768
FREQU	7507.51 Hz
SCANS	8
ACQTM	2.1823 sec
PD	4.0000 sec
PW1	7.00 usec
IRNUC	
CTEMP	22.1 c
SLVNT	CDCL3
EXREF	0.00 ppm
BF	0.23 Hz
RGAIN	11

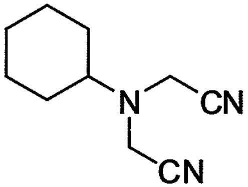

2g

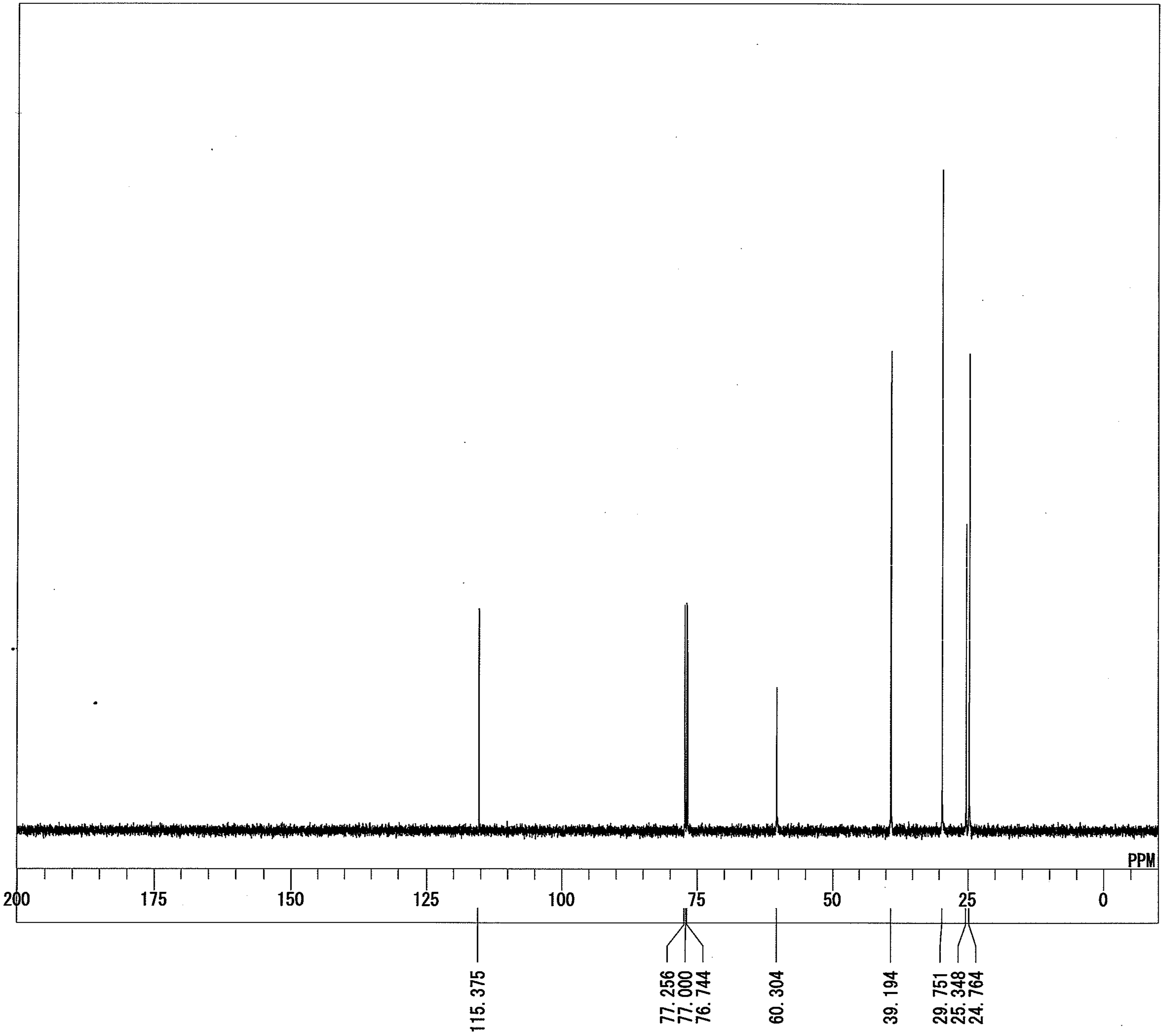

COMNT Single Pulse with Broadband Decoupling
DATIM 24-04-2010 13:17:27
OBNUC 13C
EXMOD single_pulse_dec
OBFRQ 125.77 MHz
OBSET 7.87 KHz
OBFIN 4.21 Hz
POINT 65536
FREQU 31446.54 Hz
SCANS 206
ACQTM 1.0420 sec
PD 1.0000 sec
PW1 4.17 usec
IRNUC 1H
CTEMP 23.4 c
SLVNT CDCL3
EXREF 77.00 ppm
BF 1.00 Hz
RGAIN 30

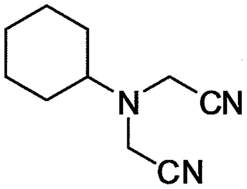

2g

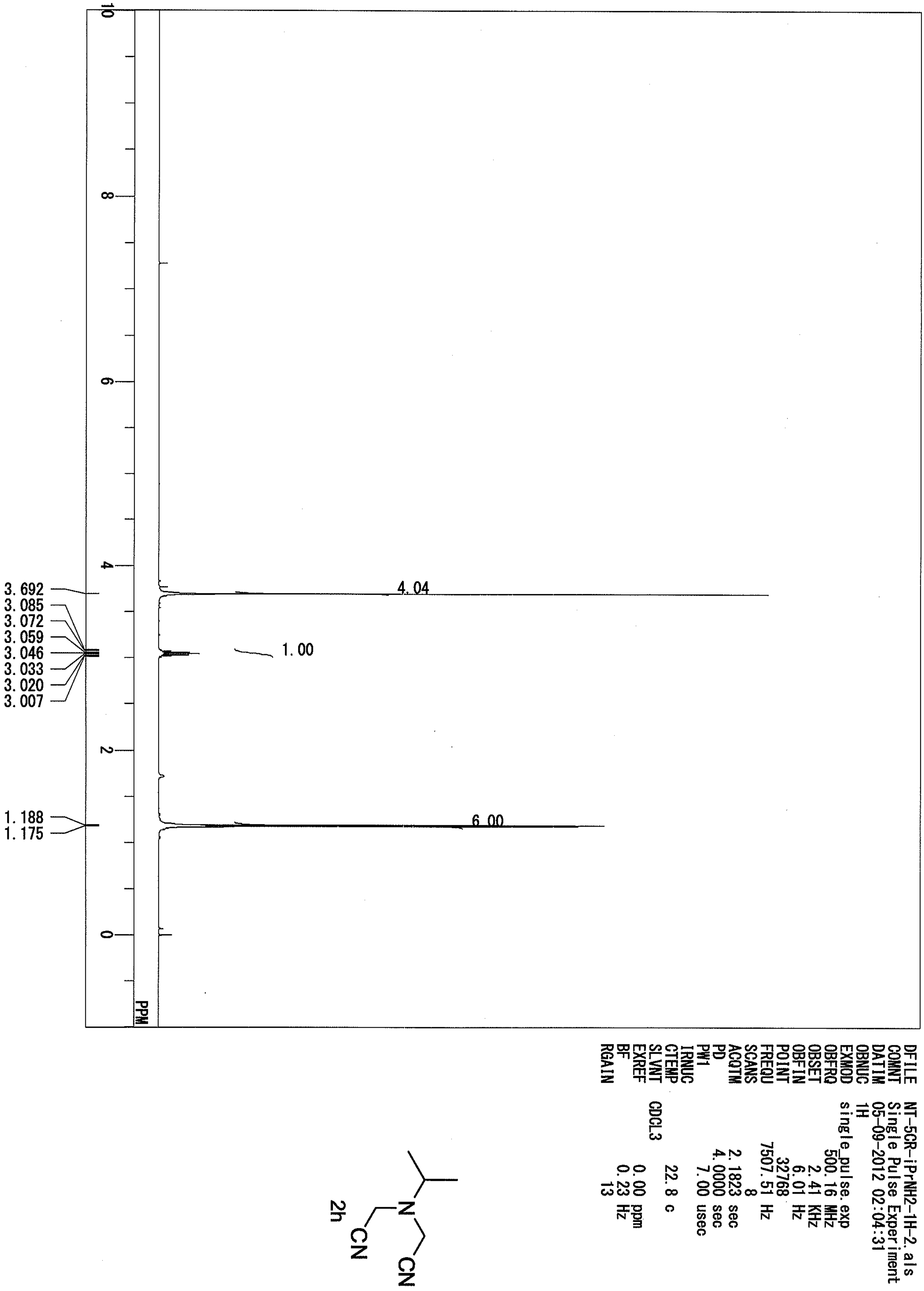

DFILE NT-5CR-iPrNH2-1H-2.als
COMNT Single Pulse Experiment
DATIM 05-09-2012 02:04:31
OBNUC 1H
EXMOD single_pulse.exp
OBFRQ 500.16 MHz
OBSET 2.41 KHz
OBFIN 6.01 Hz
POINT 32768
FREQU 7507.51 Hz
SCANS 8
ACQTM 2.1823 sec
PD 4.0000 sec
PW1 7.00 usec
IRNUC
CTEMP 22.8 c
SLVNT CDCL3
EXREF 0.00 ppm
BF 0.23 Hz
RGAIN 13
3.692
3.085
3.072
3.059
3.046
3.033
3.020
3.007
1.188
1.175
4.04
1.00
6.00
PPM
N
CN
CN
2h

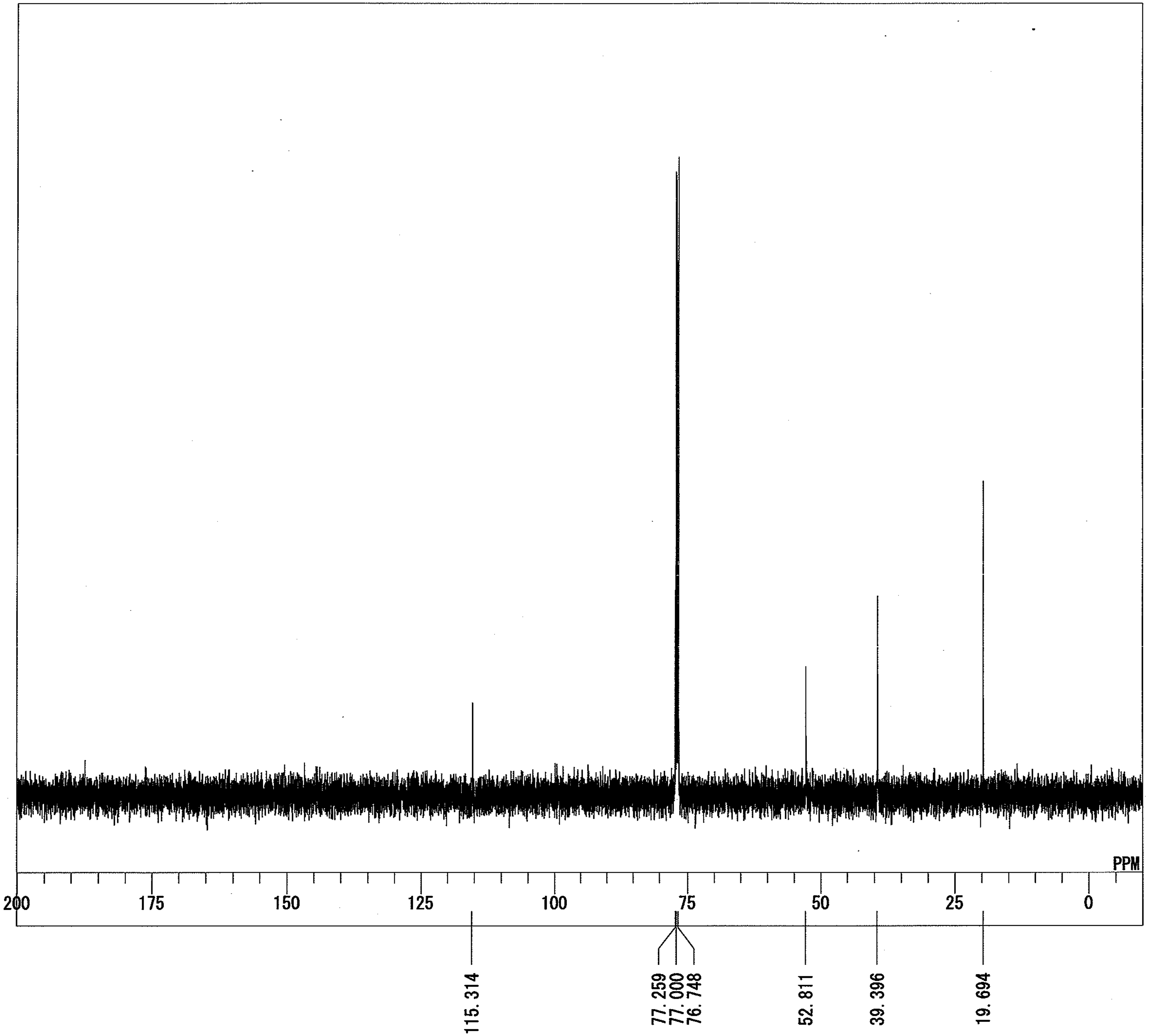

DFILE	NT-5CR-iPrNH2-13C.als
COMNT	Single Pulse with Broadband Decoupling
DATIM	04-09-2012 02:26:38
OBNUC	13C
EXMOD	single_pulse_dec
OBFRQ	125.77 MHz
OBSET	7.87 KHz
OBFIN	4.21 Hz
POINT	32768
FREQU	31446.54 Hz
SCANS	202
ACQTM	1.0420 sec
PD	1.0000 sec
PW1	4.17 usec
IRNUC	1H
CTEMP	24.4 c
SLVNT	CDCL3
EXREF	77.00 ppm
BF	0.23 Hz
RGAIN	30

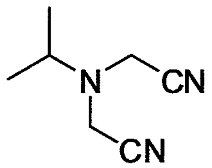

2h

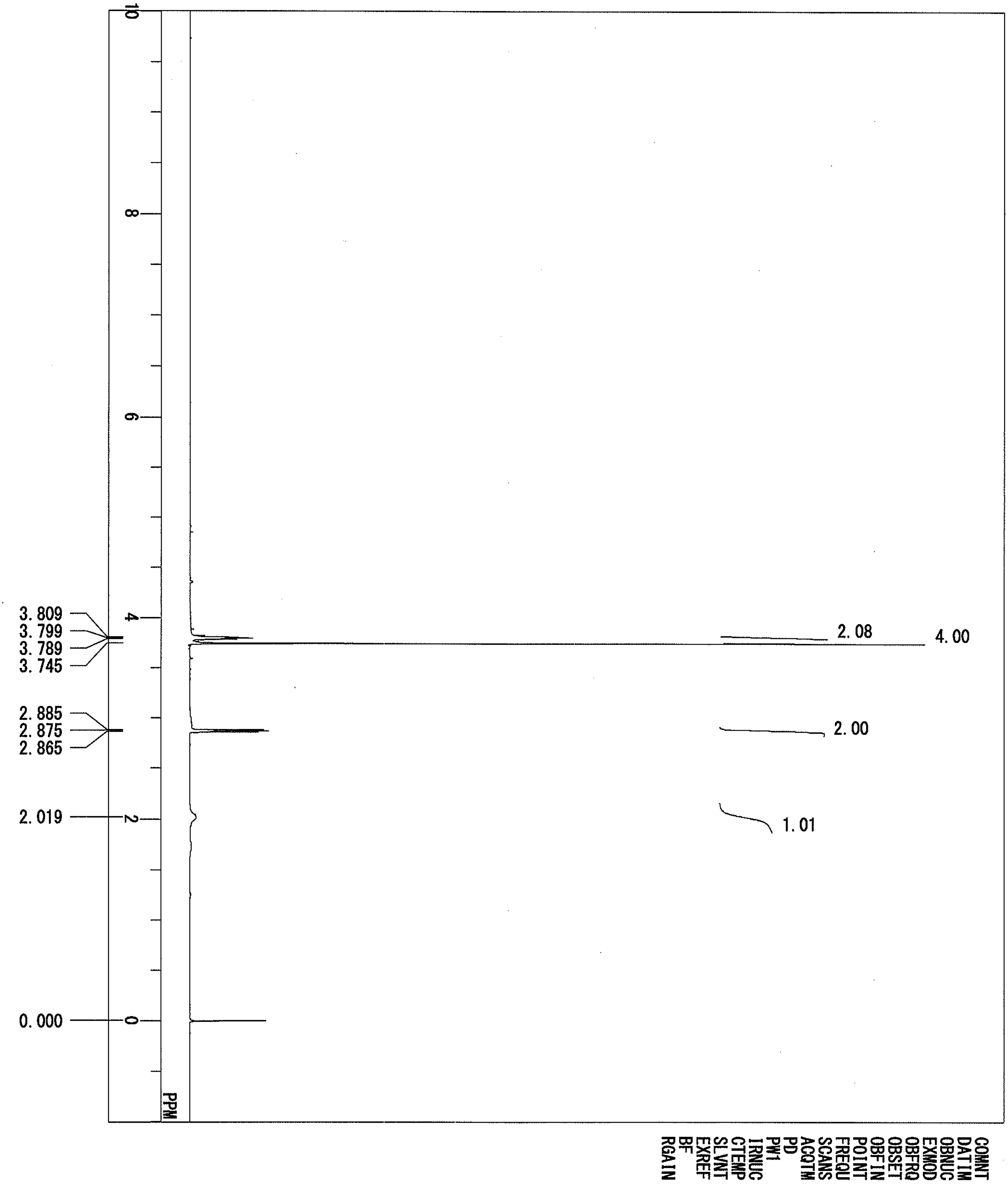
3.809
3.799
3.789
3.745
2.885
2.875
2.865
2.019
0.000
PPM
2.08
4.00
2.00
1.01
COMNT Single Pulse Experiment
DATIM 14-12-2009 14:39:51
OBNUC 1H
EXMOD single_pulse.exp
OBFRQ 500.16 MHz
OBSET 2.41 KHz
OBFIN 6.01 Hz
POINT 32768
FREQU 7507.51 Hz
SCANS 8
ACQTM 2.1823 sec
PD 4.0000 sec
PW1 7.00 usec
IRNUC
CTEMP 22.1 c
SLVNT CDCL3
EXREF 0.00 ppm
BF 0.23 Hz
RGAIN 17

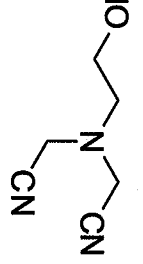
HO
N
CN
CN

2i

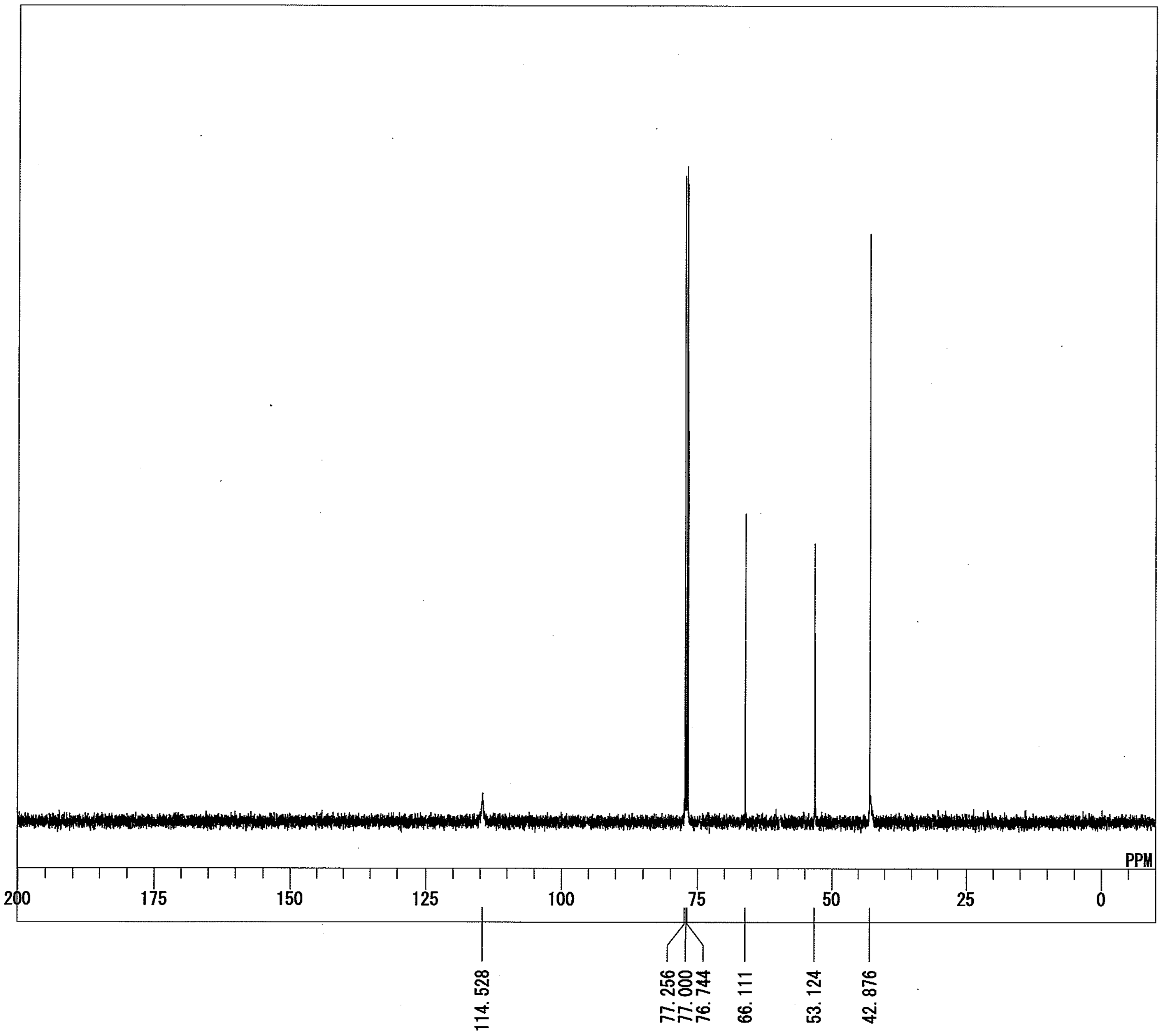

COMNT	Single Pulse with Broadband Decoupling
DATIM	10-03-2011 16:26:05
OBNUC	13C
EXMOD	single_pulse_dec
OBFRQ	125.77 MHz
OBSET	7.87 KHz
OBFIN	4.21 Hz
POINT	65536
FREQU	31446.54 Hz
SCANS	1007
ACQTM	1.0420 sec
PD	1.0000 sec
PW1	4.17 usec
IRNUC	1H
CTEMP	23.5 c
SLVNT	CDCL3
EXREF	77.00 ppm
BF	1.00 Hz
RGAIN	30

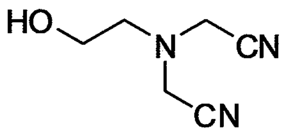

2i

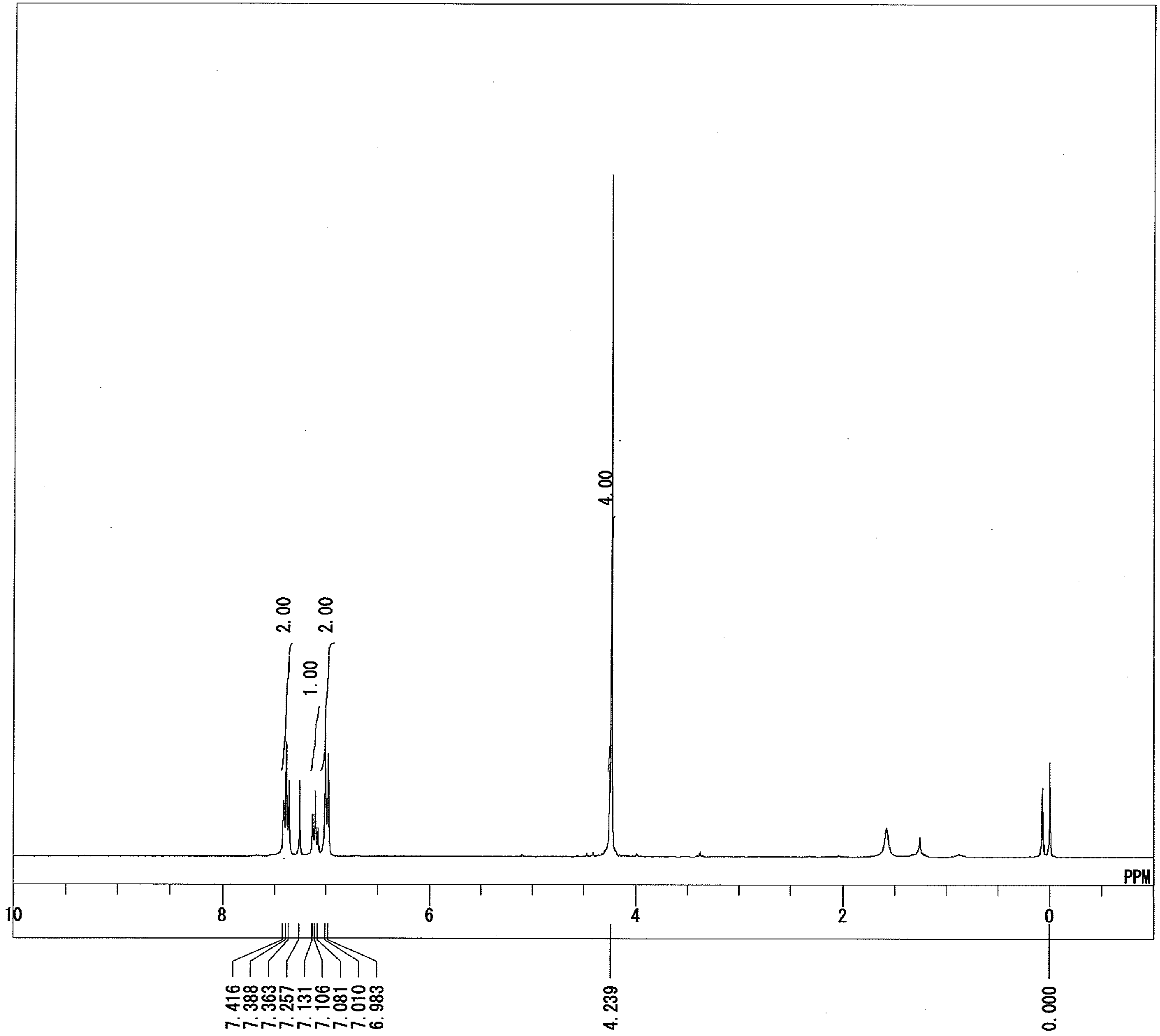

DFILE DI-129-2-E-T1-1H.als
COMNT 090608
DATIM Mon Jun 08 20:56:25 2009
OBNUC 1H
EXMOD NON
OBFRQ 300.40 MHz
OBSET 130.00 KHz
OBFIN 1150.00 Hz
POINT 65536
FREQU 6013.20 Hz
SCANS 8
ACQTM 5.4493 sec
PD 1.5510 sec
PW1 5.50 usec
IRNUC 1H
CTEMP 23.4 c
SLVNT CDCL3
EXREF 0.00 ppm
BF 0.09 Hz
RGAIN 19

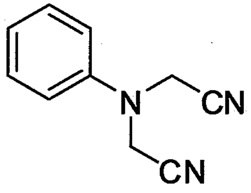

2j

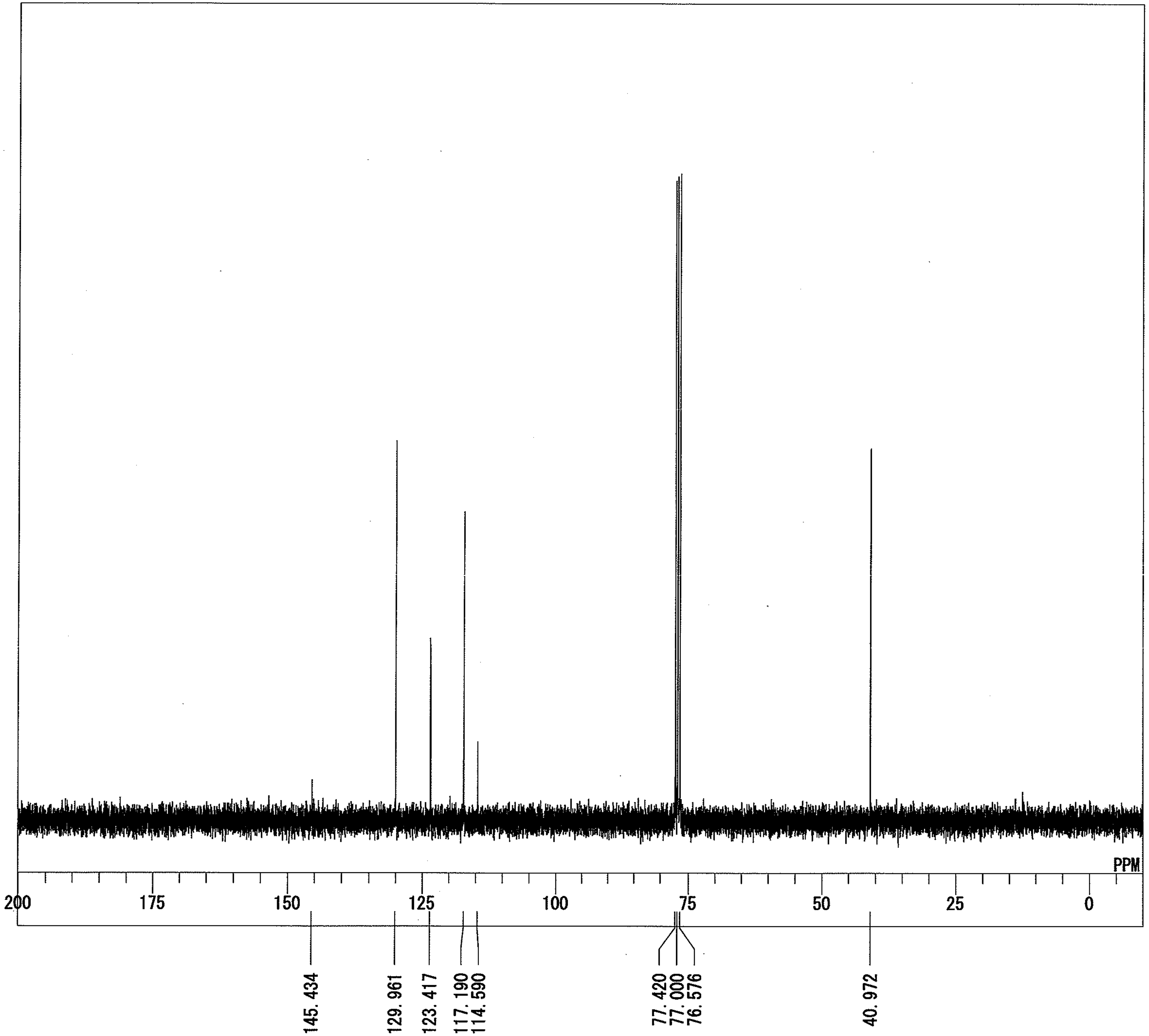

DFILE DI-129-2-E-T1-13C.als
COMNT 090608
DATIM Mon Jun 08 20:53:29 2009
OBNUC 13C
EXMOD BCM
OBFRQ 75.45 MHz
OBSET 124.00 KHz
OBFIN 1840.00 Hz
POINT 65536
FREQU 20408.10 Hz
SCANS 311
ACQTM 1.6056 sec
PD 1.3940 sec
PW1 4.00 usec
IRNUC 1H
CTEMP 23.9 c
SLVNT CDCL3
EXREF 77.00 ppm
BF 0.31 Hz
RGAIN 26
PPM
200
175
150
125
100
75
50
25
0
145.434
129.961
123.417
117.190
114.590
77.420
77.000
76.576
40.972

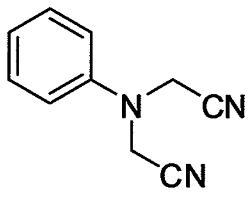

N
CN
CN

2j

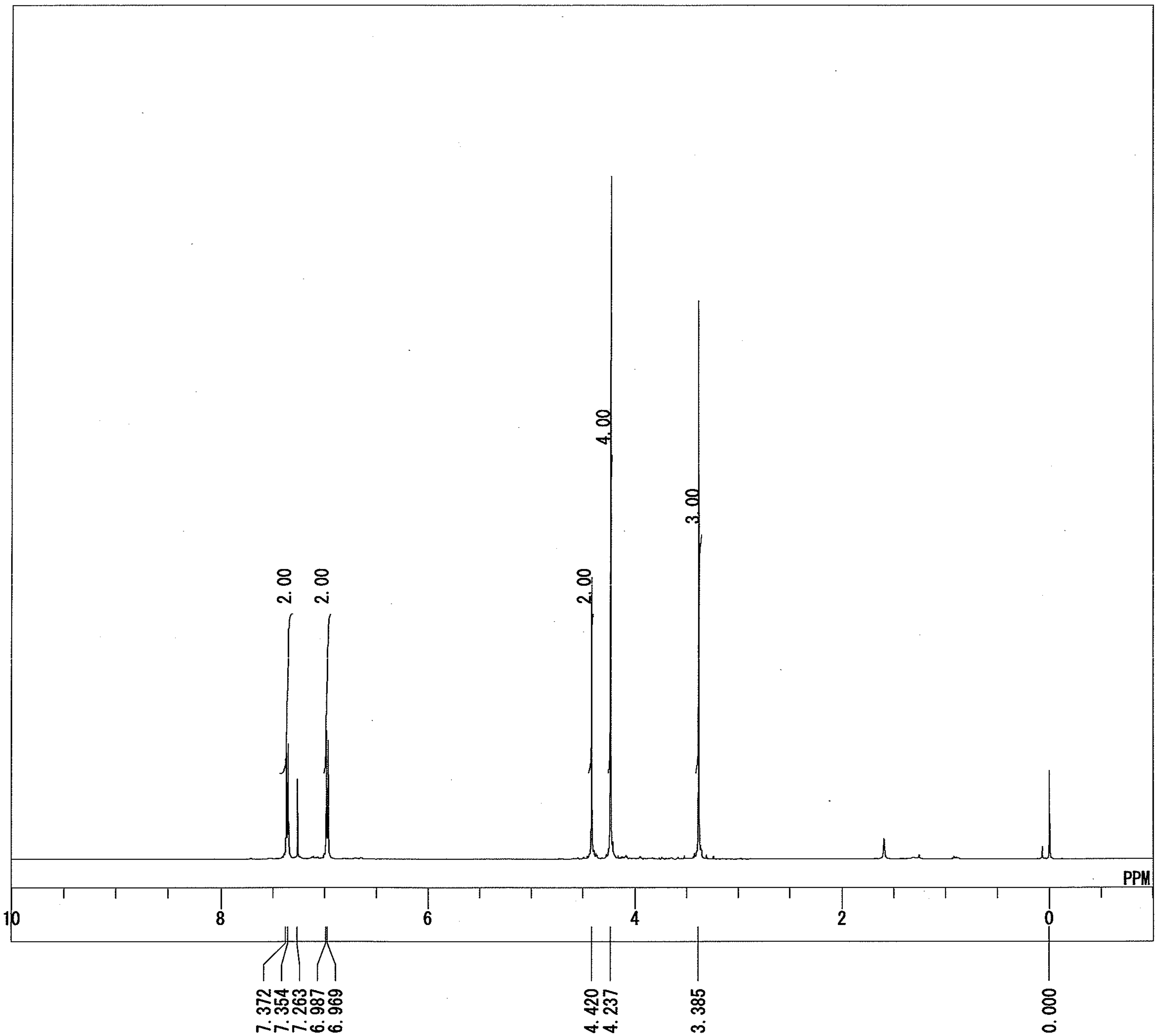

DFILE	NT-195-5-i-1H.als
COMNT	Single Pulse Experiment
DATIM	08-02-2012 22:32:21
OBNUC	1H
EXMOD	single_pulse.exp
OBFRQ	500.16 MHz
OBSET	2.41 KHz
OBFIN	6.01 Hz
POINT	32768
FREQU	7507.51 Hz
SCANS	8
ACQTM	2.1823 sec
PD	4.0000 sec
PW1	7.00 usec
IRNUC	
CTEMP	19.1 c
SLVNT	CDCL3
EXREF	0.00 ppm
BF	1.00 Hz
RGAIN	18

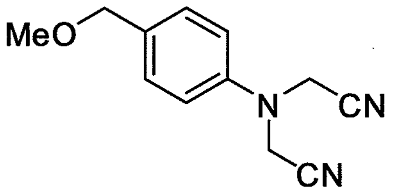

2j'

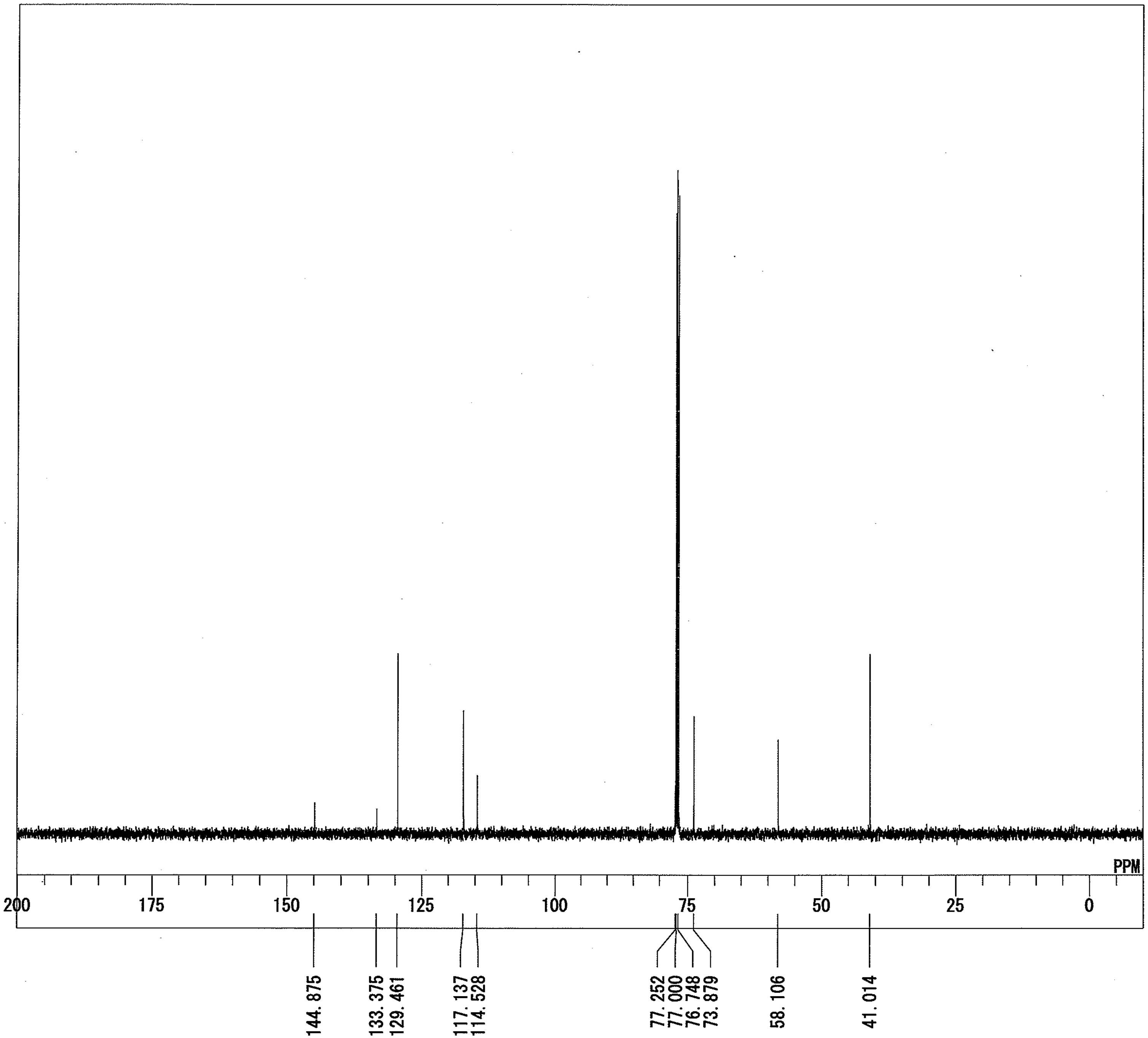

DFILE	NT-195-i-5-13C.als
COMNT	Single Pulse with Broadband Decoupling
DATIM	09-02-2012 03:25:39
OBNUC	13C
EXMOD	single_pulse_dec
OBFRQ	125.77 MHz
OBSET	7.87 KHz
OBFIN	4.21 Hz
POINT	32768
FREQU	31446.54 Hz
SCANS	507
ACQTM	1.0420 sec
PD	1.0000 sec
PW1	4.17 usec
IRNUC	1H
CTEMP	21.3 c
SLVNT	CDCL3
EXREF	77.00 ppm
BF	1.00 Hz
RGAIN	30

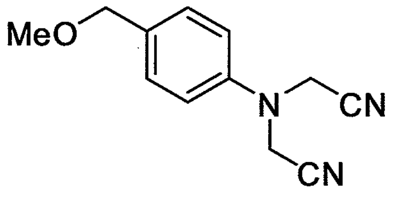

2j'

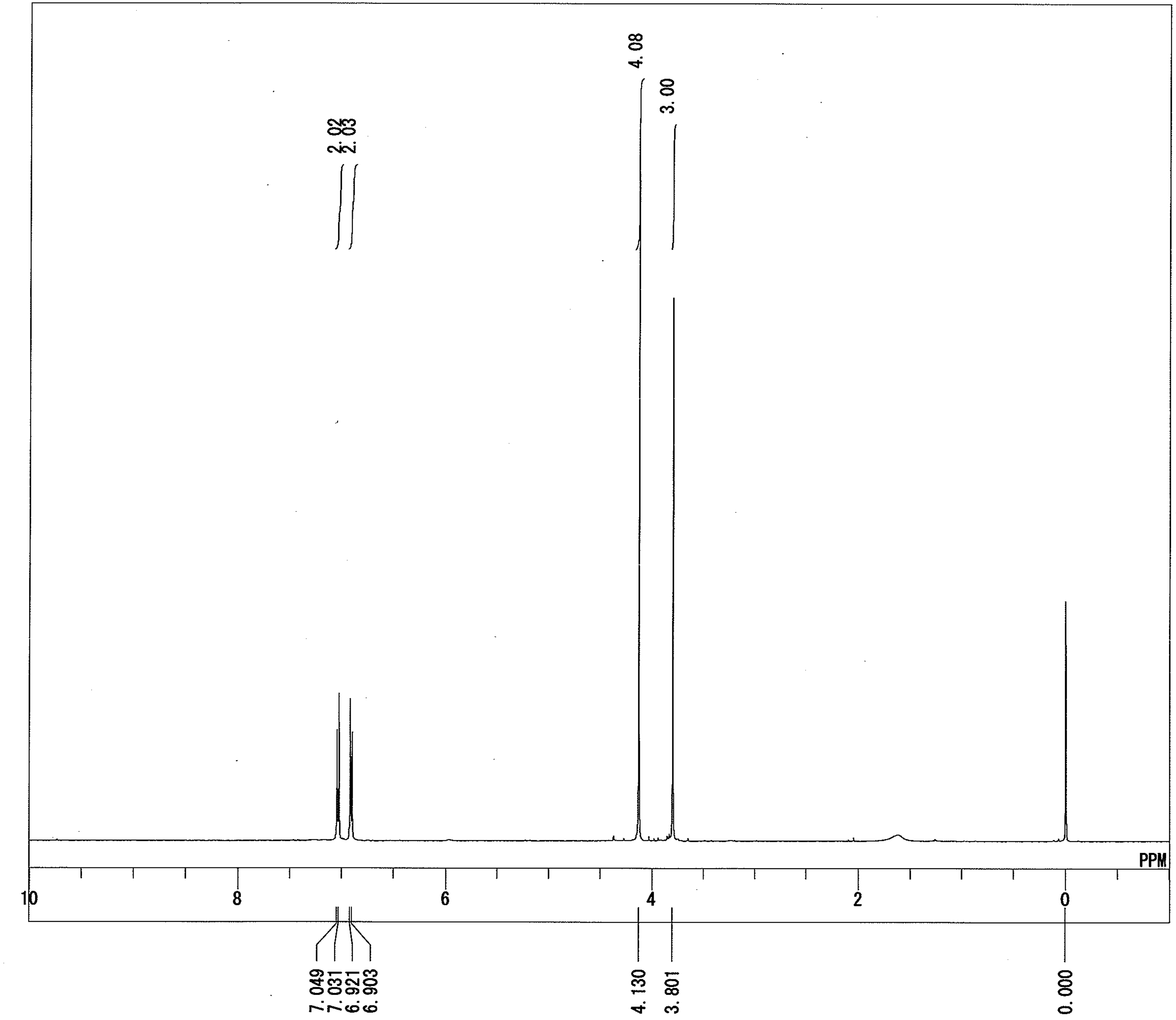

COMNT	Single Pulse Experiment
DATIM	01-09-2009 20:11:47
OBNUC	1H
EXMOD	single_pulse.exp
OBFRQ	500.16 MHz
OBSET	2.41 KHz
OBFIN	6.01 Hz
POINT	32768
FREQU	7507.51 Hz
SCANS	8
ACQTM	2.1823 sec
PD	4.0000 sec
PW1	7.00 usec
IRNUC	
CTEMP	23.3 c
SLVNT	CDCL3
EXREF	0.00 ppm
BF	0.23 Hz
RGAIN	21

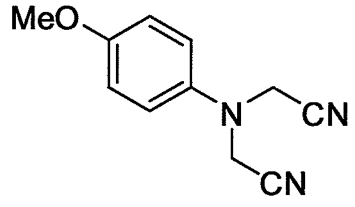

3a

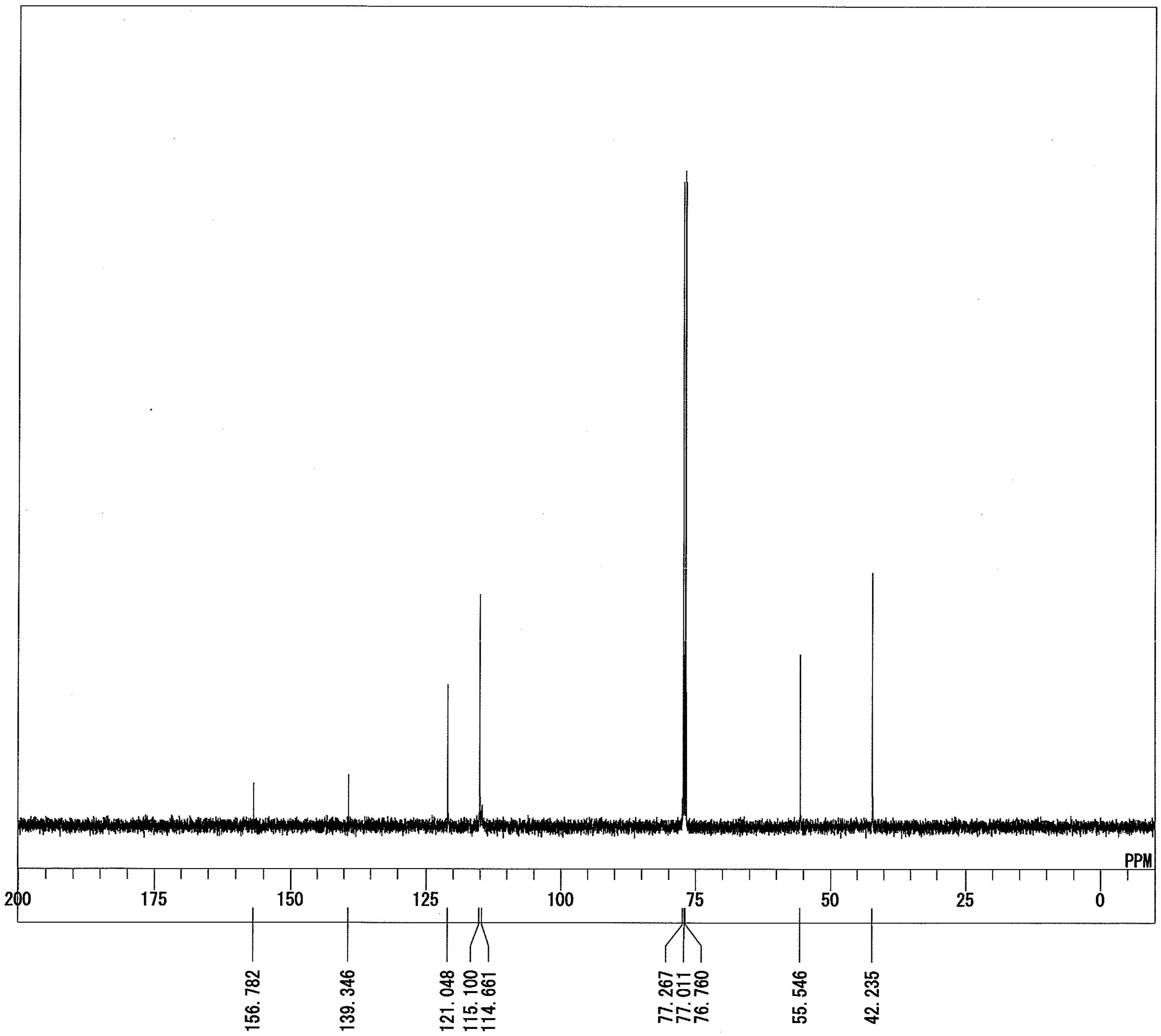

COMNT	Single Pulse with Broadband Decoupling
DATIM	10-03-2011 14:33:51
OBNUC	13C
EXMOD	single_pulse_dec
OBFRQ	125.77 MHz
OBSET	7.87 KHz
OBFIN	4.21 Hz
POINT	65536
FREQU	31446.54 Hz
SCANS	792
ACQTM	1.0420 sec
PD	1.0000 sec
PW1	4.17 usec
IRNUC	1H
CTEMP	24.1 c
SLVNT	CDCL3
EXREF	77.00 ppm
BF	1.00 Hz
RGAIN	30

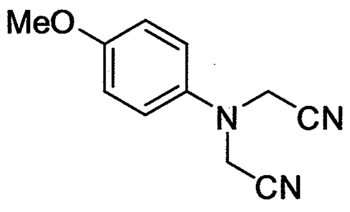

3a

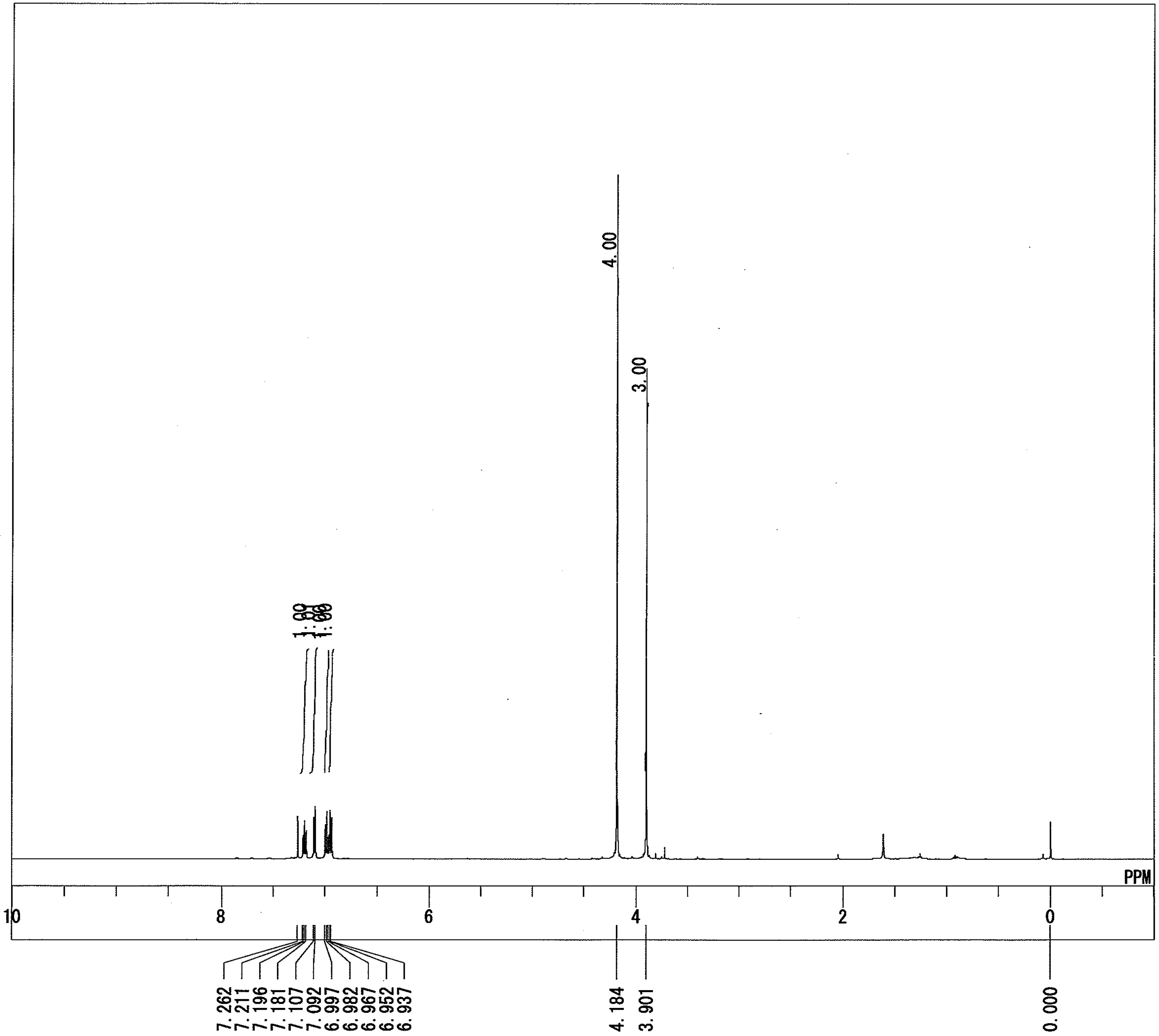

DFILE	NT-446-2-1H.als
COMNT	Single Pulse Experiment
DATIM	24-06-2013 13:37:18
OBNUC	1H
EXMOD	single_pulse.exp
OBFRQ	500.16 MHz
OBSET	2.41 KHz
OBFIN	6.01 Hz
POINT	32768
FREQU	7507.51 Hz
SCANS	8
ACQTM	2.1823 sec
PD	4.0000 sec
PW1	7.00 usec
IRNUC	
CTEMP	21.1 c
SLVNT	CDCL3
EXREF	0.00 ppm
BF	0.23 Hz
RGAIN	15

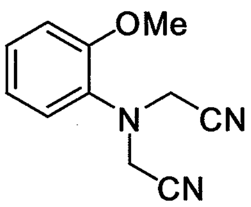

3b

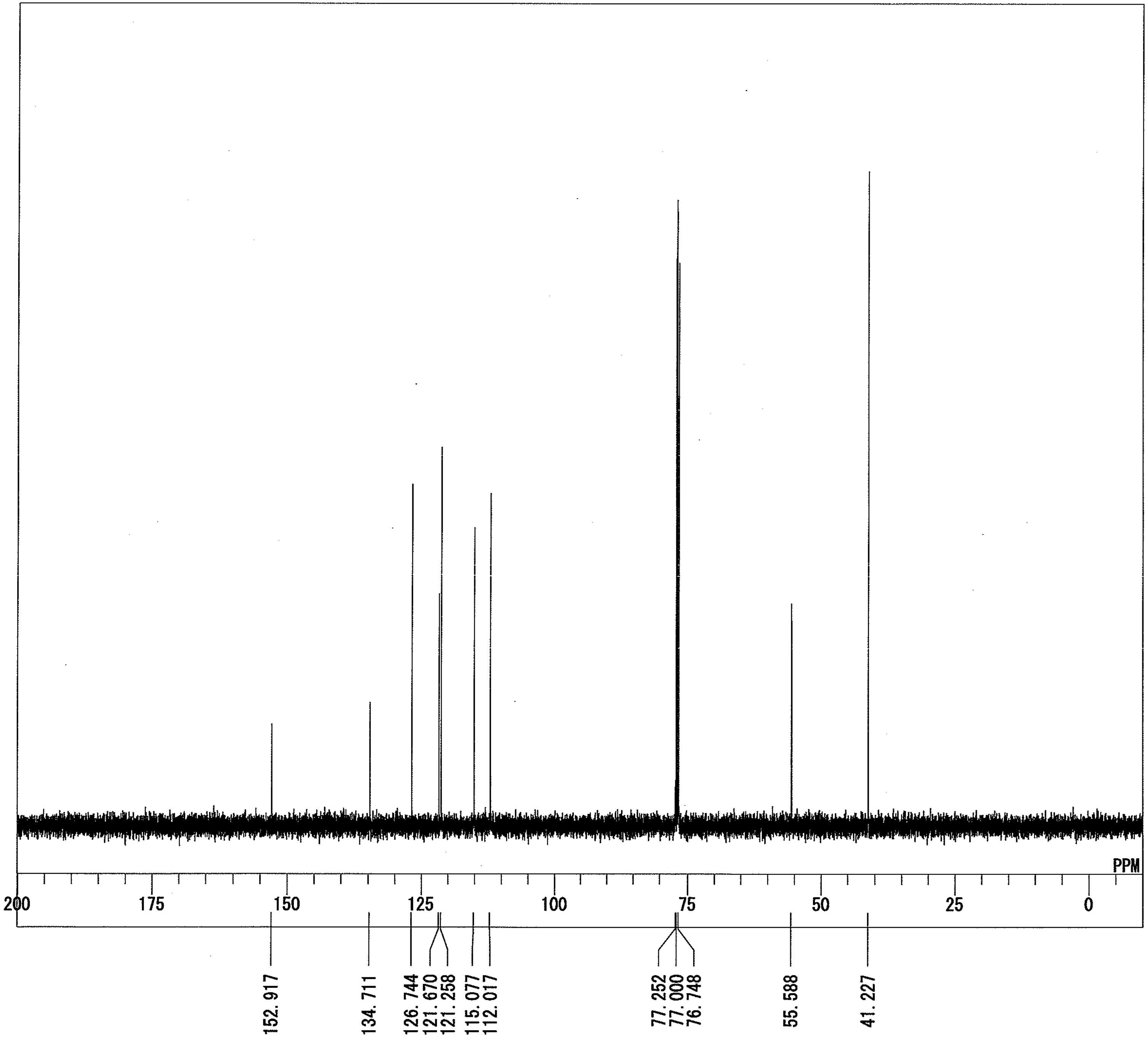

DFILE	NT-446-2-13C.als
COMNT	Single Pulse with Broadband Decoupling
DATIM	24-06-2013 13:49:22
OBNUC	13C
EXMOD	single_pulse_dec
OBFRQ	125.77 MHz
OBSET	7.87 KHz
OBFIN	4.21 Hz
POINT	32768
FREQU	31446.54 Hz
SCANS	329
ACQTM	1.0420 sec
PD	1.0000 sec
PW1	4.17 usec
IRNUC	1H
CTEMP	22.2 c
SLVNT	CDCL3
EXREF	77.00 ppm
BF	0.23 Hz
RGAIN	30

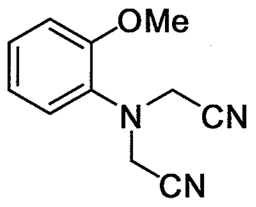

3b

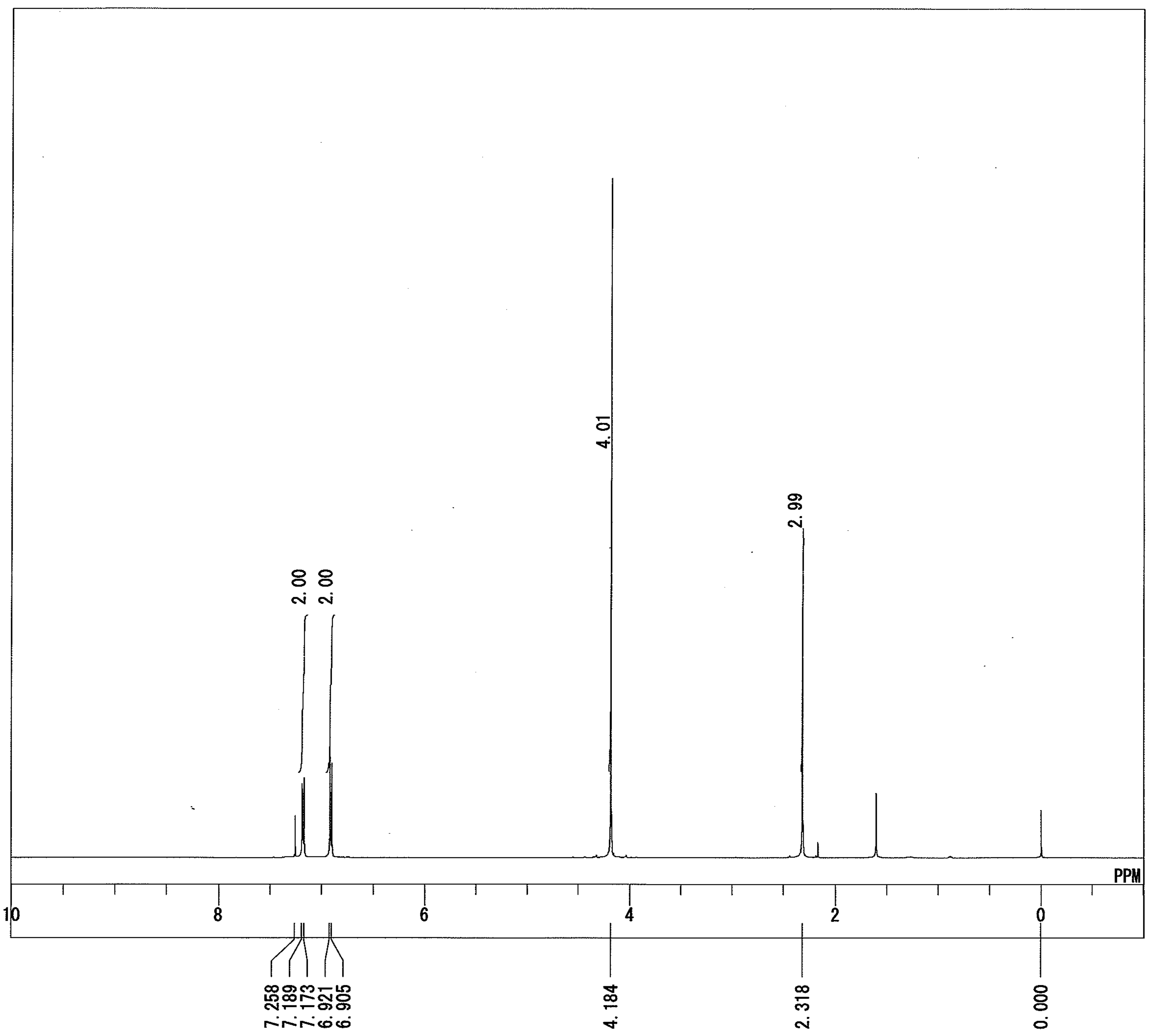

DFILE	NT-435-1H.als
COMNT	Single Pulse Experiment
DATIM	18-06-2013 18:58:28
OBNUC	1H
EXMOD	single_pulse.exp
OBFRQ	500.16 MHz
OBSET	2.41 KHz
OBFIN	6.01 Hz
POINT	32768
FREQU	7507.51 Hz
SCANS	8
ACQTM	2.1823 sec
PD	4.0000 sec
PW1	7.00 usec
IRNUC	
CTEMP	24.4 c
SLVNT	CDCL3
EXREF	0.00 ppm
BF	0.23 Hz
RGAIN	16

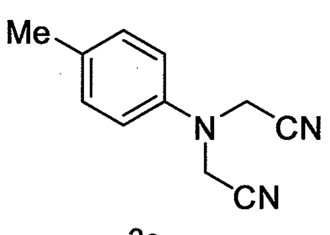

3c

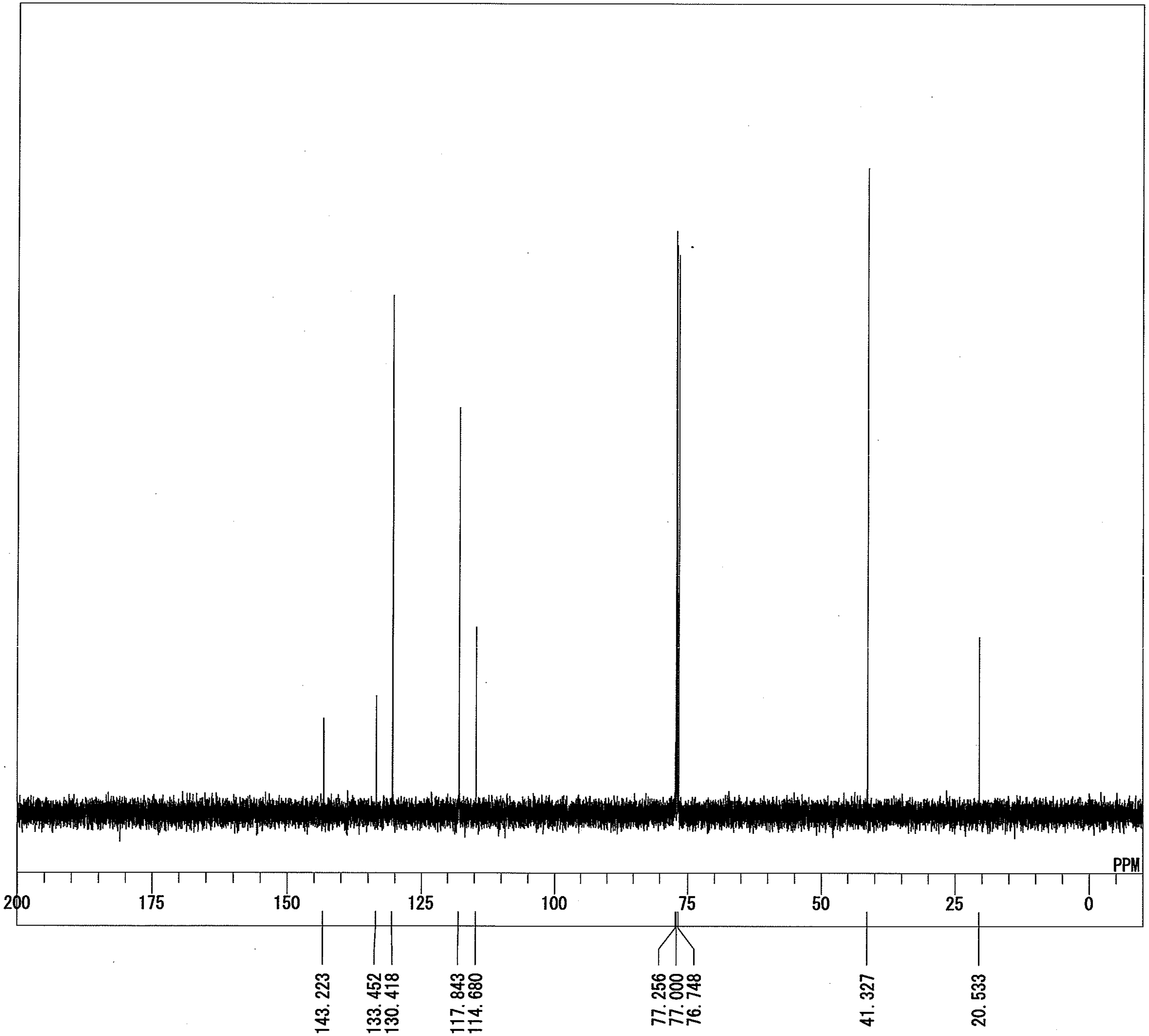

DFILE NT-435-13C_copy.1
COMNT Single Pulse with Broadband Decoupling
DATIM 18-06-2013 19:08:59
OBNUC 13C
EXMOD single_pulse_dec
OBFRQ 125.77 MHz
OBSET 7.87 KHz
OBFIN 4.21 Hz
POINT 65536
FREQU 31446.54 Hz
SCANS 156
ACQTM 1.0420 sec
PD 1.0000 sec
PW1 4.17 usec
IRNUC 1H
CTEMP 25.2 c
SLVNT CDCL3
EXREF 77.00 ppm
BF 0.48 Hz
RGAIN 30

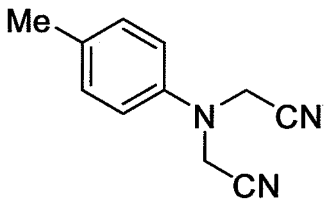

3c

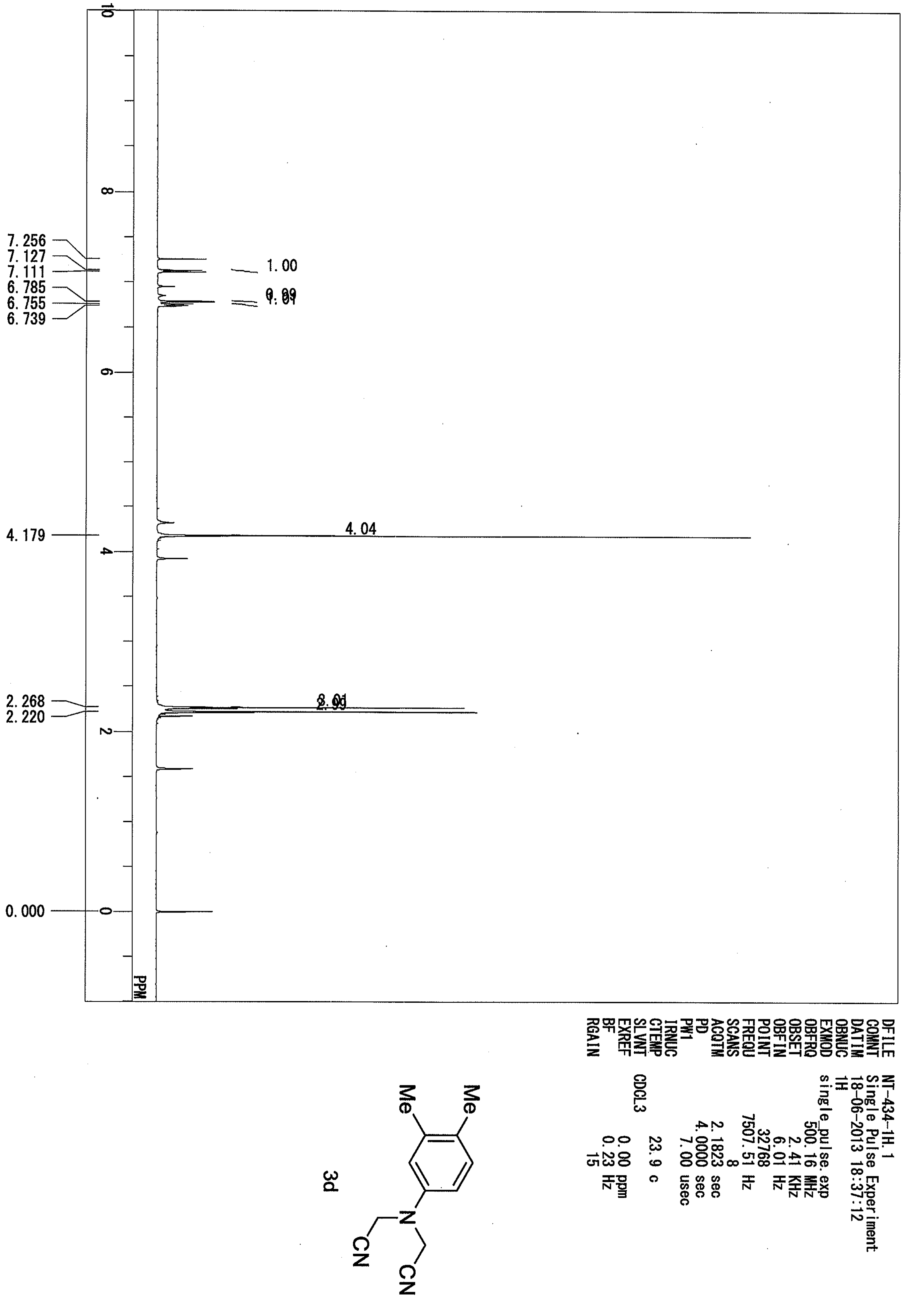
7.256
7.127
7.111
6.785
6.755
6.739
4.179
2.268
2.220
0.000
1.00
4.04
10
8
6
4
2
0
PPM
DFILE NT-434-1H.1
COMNT Single Pulse Experiment
DATIM 18-06-2013 18:37:12
OBNUC 1H
EXMOD single_pulse.exp
OBFRQ 500.16 MHz
OBSET 2.41 KHz
OBFIN 6.01 Hz
POINT 32768
FREQU 7507.51 Hz
SCANS 8
ACQTM 2.1823 sec
PD 4.0000 sec
PW1 7.00 usec
IRNUC
CTEMP 23.9 c
SLVNT CDCL3
EXREF 0.00 ppm
BF 0.23 Hz
RGAIN 15
Me
Me
N
CN
CN

3d

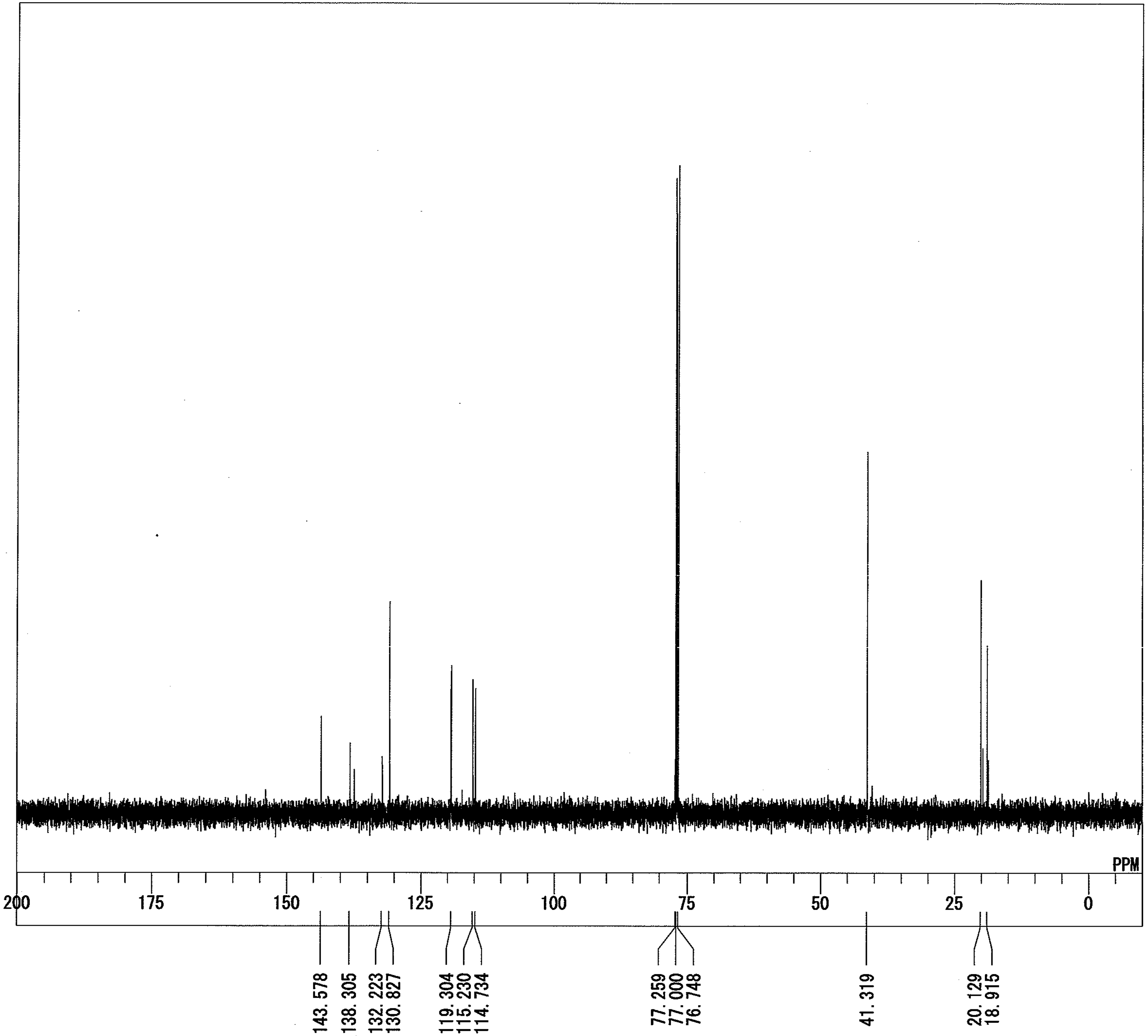

DFILE NT-434-13C.als
COMNT Single Pulse with Broadband Decoupling
DATIM 18-06-2013 18:50:33
OBNUC 13C
EXMOD single_pulse_dec
OBFRQ 125.77 MHz
OBSET 7.87 KHz
OBFIN 4.21 Hz
POINT 32768
FREQU 31446.54 Hz
SCANS 354
ACQTM 1.0420 sec
PD 1.0000 sec
PW1 4.17 usec
IRNUC 1H
CTEMP 25.3 c
SLVNT CDCL3
EXREF 77.00 ppm
BF 0.23 Hz
RGAIN 30

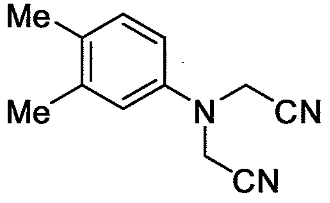

3d

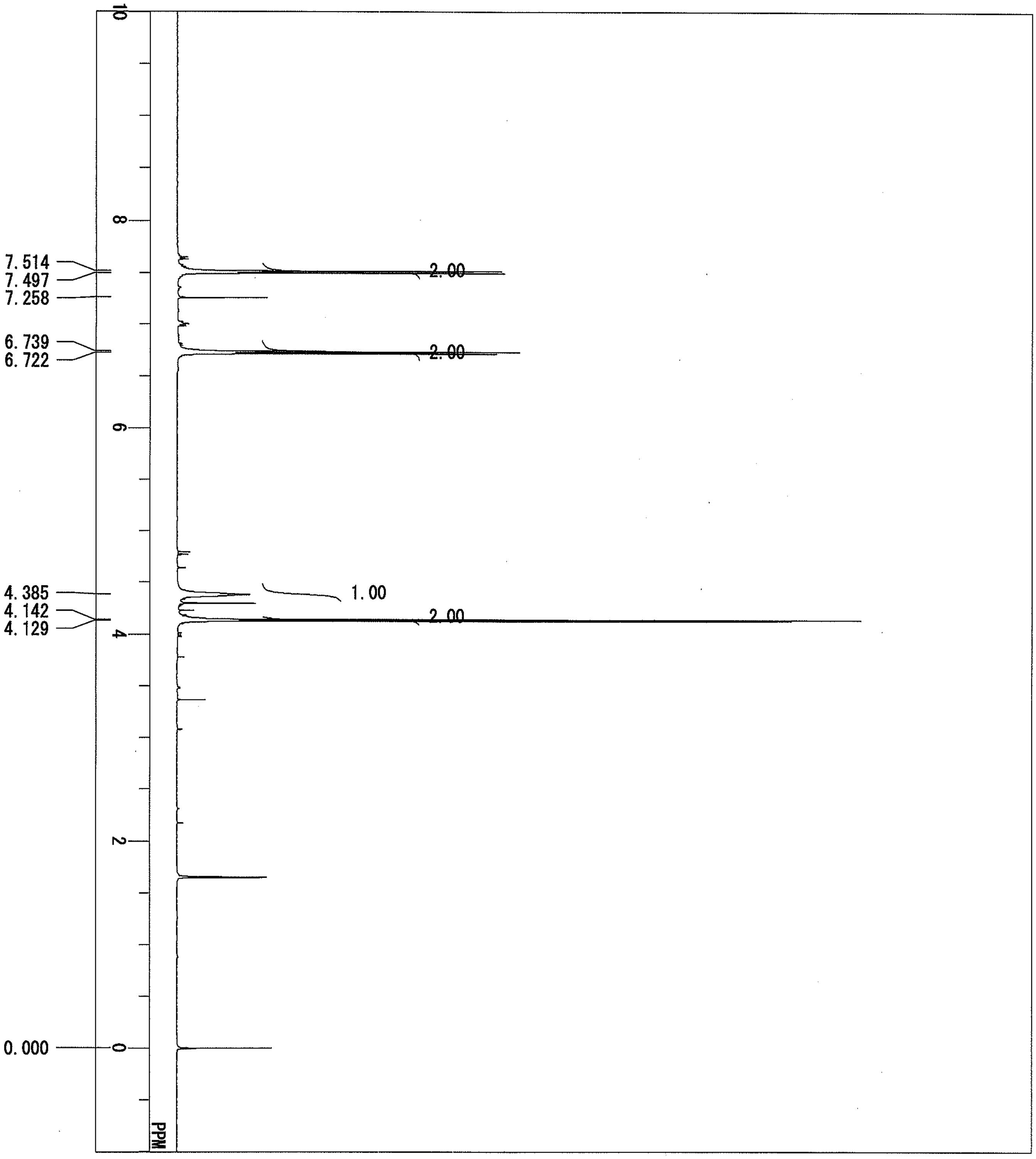

DFILE NT-436-rc-1H.als
COMNT Single Pulse Experiment
DATIM 24-06-2013 13:05:10
OBNUC 1H
EXMOD single_pulse.exp
OBFRQ 500.16 MHz
OBSET 2.41 KHz
OBFIN 6.01 Hz
POINT 32768
FREQU 7507.51 Hz
SCANS 8
ACQTM 2.1823 sec
PD 4.0000 sec
PW1 7.00 usec
IRNUC
CTEMP 21.3 c
SLVNT CDCL3
EXREF 0.00 ppm
BF 0.23 Hz
RGAIN 15

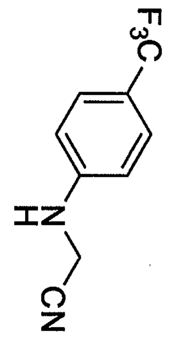

4a

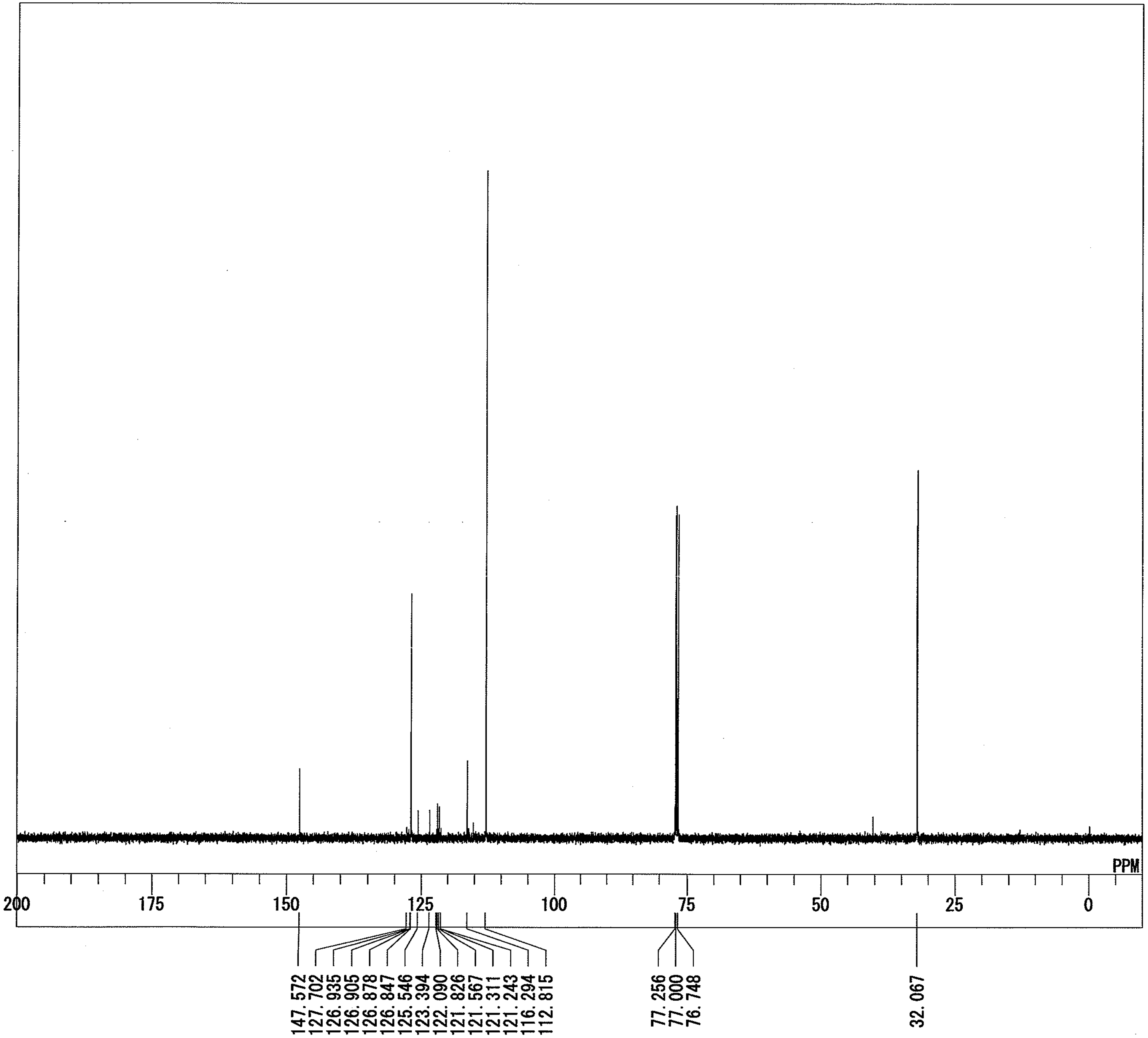

DFILE	NT-436-rc-13C_copy.als
COMNT	Single Pulse with Broadband Decoupling
DATIM	24-06-2013 13:29:45
OBNUC	13C
EXMOD	single_pulse_dec
OBFRQ	125.77 MHz
OBSET	7.87 KHz
OBFIN	4.21 Hz
POINT	65536
FREQU	31446.54 Hz
SCANS	626
ACQTM	1.0420 sec
PD	1.0000 sec
PW1	4.17 usec
IRNUC	1H
CTEMP	22.2 c
SLVNT	CDCL3
EXREF	77.00 ppm
BF	0.48 Hz
RGAIN	30

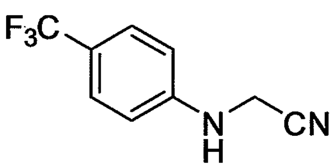

4a

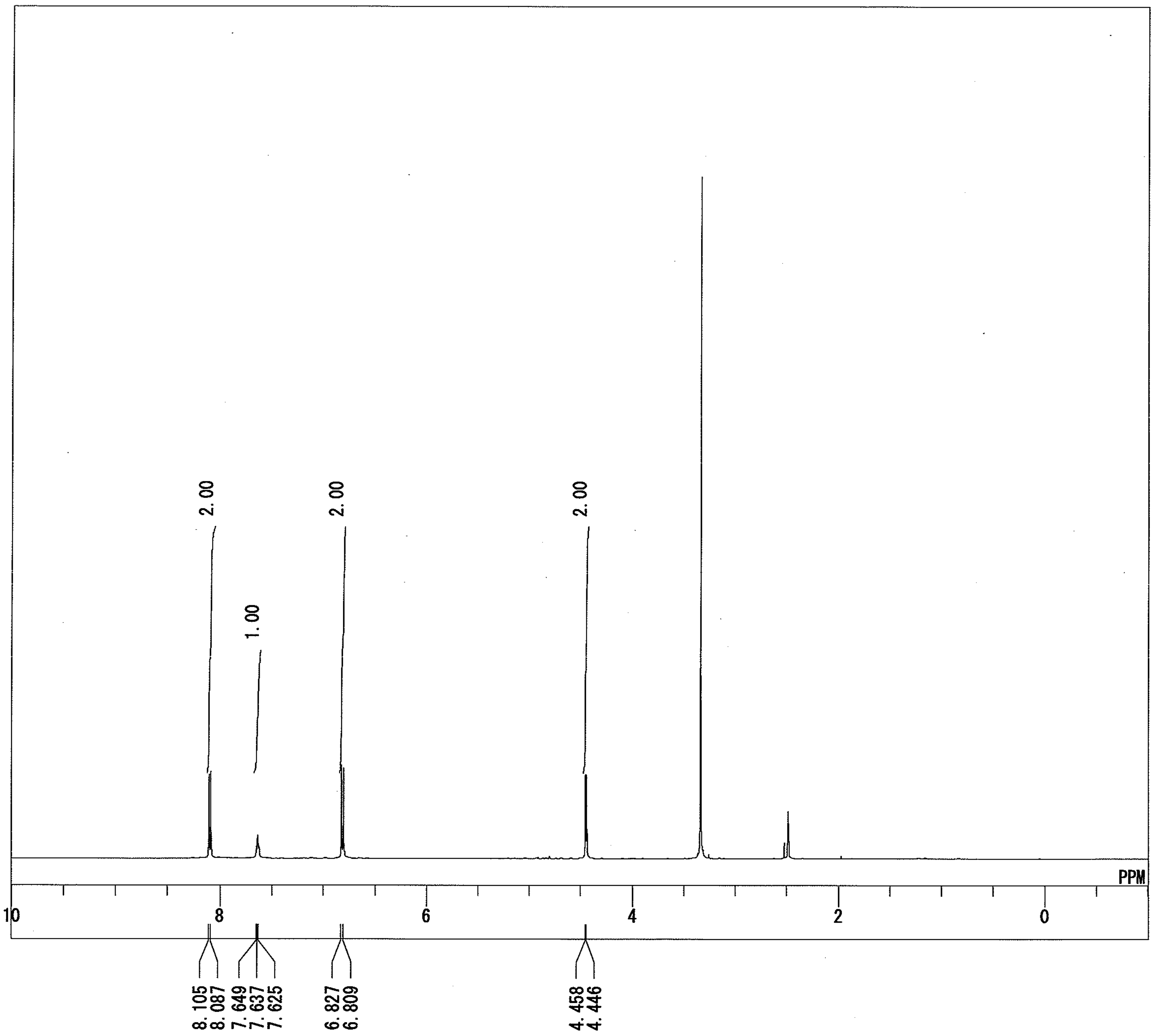
2.00
1.00
2.00
2.00
PPM
10
8
6
4
2
0
8.105
8.087
7.649
7.637
7.625
6.827
6.809
4.458
4.446
DFILE NT-pNO2-DMSO-2-1H.als
COMNT Single Pulse Experiment
DATIM 08-07-2013 10:43:21
OBNUC 1H
EXMOD single_pulse.exp
OBFRQ 500.16 MHz
OBSET 2.41 KHz
OBFIN 6.01 Hz
POINT 32768
FREQU 7507.51 Hz
SCANS 8
ACQTM 2.1823 sec
PD 4.0000 sec
PW1 7.00 usec
IRNUC
CTEMP 23.5 c
SLVNT DMSO
EXREF 2.49 ppm
BF 0.23 Hz
RGAIN 17

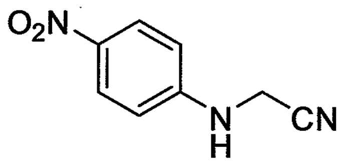
O2N
N
H
CN

4b

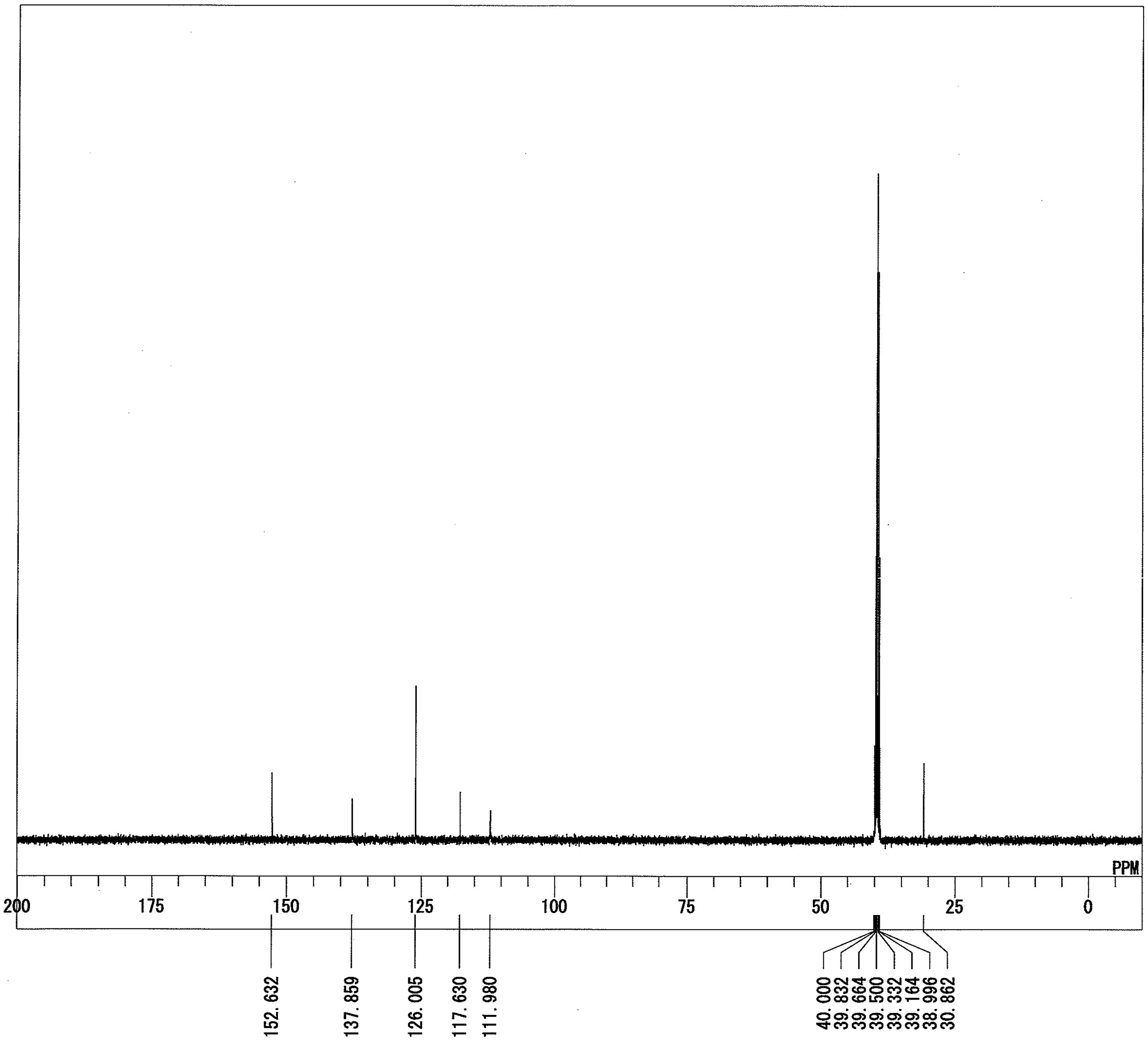

DFILE	NT-pNO2-DMSO-2-13C.als
COMNT	Single Pulse with Broadband Decoupling
DATIM	08-07-2013 11:10:55
OBNUC	13C
EXMOD	single_pulse_dec
OBFRQ	125.77 MHz
OBSET	7.87 KHz
OBFIN	4.21 Hz
POINT	65536
FREQU	31446.54 Hz
SCANS	727
ACQTM	1.0420 sec
PD	1.0000 sec
PW1	4.17 usec
IRNUC	1H
CTEMP	25.1 c
SLVNT	DMSO
EXREF	39.50 ppm
BF	0.48 Hz
RGAIN	30

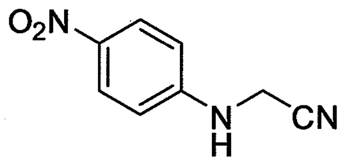

4b

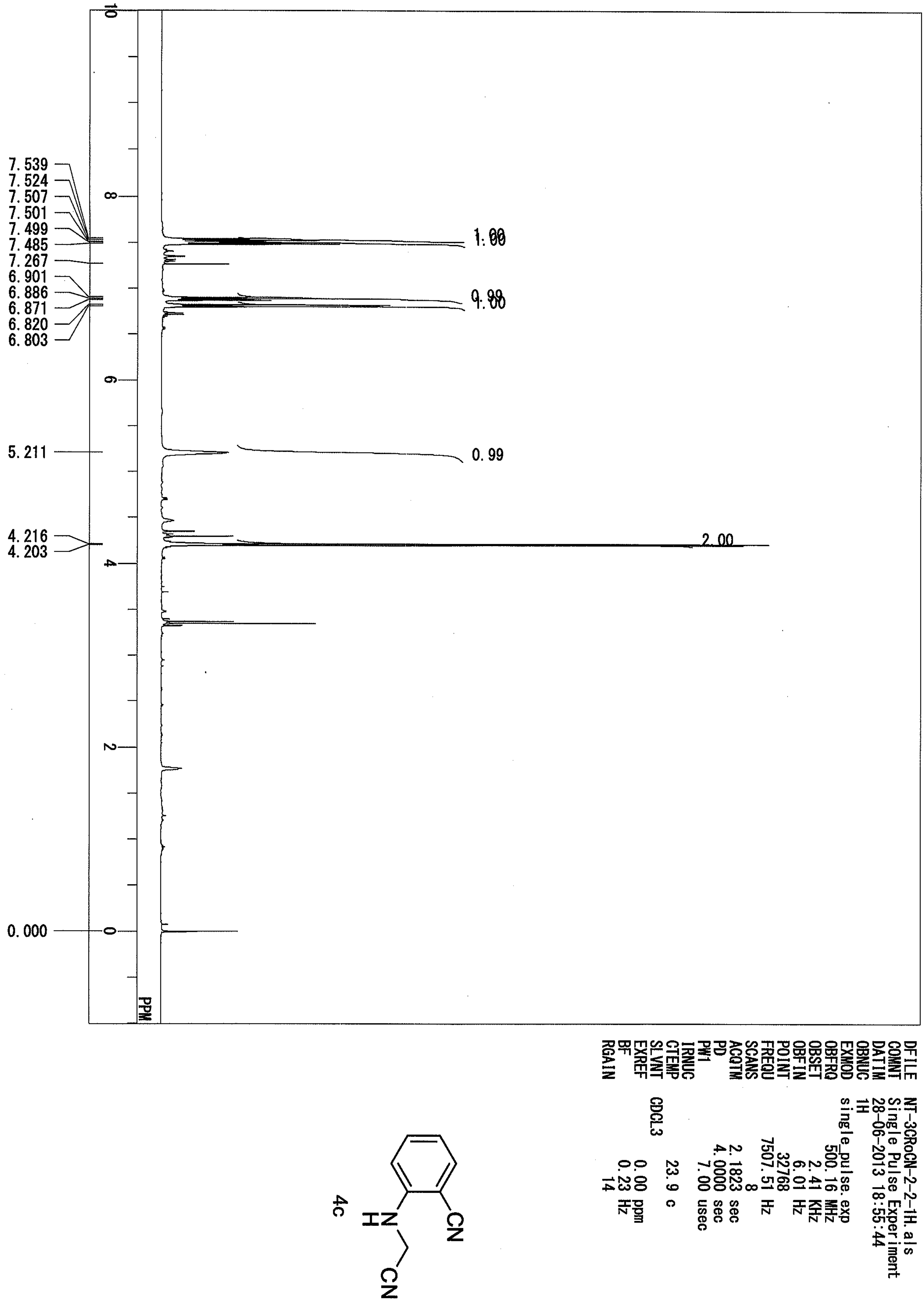
7.539
7.524
7.507
7.501
7.499
7.485
7.267
6.901
6.886
6.871
6.820
6.803
5.211
4.216
4.203
0.000
1.00
1.00
0.99
1.00
0.99
2.00
PPM
DFILE NT-3CRoCN-2-2-1H.als
COMNT Single Pulse Experiment
DATIM 28-06-2013 18:55:44
OBNUC 1H
EXMOD single_pulse.exp
OBFRQ 500.16 MHz
OBSET 2.41 KHz
OBFIN 6.01 Hz
POINT 32768
FREQU 7507.51 Hz
SCANS 8
ACQTM 2.1823 sec
PD 4.0000 sec
PW1 7.00 usec
IRNUC
CTEMP 23.9 c
SLVNT CDCL3
EXREF 0.00 ppm
BF 0.23 Hz
RGAIN 14
CN
N
H
CN
4c

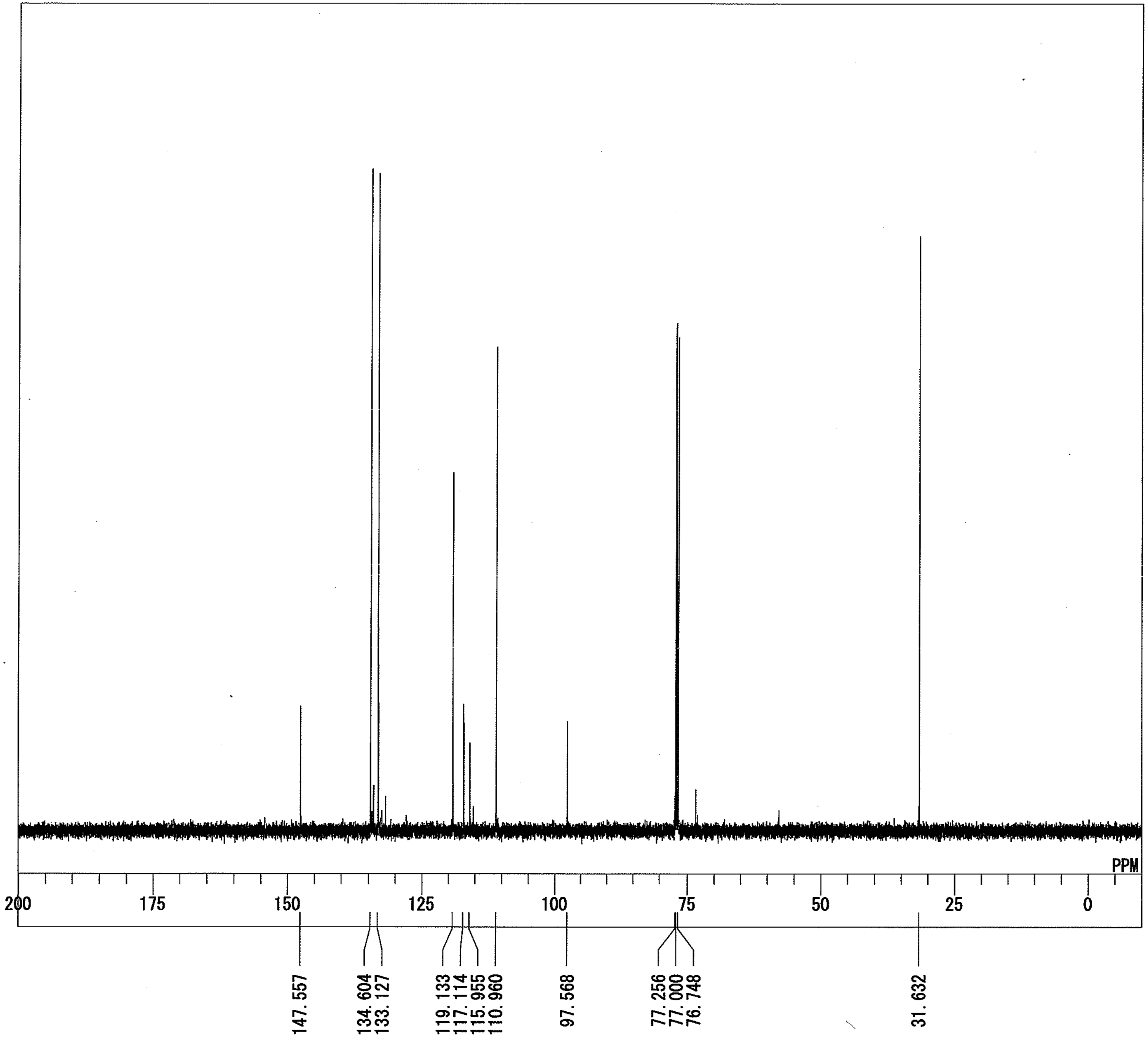

DFILE NT-3CRoCN-2-2-13C.als
COMNT Single Pulse with Broadband Decoupling
DATIM 28-06-2013 19:16:14
OBNUC 13C
EXMOD single_pulse_dec
OBFRQ 125.77 MHz
OBSET 7.87 KHz
OBFIN 4.21 Hz
POINT 65536
FREQU 31446.54 Hz
SCANS 524
ACQTM 1.0420 sec
PD 1.0000 sec
PW1 4.17 usec
IRNUC 1H
CTEMP 24.8 c
SLVNT CDCL3
EXREF 77.00 ppm
BF 0.48 Hz
RGAIN 30

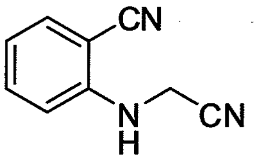

4c
